# The Borderline Symptom List–Interview: development and psychometric evaluation of an observer-based instrument for assessing symptom severity in borderline personality disorder

**DOI:** 10.1186/s40479-025-00310-6

**Published:** 2025-08-28

**Authors:** Büsra Senyüz, Ruben Vonderlin, Carola Claus, Saskia Mahalingam, Stefan Koch, Ulrich Voderholzer, Tobias Teismann, Nikolaus Kleindienst, Jan R. Böhnke, Stefanie Lis, Tali Boritz, Shelley McMain, Martin Bohus

**Affiliations:** 1 Department of Psychosomatic Medicine and Psychotherapy, Central Institute of Mental Health, Medical Faculty Mannheim, Heidelberg University, J5, D-68159 Mannheim, Germany; 2 German Center for Mental Health (DZPG), Partner Site Mannheim - Heidelberg - Ulm, Mannheim, Germany; 3 Mental Health Research and Treatment Center, Ruhr-University Bochum, Bochum, Germany; 4 Schoen Clinic Roseneck, Prien am Chiemsee, Germany; 5 Clinic for Psychiatry and Psychotherapy, University Hospital of Munich, Munich, Germany; 6 School of Health Sciences, University of Dundee, Dundee, UK; 7 Department of Public Mental Health, Central Institute of Mental Health, Medical Faculty Mannheim, Heidelberg University, Mannheim, Baden-Württemberg, Germany; 8 Department of Clinical Psychology, Central Institute of Mental Health, Medical Faculty Mannheim, Heidelberg University, Mannheim, Germany; 9 Department of Psychology, York University, Toronto, ON Canada; 10 Centre for Addiction and Mental Health, Borderline Personality Disorder Clinic, Toronto, ON Canada; 11 Department of Psychiatry, University of Toronto, Toronto, Canada; 12 McLean Hospital, Harvard Medical School, Boston, MA USA

**Keywords:** Borderline personality disorder (BPD), Severity assessment, Semi-Structured interview, Psychometric evaluation

## Abstract

**Background:**

Borderline Personality Disorder (BPD) is a complex mental health condition characterized by pervasive instability in mood, interpersonal relationships, self-concepts, and behavior. A reliable assessment of BPD symptom severity is essential for effective treatment planning and evaluation. This study introduces and evaluates the Borderline Symptom List Interview (BSL-I), a semi-structured interview designed to assess the severity of BPD symptoms comprehensively.

**Method:**

The BSL-I is a freely accessible 31-item interview designed to assess BPD symptom severity. It evaluates (a) the frequency and subjective distress associated with BPD-specific and typical psychopathological symptoms, (b) the behavioral consequences of these symptoms, (c) functional impairment, and (d) facets of positive mental health. The items were developed through an iterative process, incorporating feedback from international experts and individuals with lived experience of BPD. Psychometric properties of the BSL-I were examined cross-sectionally in different samples of clients meeting DSM-5 criteria for BPD (*n* = 171), clinical controls (*n* = 89), and healthy controls (*n* = 43).

**Results:**

The BSL-I demonstrates good internal consistency within the BPD sample (Cronbach’s α = 0.82) and good interrater reliability (ICC = 0.768). It significantly discriminates between BPD clients and clinical controls (Cohen’s d = 2.02) and healthy controls (Cohen’s d = 3.88). High correlations were observed with other established BPD symptom measures, including the number of IPDE criteria (*r* = 0.70, *p* < 0.001) and the BSL-23 (*r* = 0.83, *p* < 0.001).

**Discussion:**

Our findings indicate that the BSL-I is a reliable and valid multidimensional instrument for assessing the severity of BPD. Both clinical experts and clients found the application of the BSL-I acceptable and feasible. Future research might explore its sensitivity to change resulting from psychosocial treatments and assess its utility for treatment planning and outcome measurement.

**Conclusion:**

The BSL-I is a practical and psychometrically sound instrument for assessing the severity of BPD symptoms in clinical and research contexts.

**Supplementary Information:**

The online version contains supplementary material available at 10.1186/s40479-025-00310-6.

## Introduction

Borderline Personality Disorder (BPD) is a severe and often chronic psychiatric disorder characterized by wide-ranging instability in mood, relationships, self-image, and behavior. The lifetime prevalence is estimated to be around 0.7–2.7% of the population [1]. This condition often causes significant psychosocial impairments that affect all aspects of an individual’s life, such as work, education, and social connections, which can lead to reduced overall life satisfaction for those affected [2].

Alongside clinical diagnostic instruments, such as the SCID-5-PD [3] and IPDE [4], which primarily focus on determining whether individuals fulfill the criteria for BPD, severity instruments are essential for capturing the extent and complexity of the disorder. Research indicates that simply counting diagnostic criteria fails to provide a comprehensive assessment of symptom severity, as it overlooks differences in psychosocial functioning [5]. Furthermore, these diagnostic instruments are not designed to track the frequency, distress, or changes in BPD symptoms over time—dimensions that severity instruments typically aim to assess.

According to the conceptualization of personality disorders in the ICD-11 [6], severity indicates the overall level of personality dysfunction, characterized by impairments in self- and interpersonal functioning. It is categorized into three levels—mild, moderate, and severe—based on the degree of impairment. Transitioning from a categorical to a dimensional view of personality disorders, BPD is seen as a pattern within the five trait domain qualifiers characterized by Negative Affectivity, Detachment, Disinhibition, Dissociality, and Anankastia, with further specification through the BPD pattern qualifier.

The DSM-5 Alternative Model for Personality Disorders (AMPD [7]), also employs a dimensional framework. Criterion A assesses the severity of personality dysfunction (both self- and interpersonal functioning), Criterion B evaluates maladaptive traits across five broad domains: Negative Affectivity, Detachment, Antagonism, Disinhibition, and Psychoticism, which can be further differentiated into 25 specific trait facets. Similar to the ICD-11 [6], BPD is viewed as a particular configuration of personality traits. Importantly, in both models, severity reflects overall personality psychopathology rather than being specific to the BPD pattern or qualifier.

In this context, a reliable assessment of BPD symptom severity that extends beyond the limited number of diagnostic criteria, would offer several advantages and valuable implications for both treatment and research. First, severity assessments also take a dimensional rather than a categorical approach, allowing for the measurement of varying degrees of severity both above and below diagnostic thresholds [8]. Such measures aim to quantify a continuous spectrum (“dimension”) of symptom severity, in contrast to the trait measures mentioned above that aim to differentiate trait dimensions of personality psychopathology. Second, these instruments facilitate the tracking of changes in symptomatology, providing a more nuanced picture of treatment effectiveness in psychotherapy research and clinical practice. Third, severity measures can further support clinical decision-making, treatment selection, and planning. Additionally, severity assessments can incorporate aspects of psychosocial functioning, including work capacity, disability status, and eligibility for public assistance, thereby enhancing the understanding of the real-world consequences of symptoms [9].

Psychometric instruments used to assess symptom severity can be categorized into two main types: self-report and observer-based instruments. Two well-established self-report instruments for assessing BPD severity are the Borderline Symptom List-23 (BSL-23) [10] and the Borderline Evaluation of Severity Over Time (BEST) [11]. The BSL-23 is a widely used self-report instrument that has been translated into 18 languages, demonstrating consistent and good psychometric properties across languages [12–14]. The BSL-23 goes beyond the diagnostic criteria of the DSM-5, incorporating additional evidence-based borderline-typical symptoms. These include feelings of shame [15, 16], aversive inner tension [17], self-hate [18, 19], and the perception of voices and sounds [20, 21]. Moreover, the BSL-23 already includes symptoms outlined in the newly proposed additional criteria of the borderline qualifier in the ICD-11 [6], such as negative self-perception as disgusting and contemptible, pervasive loneliness, and problems establishing or maintaining trust in interpersonal relationships [20]. The 23 items of the BSL-23 are rated on a 0–4 Likert scale based on the level of suffering experienced from symptoms during the past week.

The BEST [11] measures severity in three dimensions: (a) thoughts and feelings, (b) negative behavior, and (c) positive behavior. Whereas the first two scales are based exclusively on the DSM-IV criteria of BPD, the positive behavior scale also includes positive coping mechanisms such as skillful behavior. Symptoms are rated on a 1-5-point Likert scale, reflecting the degree to which they cause distress, difficulties in relationships, and/or interfere with accomplishing tasks. The reference period can be set for the last seven days, 30 days, or freely chosen. The instrument does not explicitly differentiate subjective distress, relational problems, or general impairment. A limitation of this instrument is that only the DSM-IV criteria are considered at the symptom level.

Although self-report measures of severity provide valuable insights into individuals’ subjective experiences and symptom changes over time, their use with clients with BPD introduces potential issues. These issues arise from the subjective nature of self-report instruments, which rely on the individuals’ self-perception and current emotional state, factors that can introduce systematic biases [22, 23]. Individuals with BPD may over- or underestimate the severity of their symptoms depending on their emotional state at the time of reporting. This is particularly relevant in BPD populations, where both self-perception and emotional states can be highly variable, fluctuating within hours. (e.g [24]). Moreover, studies have documented discrepancies between self-reported depression symptoms and clinical ratings in individuals with BPD [22], highlighting the importance of multimethod assessment approaches to obtain a more comprehensive understanding of symptomatology. Therefore, clinical instruments serve as valuable complementary tools for clinicians to assess symptoms from an observer’s perspective [25]. While observer-based instruments also rely on self-reports, they incorporate evaluative components that allow clinicians to clarify symptom details, provide examples, and contextualize the information across various clients. These instruments enable clinicians to leverage their expertise and judgment to compare the reported symptoms within the disorder population and verify their accuracy.

The two currently best-established observer-based severity instruments for assessing BPD severity are the Borderline Personality Disorder Severity Index (BPDSI-IV) [26] and the Zanarini Rating Scale for BPD (ZAN-BPD) [27]. Both instruments assess the nine DSM-IV diagnostic criteria for BPD over a specific period, typically through multiple items per criterion. While the BPDS-I [25] operationalizes the severity solely based on the frequency of symptoms, the ZAN-BPD [27] also incorporates symptom intensity in its rating criteria for assessors. Both instruments have the advantage of integrating clinical judgment and are widely used in research.

However, both the BPDS-I [26] and ZAN-BPD [27] rely exclusively on DSM-IV criteria, which limits their ability to capture the evidence-based symptoms of the borderline qualifier as newly introduced by the ICD-11 [6]. These qualifier symptoms are (a) view of self as inadequate, bad, guilty, disgusting, and contemptuous; (b) a profound sense of being different and isolated from others; (c) a sense of alienation and pervasive loneliness; (d) proneness to rejection sensitivity; (e) problems in establishing and maintaining consistent levels of trust in interpersonal relationships; and (f) frequent misinterpretation of social signals.

Notably, existing severity instruments operationalize BPD severity in conceptually different ways. Whereas the BEST [11] captures the distress and the problems caused by symptoms and incorporates positive coping mechanisms, the BSL-23 [10] focuses on the suffering caused by symptoms. The BPDS-I [26] focuses on frequency alone, whereas the ZAN-BPD [27] combines frequency and symptom intensity. However, each of these instruments only focuses on one of these aspects or integrates both information in one rating, potentially leading to a loss of information. For instance, it becomes challenging to differentiate the severity of a single episode of severe self-harm from multiple episodes of mild self-harm. Thus, there is a need for a nuanced approach to assessing BPD severity that captures its multidimensional nature.

Additionally, although the BSL-23 [10] is widely used, there is no corresponding observer-based instrument, and the existing observer-based instruments for assessing BPD severity differ conceptually from the BSL-23. This further highlights the need for a corresponding observer-based severity instrument that complements the BSL-23 and aligns with its conceptual framework.

Given the limitations of existing instruments in assessing BPD severity and the need for an adapted conceptual framework, we developed a multidimensional semi-structured interview, the Borderline Symptom List-Interview (BSL-I), based on the well-established BSL-23 questionnaire [10]. To ensure the dissemination of this instrument and guarantee that emerging countries with limited resources also have access to well-operationalized measuring instruments, the BSL-I will be freely accessible.

The BSL-I was designed to provide a comprehensive assessment of BPD severity, extending beyond diagnostic criteria and to capture the broader psychosocial and functional impacts of the disorder. This comprehensive assessment follows a multidimensional approach, evaluating the frequency of symptoms, subjective distress, behavioral consequences, and impairment in daily life.

To catch the full range of symptoms, we also included items assessing facets of positive mental health. Recognizing its potential as a resource for alleviating the overall symptom burden and indicating recovery, we included items assessing overarching constructs such as hope and confidence [28, 29], meaningfulness [30], life satisfaction [31], joy or happiness [32], and security and comfort [33].

Alongside symptoms of BPD, the BSL-I systematically evaluates their impact on psychosocial functioning, addressing impairments across three key life domains: daily practical skills, social relationships, and work or education [2]. Assessing functioning in these domains is crucial for understanding the real-world impact of BPD symptoms, as functional impairments often persist even after symptom remission through therapy [31]. Thus, the BSL-I captures not only symptom severity but also the broader functional consequences of the disorder.

In alignment with the ICD-11’s [6] comprehensive understanding of BPD, this study has two primary objectives: (1) to provide a multidimensional, time-efficient, and openly accessible assessment tool for BPD severity, and (2) to evaluate its psychometric properties. We hypothesize that the BSL-I functions as a reliable and valid instrument that complements the BSL-23. By achieving these objectives, we aim to contribute a practical instrument that enhances the precision and consistency of BPD severity assessments.

## Methods

### Item generation and development

The development of the BSL-I was initially conducted in German and followed a theoretical-deductive and empirical approach comprising five steps. In Step 1, an initial set of 43 items was developed based on the established BSL-23 [10], the ICD-11 [6], consultations among the author team, which comprises clinical and research specialists in BPD, namely M.B. and R.V., a statistician, N.K., and a PhD student, B.S., along with an extensive literature review to assess the proposed items. In Step 2, feedback was obtained from17 international experts in BPD research and treatment, as well as individuals with lived experience, to assess the face and content validity of the initial 43 items. Out of 38 experts invited, 17 replied during the specified period from April 25 to May 8, 2023. The expert panel included members of the World DBT Association and clinicians from the Department of Psychosomatic Medicine and Psychotherapy at the Central Institute of Mental Health. Individuals with lived experience were recruited through the German Borderline Trialog [34] initiative. For non-German-speaking experts, the item set was translated using DeepL [35] and subsequently revised by the authors mentioned above to ensure clarity and conceptual accuracy. All experts were asked to rate each item in terms of its relevance to the construct of BPD severity (1 = very important, 6 = not important at all) and clarity (1 = very clear, 6 = completely unclear). Additional qualitative feedback was encouraged to refine item wording and conceptual alignment further. In Step 3, the initial 43 items were reduced to 31 based on the feedback regarding the importance of each item. The importance ratings were calculated by averaging all ratings for each item. Items with an average importance rating above 3 (somewhat important) were excluded based on the ratings and subsequent discussions with the authors mentioned above. In Step 4, the feasibility of the items was tested in pivotal clinical assessments, involving evaluations by two assessors with six clients in an inpatient setting at the Central Institute of Mental Health. In Step 5, verbal feedback was gathered from the assessors and clients after the pivotal assessments, and based on this feedback, the final set of items was selected. Overall, both assessors and clients found the interview feasible in terms of content and duration. The average length of the interview was 45 min. The final structure of the BSL-I, comprising 31 items, four dimensions, and the calculation of the scale score, is presented in Table 1.

### Training of the assessors

To train the assessors in the reliable administration of the BSL-I, all interviewers underwent a four-hour training consisting of three phases: In the first phase (1.5 hours), the conduct of the interview and the individual items were discussed. In the second phase (1 hour), the assessors concurrently rated a ‘gold standard interview’ conducted by one of the authors with a client. The ratings were compared during the third training phase, which lasted 1.5 h, and any deviations and questions were discussed and addressed. A minimum of a bachelor’s degree in psychology or a related healthcare discipline was required to conduct the interviews.

### Participants

The interview was conducted with 303 German-speaking individuals, comprising 171 clients meeting ≥ 5 DSM-5 criteria for BPD (BPD), 89 clinical controls (CC), and 43 healthy controls (HC) cross-sectionally. The BPD clients were recruited from a waitlist at the Central Institute of Mental Health in Mannheim, the Ruhr University in Bochum, and the University Medical Hospital Schleswig-Holstein in Kiel. The inclusion criteria required participants to be between 18 and 65 years old and to meet at least five criteria for BPD, as defined by the DSM-5. Exclusion criteria included: (a) a diagnosis of bipolar I disorder, (b) current or lifetime psychosis, and (c) a body mass index (BMI) below 17.5. Clients underwent a comprehensive diagnostic evaluation using the International Personality Disorder Examination - Borderline section (IPDE [4]), and the Mini-International Neuropsychiatric Interview (M.I.N.I.; [37]). The IPDE [4] and M.I.N.I [37] were administered by the same assessors who conducted the BSL-I. To evaluate the interrater reliability of the BSL-I, 80 clients with BPD agreed to be interviewed twice by two independent assessors.

The clinical control group was recruited at the Schön Clinic Roseneck in Prien, Germany. Ninety-three clinical controls were assessed. Clients underwent residential treatment and met clinical criteria for eating disorders (*n* = 39), depression (*n* = 24), anxiety disorders (*n* = 11), and obsessive-compulsive disorders (*n* = 19). Among the clinical control group, twenty-one clients exhibited subsyndromal symptoms of BPD, fulfilling three or four diagnostic criteria, while four clients met the criteria for a formal BPD diagnosis by fulfilling five or more criteria. These four clients were included in the BPD group for analysis. The recruitment phase for the clinical controls lasted from April 2024 to June 2024. Inclusion criteria were: (a) age between 18 and 65 years, (b) a primary diagnosis of eating disorders, OCD, anxiety disorders, or depression, and (c) not having been in treatment for longer than six weeks. Exclusion criteria included: (a) a diagnosis of BPD (≥ 5 IPDE criteria), (b) the presence of PTSD, (c) bipolar I disorder, and (d) current or lifetime psychosis. Client eligibility was determined in two steps: first, by reviewing the digital case records for the diagnoses made by the treating clinician (psychiatrist or psychotherapist), and second, by examining BPD symptoms using the IPDE [4].

The healthy control group was recruited at the Central Institute of Mental Health via advertising and social media. A telephone screening was conducted before participation to verify the inclusion criteria. Individuals with a history of somatic or mental illness or psychiatric or psychotherapeutic treatment were excluded. The recruitment period of the healthy controls was from January 2024 to February 2025. One participant was excluded from the analysis after disclosing passive suicidal ideation during the interview.

All participants provided written informed consent to participate in the study. The study and consent forms were reviewed and approved by the applicable ethics committees at each study center (2023 − 518).

### Measures

*Borderline Symptom List – Interview (BSL-I).* The BSL-I is a semi-structured interview comprising 31 items rated on a 5-point Likert scale. Table 1 presents the structure of the BSL-I, including specific items, dimensions, and the calculation of the scale score.

Twenty-five items (Items 1–25) evaluate BPD-related symptom frequency (0 = never, 4 = very often) and associated distress (0 = no distress, 4 = most severe distress). These items include both BPD-specific, prototypic symptoms (e.g., fear of abandonment) and typical, but not specific symptoms (e.g., suicidality). Of these 25 items, four items (Items 6, 9, 10, and 25) additionally assess the consequences of behavioral symptoms such as anger management, self-harm, and suicidal behavior, rated from 0 (no problematic behavior) to 4 (highly threatening behavior with severe consequences). Five reversed items (Items 26–30) measure aspects of positive mental health, assessing both frequency (0 = never, 4 = very often) and intensity (0 = no respective feeling, 4 = intensely pronounced respective feeling). One item evaluates psychosocial impairment in daily life (Item 31) related to practical skills, social contacts, and occupational/educational functioning, rated on a 5-point Likert scale (0 = no impairment, 4 = most severe impairment, daily). Overall symptom severity is calculated based on four conceptual dimensions: symptom frequency, symptom distress, behavioral consequences, and psychosocial impairment. The German and English versions of the BSL-I, along with the calculation sheets, are provided in the supplementary materials.

**Table 1:** Structure of the multidimensional borderline symptom List-Interview (BSL-I)

Item no.	Item title	Symptom frequency	Distress/ intensity (*R*)	Behavioral consequences	Impairment in daily life
	Scale Dimensions	Dimension 1 (D1)	Dimension 2 (D2)	Dimension 3 (D3)	Dimension 4 (D4)
1	Aversive Inner Tension	✔	✔		
2	Mood Swings	✔	✔		
3	Emotional Numbness	✔	✔		
4	Shame & Guilt	✔	✔		
5	Self-Loathing or Self-Hatred	✔	✔		
6	Irritability & Anger	✔	✔	✔	
7	Helplessness	✔	✔		
8	Dissociation	✔	✔		
9	Urge to Self-Harm	✔	✔	✔	
10	Suicidal Thoughts	✔	✔	✔	
11	Perceived Threat	✔	✔		
12	Loneliness	✔	✔		
13	Abandonment	✔	✔		
14	Identity: Coherence and Consistency	✔	✔		
15	Emptiness	✔	✔		
16	Judgment uncertainty	✔	✔		
17	Worthlessness	✔	✔		
18	Fear of Failure	✔	✔		
19	Negative Body-Self	✔	✔		
20	Difficulties with Trust	✔	✔		
21	Social Exclusion & Humiliation	✔	✔		
22	Alienation	✔	✔		
23	Intrusions and Flashbacks	✔	✔		
24	(Pseudo)-Hallucinations	✔	✔		
25	Behavior Control	✔	✔	✔	
26	Hope and Confidence (R)	✔	✔		
27	Meaningfulness (R)	✔	✔		
28	Life Satisfaction (R)	✔	✔		
29	Joy or Happiness (R)	✔	✔		
30	Security and Comfort (R)	✔	✔		
Overall functioning item					
31	Impairment in daily life				
	Daily practical skills				✔
	Social contacts				✔
	Profession/Training/School				✔
Dimension scores		$\:\:\varvec{D}1=\frac{{\sum\:}_{\varvec{i}=1}^{30}{\varvec{I}}_{\varvec{i}}}{30}$	$\:\varvec{D}2=\frac{{\sum\:}_{\varvec{i}=1}^{30}{\varvec{I}}_{\varvec{i}}}{30}$	$\:\varvec{D}3=\frac{{\sum\:}_{\varvec{i}=1}^{4}{\varvec{I}}_{\varvec{i}}}{4}$	$\:\varvec{D}4=\frac{{\sum\:}_{\varvec{i}=1}^{3}{\varvec{I}}_{\varvec{i}}}{3}$
Scale score		$\:\varvec{M}=\frac{{\sum\:}_{\varvec{i}=1}^{4}{\varvec{D}}_{\varvec{i}}}{4}$			

*Note* BSL-I: Items, dimensions, and scale score (mean) calculation. R = reversed items. Symptom frequency: Occurrence of symptoms. Distress: Subjective distress. Intensity (R): Intensity of positive affect. Behavioral consequences: (Potentially) dangerous consequences of behavior. Impairment in life: functional impairment. Each dimension score is the mean of the sum score of its items (Items = I). Scale score = mean of four dimension scores: Distress, Behavioral Consequences, Intensity, and Impairment in Life

*International Personality Disorder Examination* (IPDE) [4]. The IPDE is a semi-structured clinical interview designed to assess the presence of the nine diagnostic criteria for BPD according to the DSM-IV. A BPD diagnosis is established when an individual meets five or more criteria. The IPDE has demonstrated good interrater reliability in previous research, with kappa values ranging from 0.76 to 0.89 for BPD diagnoses [4]. In this study, all interviews were conducted by trained psychologists with experience in structured diagnostic assessments. The IPDE criteria were assessed in the clinical samples (BPD and CC) and were used to establish the construct validity of the BSL-I. Specifically, the convergent validity of the BSL-I was evaluated by examining its correlation with the IPDE criteria. We hypothesized a strong correlation between the BSL-I score and the IPDE criteria, as both instruments reflect underlying dimensions of BPD severity.

*Mini International Neuropsychiatric Interview* (M.I.N.I.) [37]. The M.I.N.I. is a semi-structured interview designed to assess major Axis I disorders according to the DSM-5 and ICD-10. It is divided into modules corresponding to different diagnostic categories. The diagnostic results from the M.I.N.I. were used to determine the eligibility of individuals with BPD.

*Borderline Symptom List (BSL-23)* [10]. The BSL-23 was administered to assess the severity of BPD. This instrument consists of 23 items evaluating typical BPD symptoms during the past week, rated on a 5-point Likert scale (0 = not at all, 4 = very much). The BSL-23 is widely used, has been translated into 18 languages, and demonstrated excellent internal consistency in our total sample, with a Cronbach’s alpha of 0.95 (95% CI [0.94, 0.96]). The mean score, calculated as the average of the 23 items, was assessed across all samples and used to establish the criterion validity of the BSL-I for the BPD group and the entire sample. We hypothesized a strong correlation between the BSL-I Score and the BSL-23, as the BSL-23 served as the conceptual framework for developing the BSL-I in assessing BPD severity.

To establish construct validity, additional established measures were included. Specifically, subscales conceptually related to BPD symptoms were used to assess convergent validity, while conceptually distinct constructs were used to evaluate discriminant validity.

*Symptom Checklist-27 (SCL-27)* [38]. The SCL-27 was used to screen for symptoms across a broader spectrum of psychiatric disorders. This self-rating instrument includes 27 items divided into six subscales: depressive, dysthymic, vegetative, agoraphobic, sociophobic symptoms, and symptoms of mistrust. In this study, we used the subscales of physical and emotional symptoms (lifetime and past two weeks). Internal consistency for these subscales in our total sample was Cronbach’s alpha = 0.92 (95% CI [0.91, 0.93]) for the physical symptoms subscale, 0.91 (95% CI [0.89, 0.92]) for emotional symptoms in the past two weeks, and 0.85 (95% CI [0.81, 0.87]) for the subscale of emotional symptoms across a lifetime. These values suggest good to excellent internal consistency across subscales. The subscales assessing physical and emotional symptoms (lifetime and past two weeks) were used to establish convergent validity for the BPD group and the entire sample. We expected a moderate to strong correlation between the BSL-I score and the subscale for physical symptoms. We also expected a strong correlation with the subscale of emotional symptoms related to the past two weeks, as the BSL-I evaluates both symptoms and emotional states within that timeframe. Finally, we expected a small to moderate correlation with the subscale of emotional symptoms experienced throughout a lifetime.

*Quality of Life Enjoyment and Satisfaction Questionnaire—Short Form (Q-LES-Q-SF*) [39]. The Q-LES-Q-SF was administered to assess the quality of life. This self-rating instrument consists of 16 items measuring satisfaction over the past week, rated on a 5-point scale from 1 (*Very Poor*) to 5 (*Very Good*). In our total sample, the internal consistency of the Q-LES-Q-SF was found to be excellent with Cronbach’s alpha of 0.91 (95% CI [0.89, 0.92]). The total score was used as a near-neighbor construct to establish discriminant validity for the BPD group and the entire sample. As quality of life is conceptually distinct from symptom severity, we expected a moderate to strong negative correlation with the BSL-I score, with higher BPD severity expected to relate to lower life satisfaction.

*Mehrfachwahl-Wortschatz-Intelligenztest* (MWT) [40]. The MWT was administered to estimate premorbid IQ and to characterize the sample. The MWT is a multiple-choice vocabulary intelligence test comprising 37-word lists. The internal consistency in our total sample was 0.71 (95% CI [0.66, 0.75]). The normed score from the MWT was used to assess discriminant validity for the BPD group and the entire sample. We expected that the correlation between the BSL-I Score and the MWT Score would be minimal, given that these two instruments assess different constructs, thus reinforcing the discriminant validity of the BSL-I.

The strength of the correlations in this study was interpreted according to Cohen’s [41] guidelines, which categorize correlations as small (*r* = 0.10), moderate (*r* = 0.30), and strong (*r* = 0.50).

## Statistical analysis

For our statistical analysis and data visualization, we utilized R version 2023.06.1 + 524 [42]. While all items of the BSL-I were fully completed, 3% of individual item responses were missing across the dataset due to entry errors. The missing values were imputed utilizing the group-specific mean corresponding to their respective items.

### Determination of sample size

To determine the sample size for this study, we conducted a literature review and performed a formal power analysis. We determined the sample size for BPD clients by reviewing the literature on sample size requirements for validating psychiatric scales [43]. Additionally, we used validated statistical software (SAS™ version 9.4 [44]) to perform the formal power analysis. The sample size was calculated for a two-sided test (*α* = 0.05, power = 0.90) assuming an unbalanced allocation (BPD: CC = 9:1) and a conservative between-group effect size (Cohen’s d = 0.80). Although Kleindienst et al. [45] reported a larger effect size for BPD_CAL vs. CC (d = 0.99), we opted for a more conservative estimate (d = 0.80) to avoid overestimating the effect size and to ensure that the study remains adequately powered even if the true effect is smaller than previously reported. This resulted in a total sample size of *N* = 190 (171 BPD, 19 CC). We then applied the calculated sample size to each group in the project, including the HCs.

### Sample analysis

To investigate age differences among the BPD, CC, and HC groups, we conducted a one-way ANOVA followed by Bonferroni-corrected post-hoc tests to control for Type I error inflation. We reported eta squared (η²) as the effect size measure.

Gender differences were analyzed using Pearson’s chi-squared test, along with pairwise comparisons via Fisher’s exact test, adjusting p-values with Bonferroni correction. Cramér’s V was employed as the measure of effect size.

To examine differences in education level, we conducted a Kruskal-Wallis test followed by Dunn’s post-hoc tests with the Bonferroni correction, calculating partial eta squared (η²ₚ) to assess the effect size.

We compared premorbid IQ scores (MWT) among groups through one-way ANOVA and Bonferroni-corrected post-hoc tests, reporting eta squared (η²) as the effect size.

We also examined group differences in IPDE criteria between CC and BPD participants using a Kruskal-Wallis test and calculated partial eta squared (η²ₚ).

Furthermore, we conducted one-way ANOVAs with Bonferroni corrections to assess group differences in BSL-23, SCL-27 physical symptoms, SCL-27 emotional symptoms (past two weeks), SCL-27 emotional symptoms (lifetime), and Q-LES-Q-SF scores, reporting eta squared (η²) for effect sizes.

Finally, we examined group differences in BSL-I scores via a one-way ANOVA with Bonferroni post-hoc tests and reported eta squared (η²) as the measure of effect size.

### Psychometric properties

We computed Cronbach’s alpha [46] for each item and the total scale to determine internal consistency. We calculated the Spearman correlation between the BSL-I and IPDE scores to establish criterion validity.

We calculated the Intraclass Correlation Coefficient (ICC) of the BSL-I scores between two assessors to evaluate its inter-rater reliability (IRR), utilizing a two-way model with a consistency type. Moreover, we generated a Bland-Altman plot to assess the agreement between the assessors’ ratings for each client.

Additionally, we computed Pearson correlations between the BSL-I and established instruments, including the BSL-23 and SCL-27 (with subscales) for convergent validity and the Q-LES-Q-SF and premorbid IQ (MWT) for discriminant validity.

We plotted receiver operating characteristic (ROC) curves to evaluate the discriminative ability of the BSL-I in distinguishing between individuals with and without BPD, as well as between clinical groups and healthy controls. For comparison, we plotted ROC curves for the BSL-23 using the same group contrasts. Additionally, DeLong’s tests for correlated ROC curves were conducted to assess whether the differences in AUCs between the BSL-I and BSL-23 were statistically significant across both group comparisons.

The optimal cut-off for the BSL-I was determined using the Youden Index [47] to achieve the optimal balance between true positive and true negative rates, thereby ensuring the best classification performance.

### Severity degrees

We determined the severity classification of the BSL-I score based on the distribution of the BPD sample. To do so, we assessed the normality of the BSL-I scores in the BPD group by calculating skewness and kurtosis, followed by an Anderson-Darling test to evaluate deviations from normality. The mean and standard deviation were used to establish six distribution-based severity categories: (1) Extremely high: scores greater than two standard deviations above the mean; (2) Very high: scores between one and two standard deviations above the mean; (3) High: scores between the mean and one standard deviation above the mean; (4) Moderate: scores between the mean and one standard deviation below the mean; (5) Mild: scores between one and two standard deviations below the mean; (6) None or low: scores from 0 to less than two standard deviations below the mean. We performed Spearman’s correlations to examine the relationships between BSL-I severity degrees with the BSL-I score, the BSL-23 score, the BSL-23 severity degrees, and the IPDE-BPD criteria. This analysis aimed to assess potential information loss resulting from the categorization of the BSL-I score and to evaluate the validity of the derived severity degrees.

## Results

### Sample characteristics

The demographic characteristics of the sample are in Table 2. There were significant age differences between the BPD, CC, and HC groups, *F*(2, 300) = 4.41, *p* = 0.013, η² = 0.029. Post hoc pairwise comparisons with Bonferroni correction revealed a significant age difference only between the CC and HC groups (*padj* = 0.01). No significant age differences were found between the BPD and CC groups or between the BPD and HC groups. A Pearson’s chi-squared test revealed a significant gender difference between the groups, *χ*² *(*2) = 6.87, *p* = 0.032, V = 0.15. Pairwise comparisons with Bonferroni correction showed a significant gender difference only between the BPD and CC groups (*padj* = 0.041).

There was also a significant difference in the level of education between groups, *H* (2) = 17.012, *p* < 0.001, η²ₕ = 0.05. Bonferroni-corrected post hoc comparisons showed that the BPD group had a significantly lower level of education than both the CC group (*padj* = 0.29) and the HC group (*padj* = 0.001). There was no significant difference in education between the CC and HC groups. No significant difference was observed in premorbid IQ between the groups (*p* = 0.064).

**Table 2:** Sample characteristics

Column label	BPD (*n* = 171)	CC (*n* = 89)	HC (*n* = 43)	Difference		Effect size
**Age**						
Mean (SD)	30.64 (8.75)	28.36 (12.61)	34.16 (12.51)	*F*(2, 300 = 4.41	*p* = 0.013 BPD = CC BPD = HC CC < HC (*padj* = 0.001)	η² = 0.029 small
**Sex**,** n (%)**						
Female	150 (88)	67 (75)	34 (79)	*χ*² (2) = 6.87	*p* = 0.032 BPD ≠ CC (*padj* = 0.041) BPD = HC CC = HC	V = 0.15 small
Male	21 (12)	22 (25)	9 (21)			
**Education**,** n (%)**						
No school leaving certificate	5 (2.92)	1 (1.12)	0 (0)	*H* (2) = 17.01,	*p* < 0.001 BPD < CC (*padj* = 0.029) CC = HC (0.080) BPD < HC (*padj* < 0.001)	η²ₕ = 0.05 small
Secondary school leaving certificate (Volksschulabschluss)	23 (13.45)	5 (5.61)	1 (2.32)			
Secondary school certificate (Polytechnische Oberschule)	42 (24.56)	18 (20.22)	6 (13.95)			
general or subject-specific higher education entrance qualification (Abitur)	67 (39.18)	41 (46.07)	17 (39.53)			
University degree	34 (19.88)	24 (26.96)	19 (44.18)			
**Premorbid IQ (MWT**)						
Mean (SD)	103.84 (9.42)	105.56 (11.97)	108.10 (14.10)	*F*(2, 300) = 2.75	*p* = 0.064	η² = 0.02 small
**Diagnosis**,** n (%)**						
Major Depression	157 (91.81)	69 (77.53)	x			
PTSD	54 (31.58)	x	x			
GAS	47 (27.49)	5 (5.62)	x			
Other anxiety disorders	89 (52.06)	14 (15.73)	x			
OCD	23 (13.45)	21 (23.60)	x			
Eating Disorders	38 (22.22)	35 (39.33)	x			

*Note* Demographic characteristics of the Samples. BPD = Clients with Borderline Personality Disorder, CC = Clinical Controls (Eating Disorders, Major Depression, Obsessive-compulsive Disorders, and Anxiety Disorders), HC = Healthy Controls, x = Diagnosis not included due to subgroup eligibility criteria. Bold indicates variable names.

### Between-group comparisons of BSL-I and other measures

**Fig. 1:**
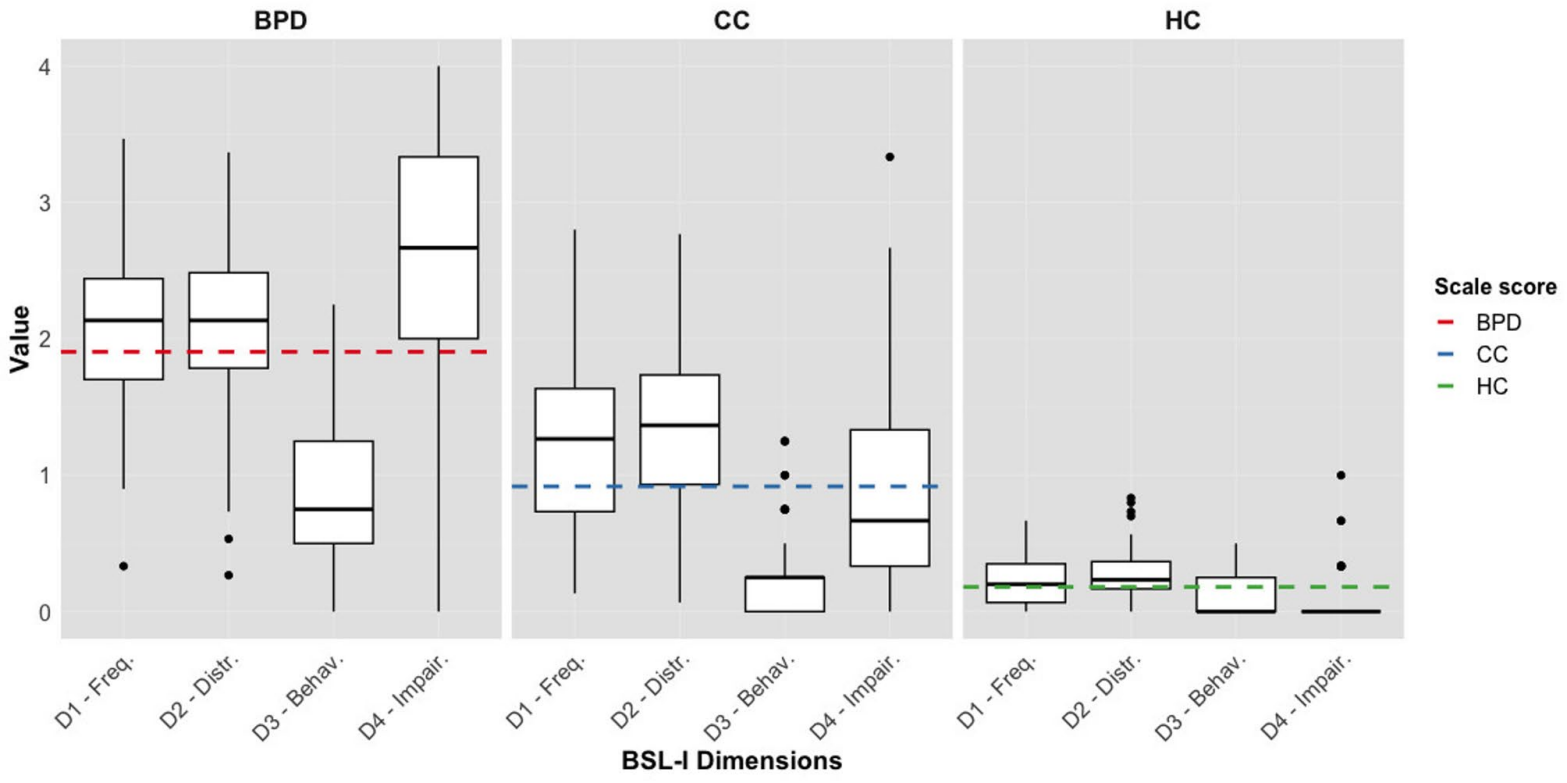
Boxplots illustrating the distribution of each diagnostic group across the four dimensions of the Borderline Symptom List – Interview (BSL-I). Dimension labels: D1 – Symptom Frequency, D2 – Subjective Distress, D3 – Behavioral Consequences, D4 – Impairment in Daily Life. Diagnostic groups include BPD (Borderline Personality Disorder), CC (Clinical Controls), and HC (Healthy Controls). The dotted line represents the scale score for each subgroup

There was a significant difference in the *BSL-I scores* between the groups, *F*(2, 301) = 605.5, *p* < 0.001, η² = 0.68. The mean score of the BPD group (*M* = 1.90, *SD* = 0.49) was significantly higher than of the CC group (*M* = 0.92, *SD* = 0.48), *p* < 0.001, *d* = 2.02, and the HC group (*M* = 0.18, *SD* = 0.15), *p* < 0.001, *d* = 3.88. The CC group scored significantly higher than the HC group, *p* < 0.001, *d* = 1.82. The distribution of the BSL-I dimensions (frequency, distress, behavioral consequences, and impairment) across each subgroup is illustrated in Fig. 1.

**Fig. 2:**
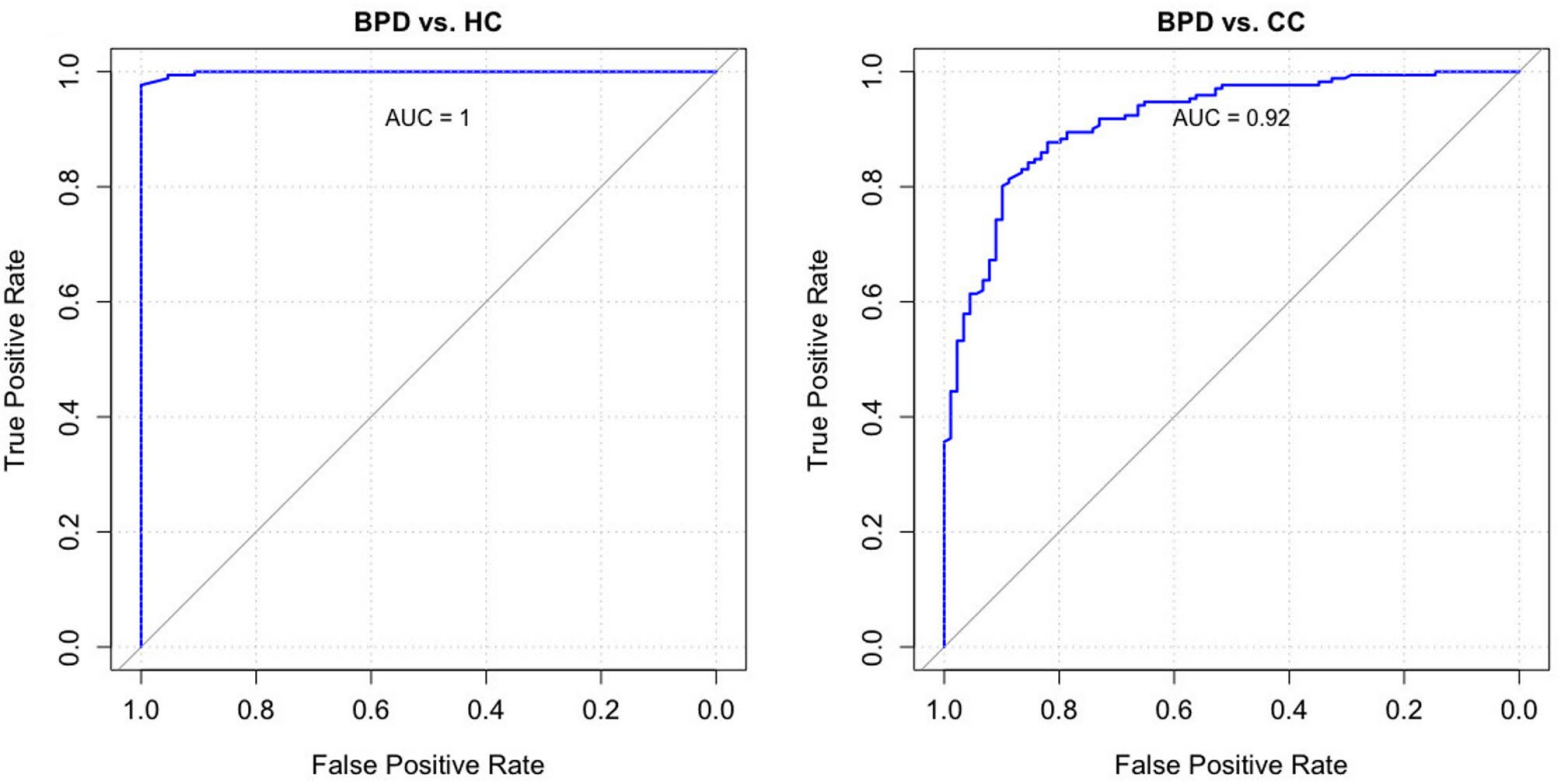
ROC curves illustrating the accuracy of the BSL-I in classifying diagnostic status (BPD vs. non-BPD) in two subsamples. Left: Borderline Personality Disorder (BPD) vs. Healthy Controls (HC); Right: Borderline Personality Disorder (BPD) vs. Clinical Controls (CC)

Group comparisons, means, and standard deviations for other investigated measures are provided in Table 3. Furthermore, the BSL-I effectively distinguished diagnostic statuses (BPD vs. non-BPD). As illustrated by the ROC curves in Fig. 2, the Area Under the Curve (AUC) for the BSL-I Mean Score in the BPD versus HC sample was extremely high at 0.99 (rounded to 1). In the BPD versus CC sample, it was also notably high at 0.92. In contrast, the BSL-23 showed a similarly high AUC of 0.99 for the BPD versus HC comparison, but a lower AUC of 0.79 for BPD versus CC (see Supplementary Fig. 1). DeLong’s test for correlated ROC curves indicated no significant difference in discriminative ability between the BSL-I and BSL-23 for the BPD versus HC comparison. However, for the BPD versus CC comparison, the BSL-I demonstrated significantly superior discriminative ability (*Z* = − 5.52, *p* < 0.001 (95% [CI: −0.16, − 0.08]. The identified optimal cut-off for the BSL-I was 1.37, resulting in a sensitivity of 0.820 and a specificity of 0.877. The complete tables of sensitivities and specificities for all values of the BSL-I are provided in supplementary Tables 1 and 2.

### Reliability

The internal consistency of the BSL-I, as measured by Cronbach’s α, was good in the BPD group (α = 0.82, 95% CI [0.76, 0.84]), based on 1,000 percentile bootstrap replicates. For the total sample, Cronbach’s α indicated high reliability (α = 0.94, 95% CI [0.93, 0.95]), also estimated using 1,000 percentile bootstrap replicates.

**Fig. 3:**
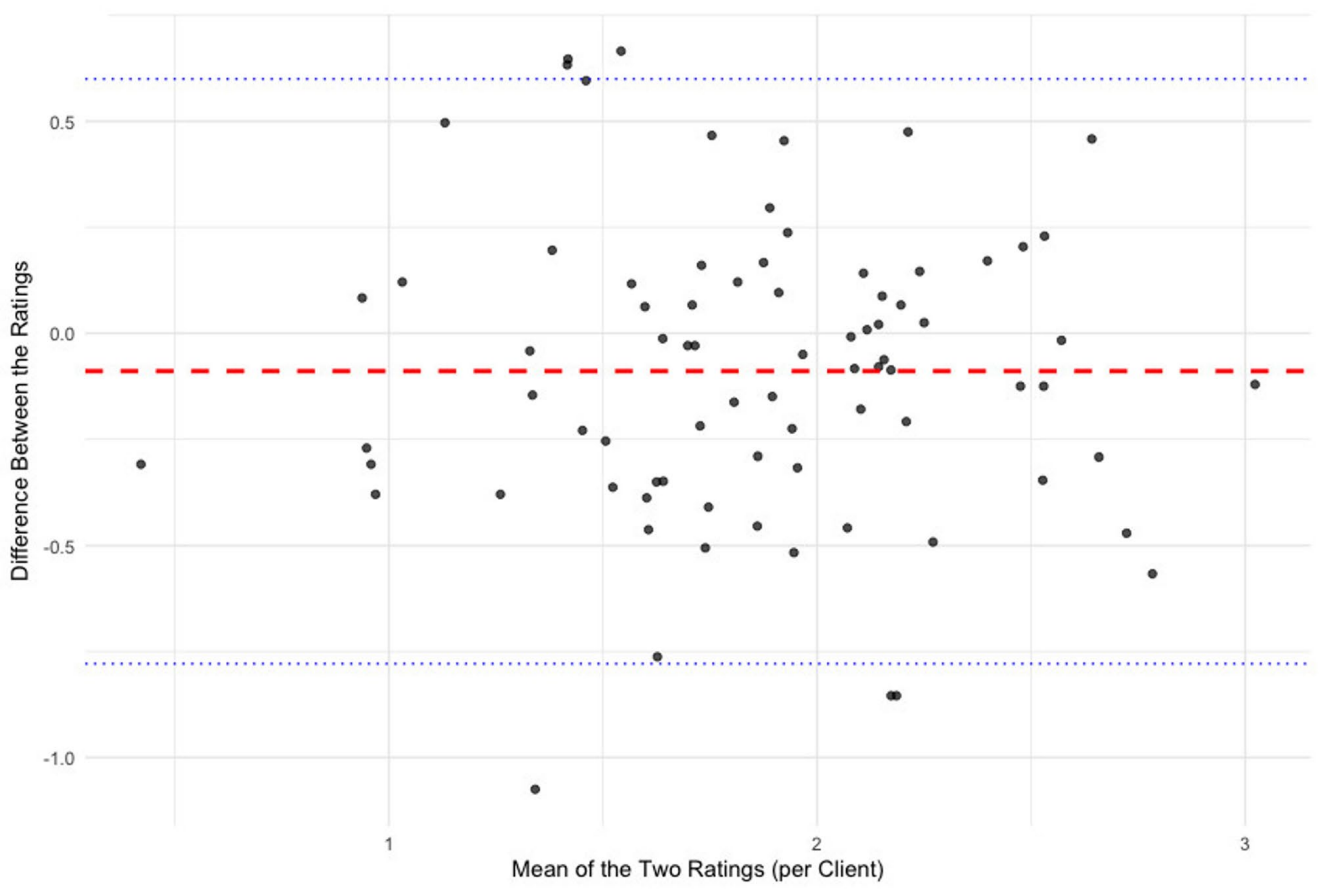
Bland-Altman plot illustrating the agreement between two raters across clients (*N* = 80) as part of the inter-rater reliability (IRR) assessment. The red dashed line represents the mean difference between the raters. The blue dotted lines indicate the 95% limits of agreement (mean ± 1.96 × SD)

Interrater reliability of the BSL-I Score was evaluated using the Intraclass Correlation Coefficient (ICC), based on the ratings from two independent assessors in a subsample of 80 BPD clients. The ICC value was 0.768 (95% CI: 0.66–0.85), demonstrating good inter-rater reliability. A significant F-test (*F*(79,79) = 7.63, *p* < 0.001, η² = 0.88) supported this conclusion, indicating that the ratings were reliable and that the variability in scores among raters was significantly higher than random error. The Bland-Altman plot for IRR (see Fig. 3) demonstrated strong consistency among the raters. Most differences remained within the 95% limits of agreement, with a mean difference near zero. There was no observable proportional bias throughout the score range.

**Table 3:** Investigated measures

Column Label	BPD (*n* = 171)	CC (*n* = 89)	HC (*n* = 43)	Difference		Effect Size
IPDE BPD criteria, M (SD)	6.23 (1.33)	1.35 (1.38)	x	*χ*² (1) = 176.74	*p* < 0.001	η²ₕ = 0.068 medium
BSL23 Score, M (SD)	1.90 (0.70)	1.08 (0.71)	0.26 (0.30)	*F*(1, 301) = 249.1	*p* < 0.001 BPD > CC (*p*adj < 0.001) CC > HC (*p*adj < 0.001)	η² = 0.045 medium
SCL-27, M (SD)						
SCL-27 Physical symptoms	3.03 (0.64)	2.60 (0.75)	1.58 (0.46)	*F*(1, 301) = 156	*p* < 0.001 BPD > CC (*p*adj < 0.001) CC > HC (*p*adj < 0.001)	η² = 0.035 medium
SCL-27 Emotional symptoms (past two weeks)	3.53 (0.81)	3.07 (1.10)	1.48 (0.61)	*F*(1, 301) = 154	*p* < 0.001 BPD > CC (*p*adj < 0.001) CC > HC (*p*adj < 0.001)	η² = 0.033 medium
SCL-27 Emotional symptoms (lifetime)	0.88 (0.21)	0.73 (0.30)	0.24 (0.28)	*F*(1, 301) = 192	*p* < 0.001 BPD > CC (*p*adj < 0.001) CC > HC (*p*adj < 0.001)	η² = 0.039 medium
Q-LES-Q-SF, M (SD)	36.2 (8.29)	42.5 (9.59)	53.6 (9.11)	*F*(1, 301) = 136	*p* < 0.001 BPD < CC (*p*adj < 0.001) CC < HC (*p*adj < 0.001)	η² = 0.031 medium

*Note* Means (M), standard deviations (SD), and group comparisons for the investigated measures between the subgroups Borderline Personality Disorder (BPD), Clinical Controls (CC), and Healthy Controls (HC)

### Convergent and discriminant validity

**Fig. 4:**
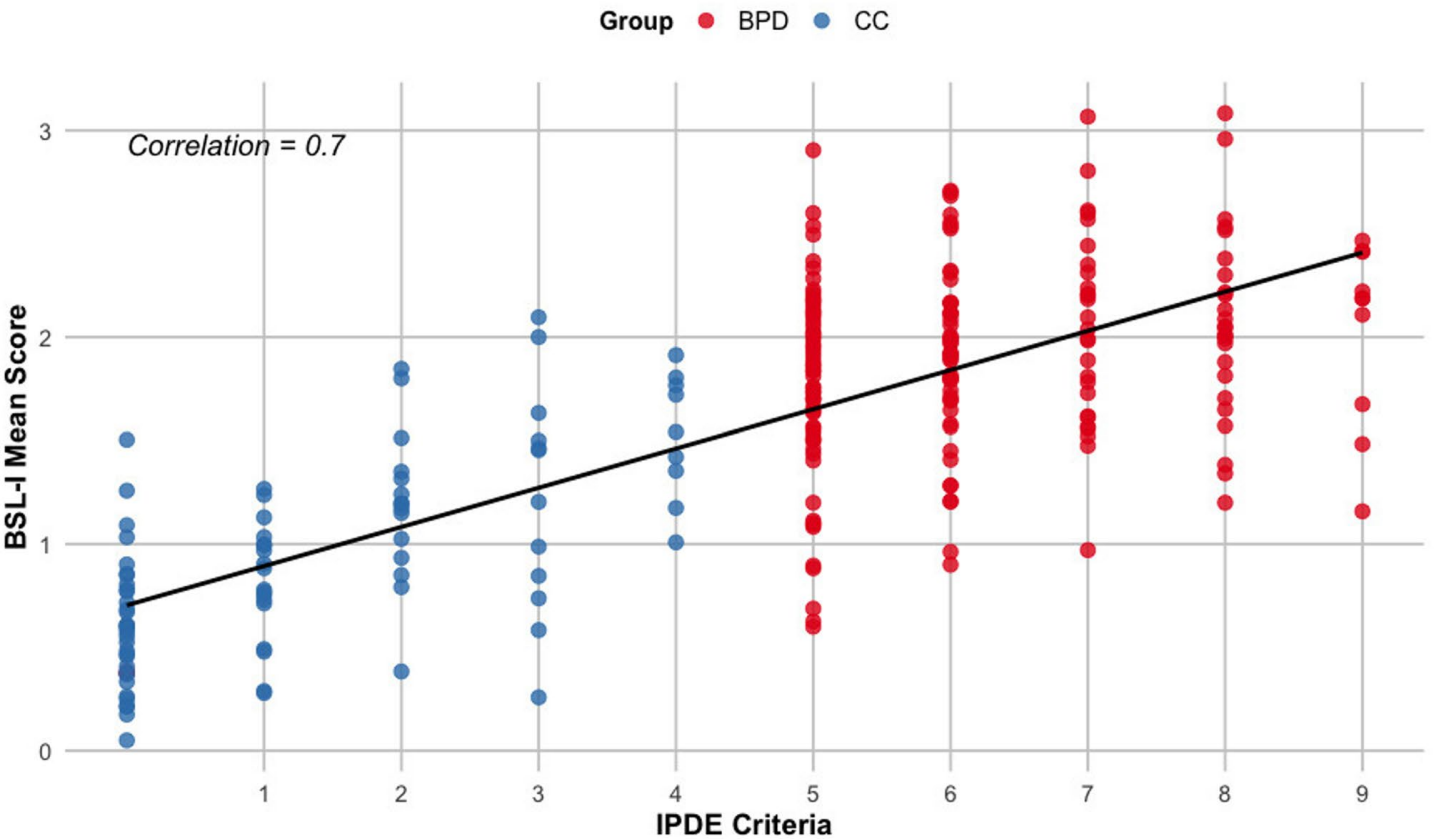
Scatterplot illustrating the correlation between IPDE-BPD criteria and BSL-I scores, assessing construct validity in a single-method comparison. BPD: Borderline Personality Disorder, CC: Clinical Controls

As expected, the BSL-I mean score strongly correlated with the number of IPDE-BPD criteria met (Spearman’s correlation) across the entire clinical sample (BPD and CC groups) (*r* = 0.70, *p* < 0.001; see Fig. 4), suggesting high convergent validity. However, in the BPD subgroup, where all individuals met at least five BPD criteria, the correlation was smaller (*r* = 0.23, *p* = 0.002), likely due to restricted variance in symptom counts.

**Fig. 5:**
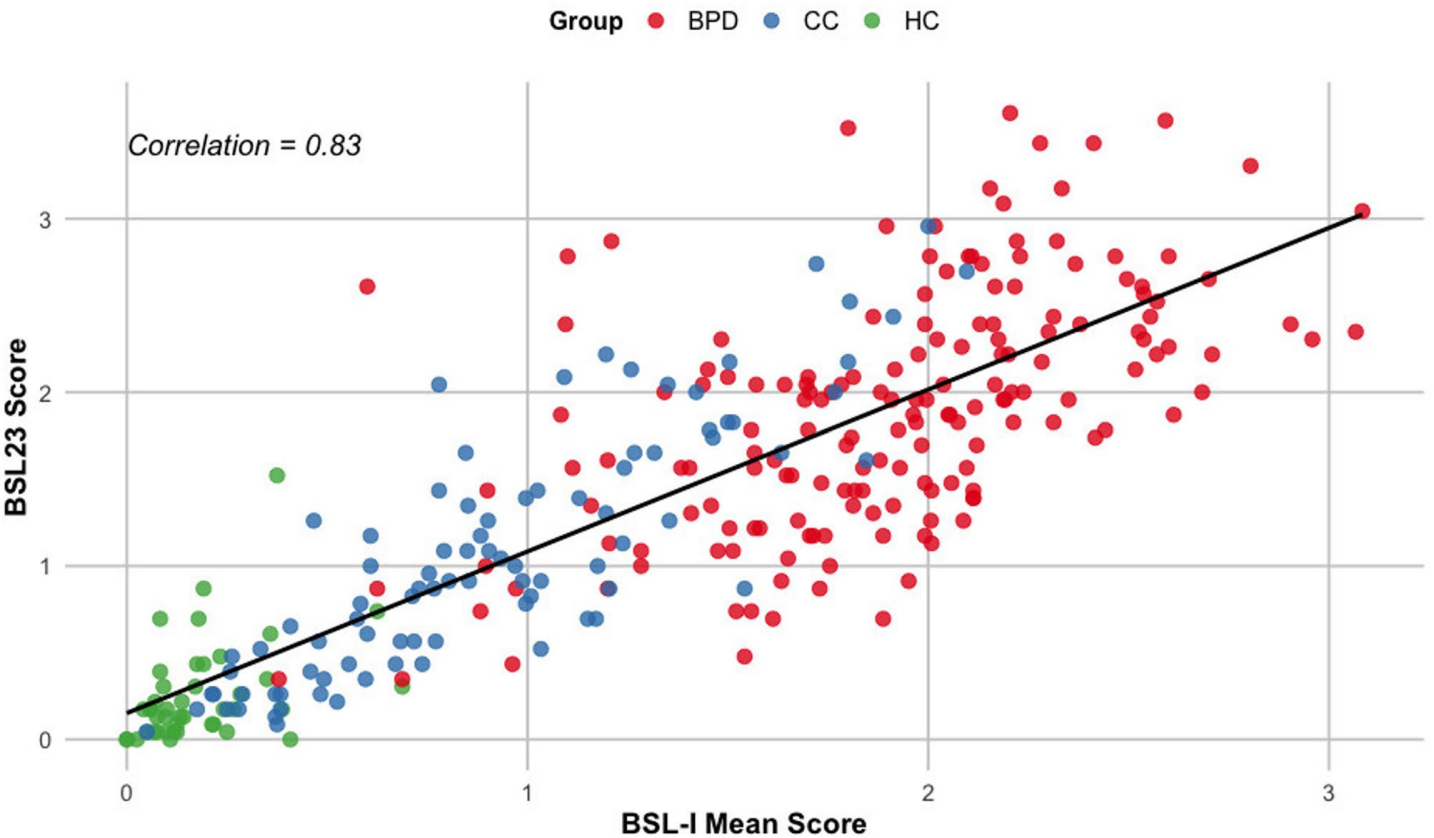
Scatterplot illustrating the correlation between BSL-23 scores (model concept) and BSL-I scores, assessing convergent validity in a multi-method comparison. BPD: Borderline Personality Disorder, CC: Clinical Controls, and HC: Healthy Controls

Furthermore, as hypothesized, the correlation between the BSL-I mean score and the BSL-23 was strong in the total sample (*r* = 0.83, *p* < 0.001; see Fig. 5) and remained strong in the BPD group (*r* = 0.57, *p* < 0.001), indicating high criterion validity.

The correlation between the BSL-I mean score and the SCL-27 physical symptom subscale was strong for the total sample (*r* = 0.69, *p* < 0.001) and moderate in the BPD group (*r* = 0.45, *p* < 0.001), thus supporting the hypothesis of a moderate to strong correlation and convergent validity. Similarly, the correlation between the BSL-I mean score and the SCL-27 emotional symptom subscale for the past two weeks was strong in the total sample (*r* = 0.67, *p* < 0.001) and moderate in the BPD group (*r* = 0.40, *p* < 0.001), consistent with the expected strong correlation. The correlation between the BSL-I mean score and the SCL-27 emotional symptoms (lifetime) was strong for the total sample (*r* = 0.55, *p* < 0.001), exceeding the hypothesized small to moderate correlation. However, in the BPD group, the correlation between the BSL-I mean score and the SCL-27 emotional symptoms (lifetime) was negligible (*r* = 0.05, *p* = 0.661), contrary to the expectation of a small correlation.

As expected, the correlation between the BSL-I and the Q-LES-Q-SF was strongly negative for the total sample (*r* = −0.68, *p* < 0.001) and moderately negative for the BPD sample (*r* = −0.48, *p* = 0.001), supporting the hypothesis and providing evidence for discriminant validity.

The correlation between the BSL-I and the MWT-IQ was small for the total sample (*r* = −0.14, *p* < 0.001) and similarly small for the BPD sample (*r* = −0.03, *p* = 0.609), which is consistent with the hypothesis and provides evidence for the discriminant validity of the BSL-I.

**Fig. 6:**
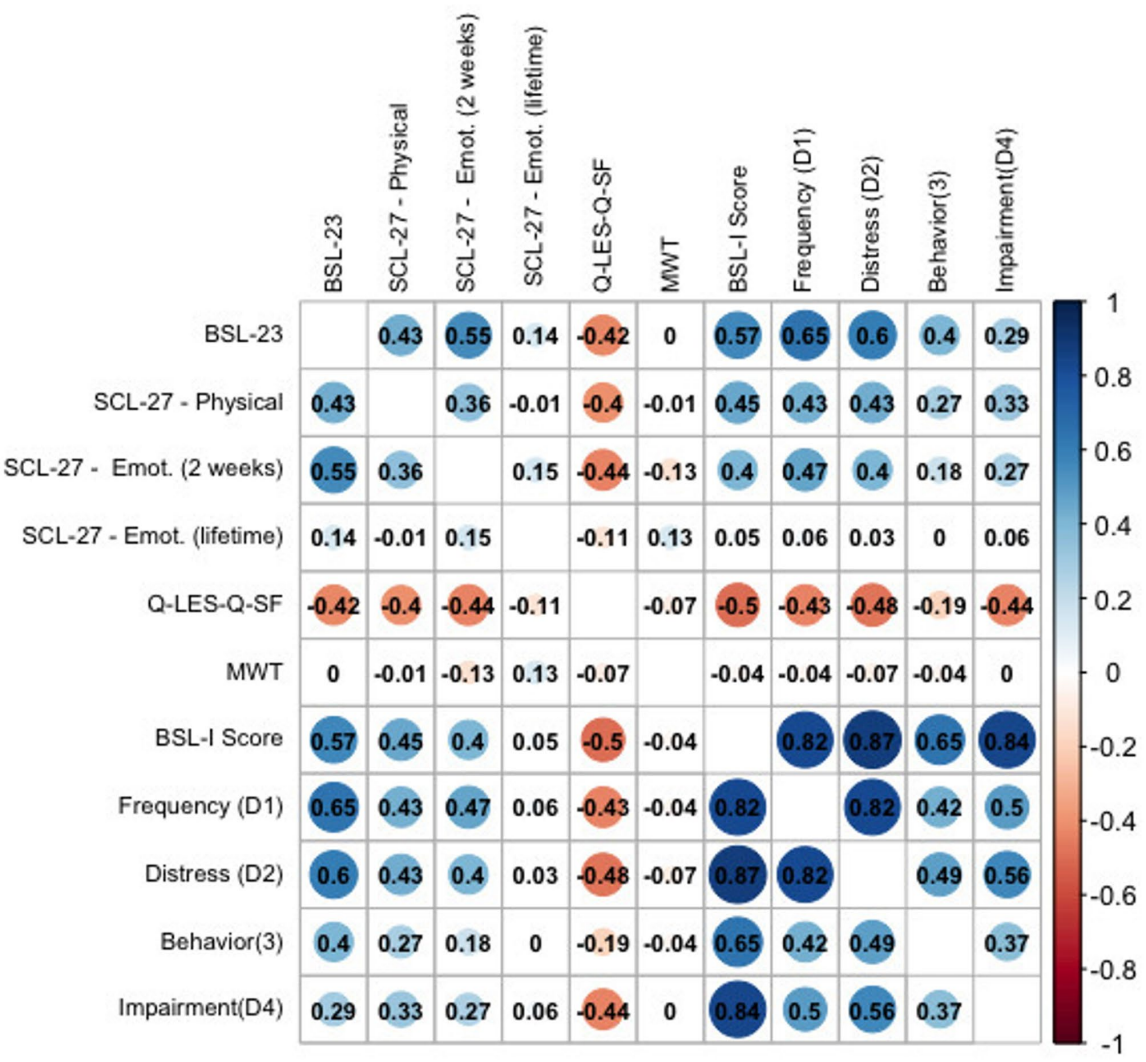
Pearson correlations between the BSL-I score and its dimensions with established measures for convergent and discriminant validity in the BPD sample. Abbreviations: BSL-23 = Borderline Symptom List-23; SCL-27 Physical = Physical Symptoms Subscale; SCL-27 Emotional (2 weeks) = Emotional Symptoms Subscale (past 2 weeks); SCL-27 Emotional (lifetime) = Emotional Symptoms Subscale (lifetime); Q-LES-Q-SF = Quality of Life; MWT = premorbid IQ; BSL-I = Borderline Symptom List – Interview (Total Score); BSL-I Dimensions = D1) Frequency, D2) Distress, D3) Behavioral Consequences, D4) Impairment in Daily Life

**Fig. 7:**
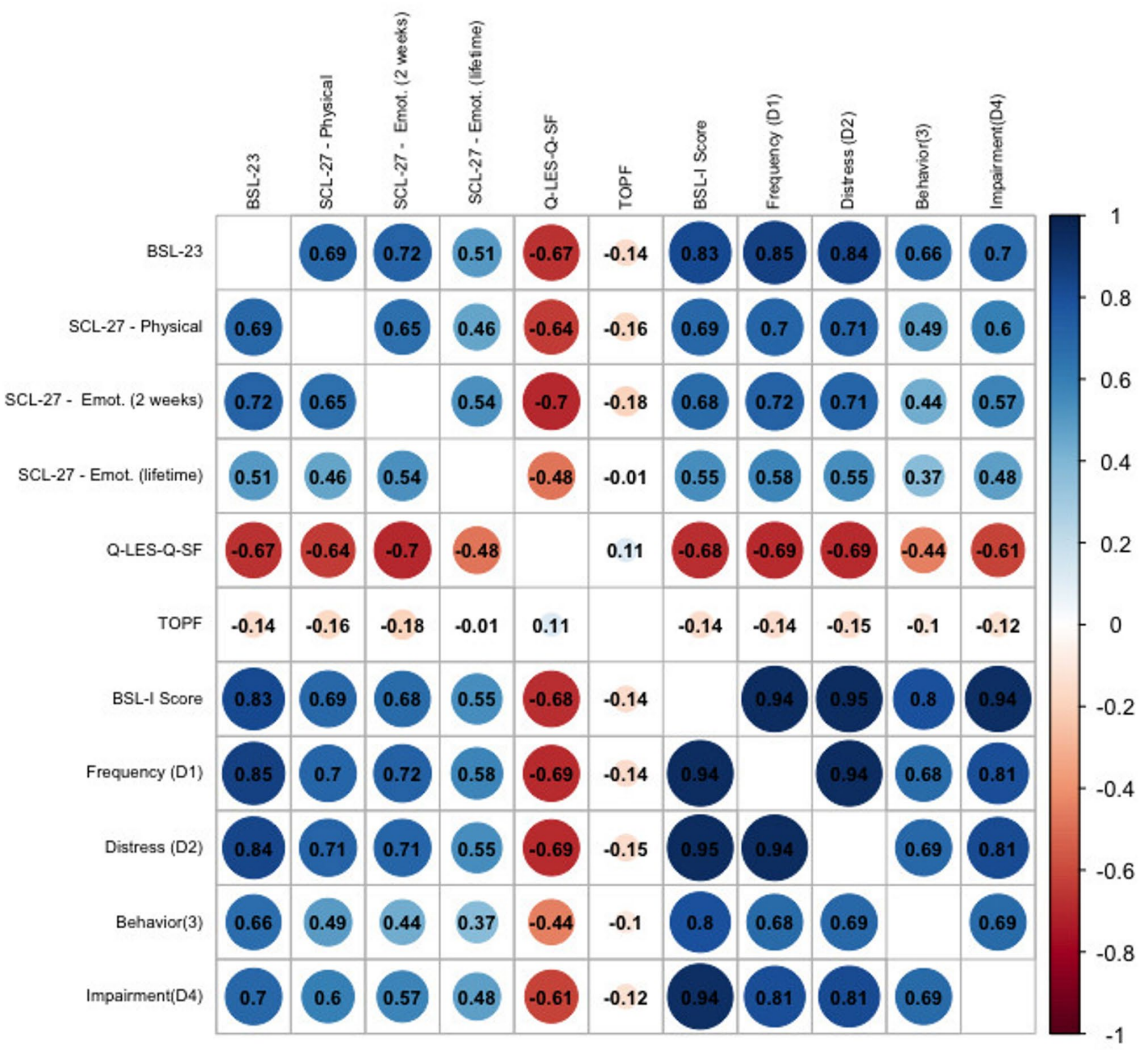
Pearson correlations between the BSL-I score and its dimensions with established convergent and discriminant validity measures in the total sample. Abbreviations: BSL-23 = Borderline Symptom List-23; SCL-27 Physical = Physical Symptoms Subscale; SCL-27 Emotional (2 weeks) = Emotional Symptoms Subscale (past 2 weeks); SCL-27 Emotional (lifetime) = Emotional Symptoms Subscale (lifetime); Q-LES-Q-SF = Quality of Life; MWT = premorbid IQ; BSL-I = Borderline Symptom List – Interview (Total Score); BSL-I Dimensions = D1) Frequency, D2) Distress, D3) Behavioral Consequences, D4) Impairment in Daily Life

Figures 6 and 7 present the complete set of correlations for both the BPD group and the total sample.

### Calculation of BSL-I severity degrees

**Fig. 8:**
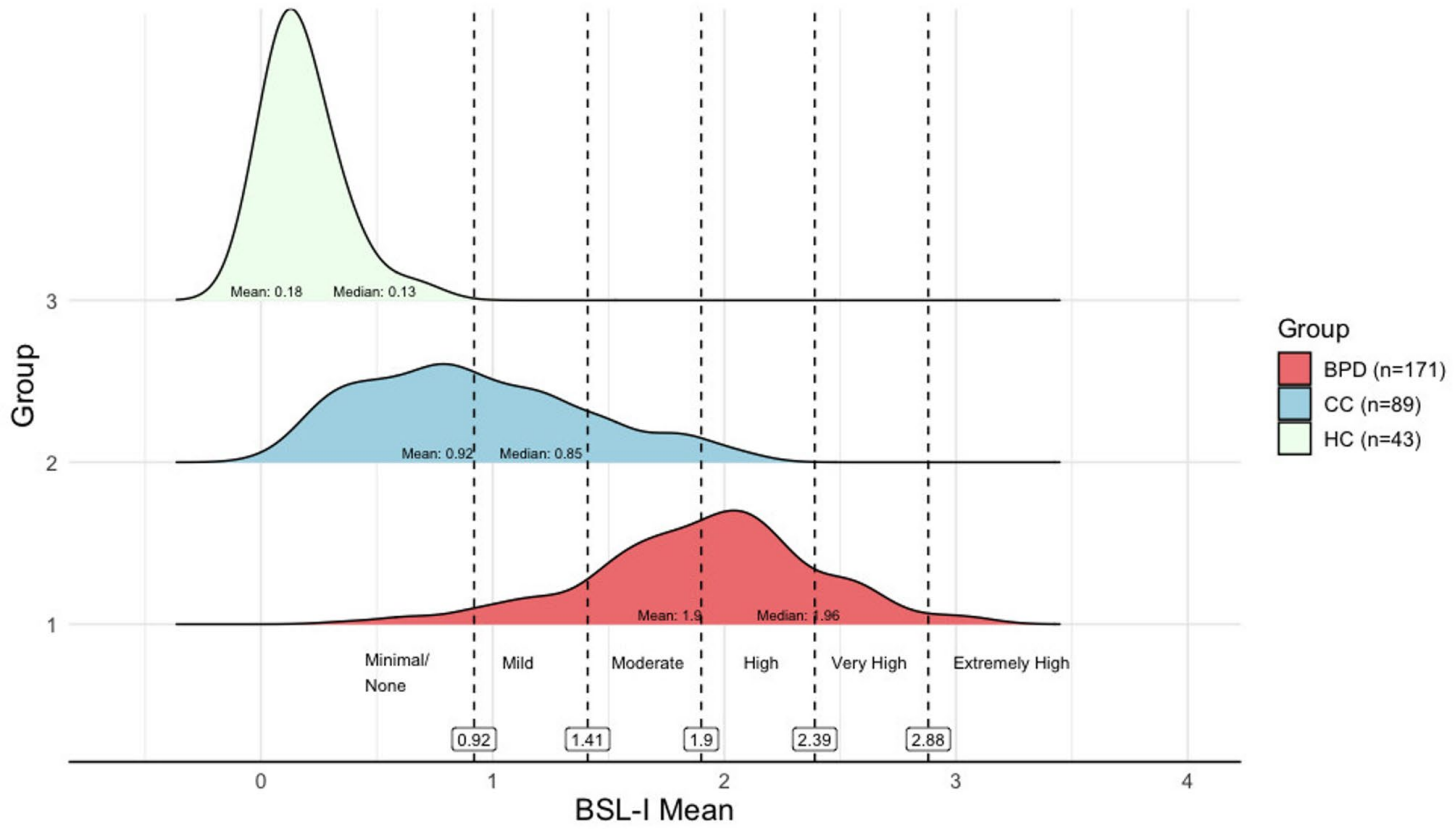
Density plot of BSL-I scores across the groups and the six severity degrees. Groups include Borderline Personality Disorder (BPD), Clinical Controls (CC), and Healthy Controls (HC)

The BSL-I scores in the BPD group aligned with the assumption of normality (Anderson-Darling *A* = 0.66, *p* = 0.08), with a skewness of −0.38 and a kurtosis of 3.38. The BSL-I mean scores were categorized into six degrees of severity based on the normal distribution of the BPD sample, using standard deviations to define the severity degrees. The theoretical minimum and maximum scores were 0 and 4, respectively. Scores between 0 and less than 0.92 fall into the none/minimal range (more than two standard deviations below the mean), scores from 0.92 to less than 1.41 indicate *mild* severity (between one and two standard deviations below the mean), scores from 1.41 to less than 1.90 indicate *moderate* severity (within one standard deviation below the mean). Scores from 1.90 to less than 2.39 fall into the *high* range (within one standard deviation above the mean). Scores from 2.39 to less than 2.88 indicate *very high* severity (one to two standard deviations above the mean). Values from 2.88 fall into the *extremely high* range (more than two standard deviations above the mean). The distribution of the BSL-I scores across groups and the severity degrees are presented in Fig. 8.

Across the entire sample of BPD, CC, and HC groups (*N* = 303), a very strong correlation was found between BSL-I scores and severity degrees (*ρ* = 0.97, *p* < 0.001). Similarly, severity degrees correlated strongly with BSL-23 scores across the entire sample (*ρ* = 0.79, *p* < 0.001). When focusing on the BPD group, the correlation between severity degrees and BSL-23 scores remained strong (*ρ* = 0.54, *p* < 0.001). The correlation between BSL-I severity degrees and BSL-23 severity degrees was also strong in the entire sample (*ρ* = 0.76, *p* < 0.001). The correlation between BSL-I and BSL-23 severity degrees in the BPD group was moderate (*ρ* = 0.48, *p* < 0.001). In addition, a strong correlation was found between the number of BPD criteria met in the IPDE and the severity degrees (*ρ* = 0.68, *p* < 0.001). In the BPD group, the correlation between BPD criteria and severity degrees was small (*ρ* = 0.23, *p* = 0.003).

The strong correlation between the BSL-I score and the severity degrees suggests that categorizing the mean score into degrees results in minimal information loss. The pattern of strong correlations between severity degrees and the BSL-I score, as well as other BPD-specific measures, supports the convergent validity of the BSL-I severity degrees. Likewise, cross-tabulating means and standard deviations for the number of IPDE-BPD criteria met, and BSL-23 scores across BSL-I severity degrees (see Table 4) demonstrate general agreement and convergence with classifications from established instruments.

**Table 4:** BSL-I severity degree, Means, and SD of the IPDE-BPD criteria and the BSL-23 score.

BSL-I score in the BPD sample		Values of external measures across the BSL-I severity degrees	
Severity Classification (BSL-I)	Range of BSL-I scores	Number of BPD-symptoms (IPDE)	Borderline Symptom List (BSL-23)
Observed Samples		Clinical sample (BPD, CC)	Total sample (BPD, CC, HC)
None or low	0-0.92 rounded (0-0.9)	1.07 (1.68)	0.52 (0.49)
Mild	0.92–1.41 rounded (0.9–1.4)	3.63 (2.53)	1.38 (0.58)
Moderate	1.41–1.90 rounded (1.4–1.9)	5.45 (1.68)	1.64 (0.57)
High	1.90–2.39 rounded (1.9–2.4)	6.21 (1.39)	2.16 (0.60)
Very high	2.39–2.88 rounded (2.4–2.9)	6.81 (1.29)	2.48 (0.50)
Extremely high	2.88-4 rounded (2.9-4)	7.00 (1.41)	2.52 (0.35)

*Note* BPD = Clients with Borderline Personality Disorder, CC = Clinical Controls (Eating Disorders, Major Depression, Obsessive-compulsive Disorders, and Anxiety Disorders), HC = Healthy Controls

## Discussion

This study aimed to evaluate the psychometric properties of the BSL-I, a newly developed interview designed to assess the severity of BPD symptoms. We integrated prototypic evidence-based BPD symptoms, nonspecific, but severity-indicating symptoms, as well as facets of positive mental health. We conceptualized BPD symptom severity across four dimensions: symptom frequency, subjective level of distress, behavioral consequences, and impairments in daily life. Based on the BPD sample, we established six severity degrees: (1) None or low, (2) mild, (3) moderate, (4) high, (5) very high, and (6) extremely high.

The findings suggest that the BSL-I is a reliable instrument for assessing BPD symptom severity, with psychometric properties ranging from good to excellent. The interview demonstrates strong internal consistency, indicating high item homogeneity and reliable assessment of BPD symptomatology. The good interrater reliability further supports the consistency and objectivity of the BSL-I, indicating its applicability in clinical and research settings and highlighting the clarity and feasibility of the assessment for assessors.

The BSL-I exhibits high convergent validity with the IPDE-BPD criteria [4] and strong criterion validity with the BSL-23 [10], which served as our model concept. Consequently, our operationalization of BPD symptom severity—incorporating frequency, subjective distress, behavioral consequences, and functional impairment—aligns with general BPD pathology and the BPD severity model underlying the BSL-23. Thus, the BSL-I is the corresponding semi-structured interview to the established self-rating BSL-23.

Overall, the majority of the hypotheses were confirmed, providing further evidence for the construct and convergent validity of the BSL-I. It is important to note, however, that the correlations between the BSL-I, IPDE-BPD criteria, and BSL-23 are lower when considering only the BPD group. This suggests that some covariance may be lost when focusing exclusively on individuals with a high symptom range (≥ 5 BPD criteria met). The BSL-I is, therefore, also capable of capturing psychopathological manifestations in subthreshold clients who do not meet the full diagnostic criteria for a BPD diagnosis.

Although the BSL-I assesses BPD dimensionally rather than categorically—and can therefore be implemented regardless of the DSM-5 diagnostic threshold—it accurately distinguishes between BPD and non-BPD clients, as indicated by its elevated AUC values on the ROC curves.

The BSL-I demonstrates superior discriminative ability compared to the BSL-23 [10]. Although both instruments exhibit extremely high discriminative performance in differentiating between BPD and HC groups, the BSL-I`s ability to discriminate BPD and CCs is significantly better for the BSL-I when compared to the BSL-23.

The significant mean differences between the HC, CC, and BPD groups further support its discriminative ability. While the distinction between HC and BPD is clear, some overlap remains between the CC and BPD groups (see Fig. 8). This overlap can be attributed to three main factors: (1) The CC group comprises individuals classified within the subsyndromal range for BPD, meeting 3–4 IPDE-BPD criteria (21 individuals); (2) the BSL-I captures both BPD-typical and BPD-specific symptoms. BPD-typical symptoms, such as insecurity about self-image, fear of failure, suicidality, are overarching symptoms and, therefore, also present in other disorders but remain clinically relevant to BPD. In contrast, BPD-specific symptoms, such as fear of abandonment and alienation, are predominantly observed in BPD clients. Given that the BSL-I employs a comprehensive assessment approach, these overarching symptoms, which extend beyond core BPD symptomatology, provide valuable insights into the BPD client’s broader psychological state. (3) The BSL-I includes aspects of positive mental health - such as hope, confidence, meaningfulness, and life satisfaction - which are also relevant to BPD populations but reflect general mental health aspects, making them less discriminative. 4) Diagnostic categories might be understood as heterogeneous constructs rather than discrete biological entities, influenced by both empirical findings and sociocultural contexts [48, 49].

Capturing BPD-related symptoms comprehensively—including subsyndromal manifestations and positive mental health aspects—makes BSL-I particularly valuable for treatment planning and tracking therapeutic progress.

### Key strengths and limitations

Our study has several strengths and limitations. A key strength was the incorporation of feedback from international experts and individuals with lived experience, ensuring face and content validity in alignment with the scale’s intended purpose. Another strength is the large sample size and the inclusion of clinical control groups with different primary diagnoses. This provides insight into how the BSL-I functions across subgroups and its ability to reflect disorder characteristics. Furthermore, we included symptoms beyond the DSM-5 diagnostic criteria, such as self-hate [18, 19], loneliness [50], and difficulties with trust [51] that cover the newly introduced evidence-based symptoms by the ICD-11 [6]. We also included items related to positive mental health, such as hope, confidence, meaningfulness, and life satisfaction [28, 30, 31], to capture broader emotional processing difficulties in BPD clients and acknowledge positive affect as a potential buffer to BPD severity.

Notably, the multidimensional conception of the BSL-I and the calculation of the BSL-I scale score as the mean of its four dimensions — symptom frequency, subjective level of distress, behavioral consequences, and impairments in daily life — account for the complex nature of BPD severity evaluation and represent another strength of the BSL-I. This approach automatically weights the dimensions equally, ensuring that assessors do not have to make subjective trade-offs between dimensions, for instance, between low symptom frequency paired with extremely serious behavioral consequences and high symptom frequency with minimal behavioral consequences. Likewise, the inclusion of subjective distress and functional impairments ensures that cases are also captured in which individuals experience symptoms in a more internalized manner — a pattern more common among female individuals with BPD and not easily observable through behavioral consequences, which is more typical for male individuals with BPD [52] Moreover, symptoms may remain unnoticed due to experiential avoidance [53], manifesting instead through impairments in daily functioning — for instance, avoiding grocery shopping, social situations, or frequently taking sick leave from work. Consequently, a low reported symptom frequency and distress may initially create the misleading impression that the client is doing well, even though dysfunctional coping strategies may significantly impair overall functioning, as captured by the conceptualization of the BSL-I that considers impairment in daily functioning.

One limitation is that the subgroups differed significantly in age, gender, and education. However, the effect sizes for the group differences were small. The HC group was older than the CC group; however, there were no significant age differences between the BPD and HC groups, or between the CC and HC groups. There was also a gender difference between the BPD group and the CC group, with a higher proportion of men in the CC group. This may reflect the gender bias in BPD-related mental health service use, where men tend to receive fewer psychotherapeutic and pharmacotherapeutic interventions and are more likely to utilize drug and alcohol rehabilitation services [52]. Additionally, individuals with BPD had lower educational levels compared to both the HC and CC groups, likely reflecting the functional impairments in education and employment often seen in BPD [2].

### Implications and future research

Future research should focus on assessing the sensitivity to change of the BSL-I, specifically its ability to capture real-time changes in a longitudinal setting. A deeper investigation of the BSL-I dimensions—such as frequency, subjective distress, behavioral consequences, and functional impairment—within the BPD group, both with and without comorbidities, would enhance our understanding of the disorder’s key features. Additionally, exploring item functioning in different samples could provide insights into categorizing BPD-typical and BPD-specific items, helping to deepen our understanding of BPD and its commonalities or differences with other disorders. Finally, validating and psychometrically evaluating the BSL-I in various languages is essential for enabling multinational use and investigating its intercultural generalizability.

## Conclusion

The BSL-I demonstrates high validity, highlighting its relevance for clinical practice and research. It provides a comprehensive, multidimensional assessment of BPD severity, focusing on symptom frequency, subjective distress, behavioral consequences, and functional impairment. Aligned with the ICD-11 [6] conceptualization of BPD, the BSL-I serves as an observer-based addition to the BSL-23. It enables a dimensional evaluation of BPD severity, independent of diagnostic thresholds, capturing both symptom severity continuously and within the subsyndromal range, while offering severity degrees to guide clinical and research applications.

With an average administration time of 45 min and good interrater reliability, the BSL-I is an efficient and practical instrument for clinicians and researchers. It facilitates reliable assessments with limited time and resource demands, ensuring a low client burden. The BSL-I will be available as an open-access instrument.

## Supplementary Information

Supplementary Material 1

Supplementary Material 2

Supplementary Material 3

Supplementary Material 4

Supplementary Material 5

Supplementary Material 6

## Data Availability

The BSL-I, available in both English and German, is included as Supplementary Files 1 and 2. The datasets used and/analyzed during the present study are available from the corresponding author upon reasonable request.

## Abbreviations

BPD: Borderline Personality Disorder
CC: Clinical Controls
HC: Healthy Controls
BSL-I: Borderline Symptom List – Interview
BSL-23: Borderline Symptom List – 23 items
SCL-23: Symptom Checklist-23
DDS: Demographic Data Scale
IPDE: International Personality Disorder Examination
ICC: Intraclass Correlation Coefficient
AUC: Area Under the Curve
GAS: Global Assessment Scale
OCD: Obsessive-Compulsive Disorder
PTSD: Post-Traumatic Stress Disorder
MWT: Mehrfach-Wortschatz-Test
