# Supplementary material for: The Borderline Symptom List–Interview: development and psychometric evaluation of an observer-based instrument for assessing symptom severity in borderline personality disorder
Source: Borderline Personal Disord Emot Dysregul. 2025 Aug 28;12:33. doi: 10.1186/s40479-025-00310-6 (PMC12395751; doi:10.1186/s40479-025-00310-6)
Supplement: Supplementary file 1 — Supplementary Material 1 [file 40479_2025_310_MOESM1_ESM.docx]

**Borderline Symptom List - Interview**

**BSL-I**

**English Version**

**Instruction**

The BSL-I is a semi-structured interview designed to assess the severity of borderline personality disorder symptoms experienced during the two-week period preceding the interview.

The BSL-I provides a systematic assessment of symptom frequency, the degree of subjective distress, behaviors related to potential or actual consequences, and daily functional impairment. It comprises 31 items, the majority of which address the following aspects:

1. frequency of symptom occurrence

2. intensity of distress that accompanies the symptom

3. behavioral consequences (Note: this aspect is assessed in only four items)

One additional item assesses the client’s overall functional impairment (e.g., daily activities, social relationships, and work or education). The degree to which impairment arises from the avoidance of symptoms should also be assessed.

**Note:**To ensure the proper and valid administration of the interview, thorough training of interviewers is required.

**1) Aversive inner tension and intensely uncomfortable emotions**

**Ask verbatim:** "During the last two weeks, did you experience any moments, episodes, or prolonged periods of time where you felt aversive inner tension or intensely uncomfortable emotions that you could not control?"

**If not present:** Check "0" for frequency and move to the next item.

**If present, ask for the following information:**

| **1.1 Frequency** | | | | |
| --- | --- | --- | --- | --- |
| **Prompts for more specific details:** "How often did you experience aversive inner tension or intensely uncomfortable emotions?" | | | | |
| **Notes:** | | | | |
| **Interviewer rating of frequency:** | | | | |
| 0  Never | 1  Rarely  (1-2 times in the last two weeks) | 2  Occasionally  (3-6 times in the last two weeks) | 3  Frequently  (approx. 7-8 times in the last two weeks) | 4  Very often  (Almost daily, or consistently for the last two weeks) |
| **1.2 Distress** | | | | |
| **Prompts for more specific details:** "Did you find your aversive inner tension or intensely uncomfortable emotion distressing? How severe was your distress? " | | | | |
| **Notes:** | | | | |
| **Interviewer rating of level of distress:** | | | | |
| 0  No Distress | 1  Mild Distress | 2  Moderate Distress | 3  Marked Distress | 4  Most Severe Distress |

**2) Mood swings**

**Ask verbatim:** "During the last two weeks, were there any moments, episodes, or prolonged periods of time where you experienced mood swings? For example, rapid changes in your emotions or inner feelings? "

**If not present:** Check "0" for frequency and move to the next item.

**If present, ask for the following information:**

| **2.1 Frequency** | | | | |
| --- | --- | --- | --- | --- |
| **Prompts for more specific details:** "How often did you find your mood or emotions rapidly changing? Did this happen only occasionally, or did it occur more consistently?" | | | | |
| **Notes:** | | | | |
| **Interviewer rating of frequency:** | | | | |
| 0  Never | 1  Rarely  (1-2 times in the last two weeks) | 2  Occasionally  (3-6 times in the last two weeks). | 3  Frequently  (approx. 7 -8 times in the last two weeks) | 4  Very often  (Almost daily, or consistently for the last two weeks). |
| **2.2 Distress** | | | | |
| **Prompts for more specific details: “**During the past two weeks, did you find your mood swings distressing? How severe was your distress?" | | | | |
| **Notes:** | | | | |
| **Interviewer rating of level of distress:** | | | | |
| 0  No Distress | 1  Mild Distress | 2  Moderate Distress | 3  Marked Distress | 4  Most Severe Distress |

**3) Emotional Numbness**

**Ask verbatim: "**During the last two weeks, did you experience any moments, episodes, or prolonged periods of time where you felt as if you were cut off from your feelings? For example, where you felt numb to your feelings."

**If not present:** Tick "0" for frequency and go to the next item**.**

**If present, ask for the following information:**

| **3.1. Frequency** | | | | |
| --- | --- | --- | --- | --- |
| **Prompts for more specific details:** "During the past two weeks, how often did you experience feeling emotionally numb or cut off from your emotions?" | | | | |
| **Notes:** | | | | |
| **Interviewer rating of frequency:** | | | | |
| 0  Never | 1  Rarely  (1-2 times in the last two weeks) | 2  Occasionally  (3-6 times in the last two weeks). | 3  Frequently  (approx. 7 -8 times in the last two weeks) | 4  Very often  (Almost daily, or consistently for the last two weeks). |
| **3.2 Distress** | | | | |
| **Prompts for more specific details:** "During the past two weeks, was this experience of emotional numbness distressing to you? To what extent did you feel distressed?" | | | | |
| **Notes:** | | | | |
| **Interviewer rating of level of distress:** | | | | |
| 0  No Distress | 1  Mild Distress | 2  Moderate Distress | 3  Marked Distress | 4  Most Severe Distress |

**4) Shame, Guilt**

**Ask verbatim:** "During the last two weeks, did you experience any moments, episodes, or prolonged periods of time where you felt ashamed or guilty, or thought you were a bad or evil person – even though there was no clear or appropriate reason, or the situation didn’t seem to justify those feelings?”

**If not present:** Tick "0" for frequency and go to the next item**.**

**If present, ask for the following information:**

| **4.1. Frequency** | | | | |
| --- | --- | --- | --- | --- |
| **Prompts for more specific details:** "How often have you experienced feelings of shame, guilt, or the belief that you are a bad or evil person?" | | | | |
| **Notes:** | | | | |
| **Interviewer rating of frequency:** | | | | |
| 0  Never | 1  Rarely  (1-2 times in the last two weeks) | 2  Occasionally  (3-6 times in the last two weeks). | 3  Frequently  (approx. 7 -8 times in the last two weeks) | 4  Very often  (Almost daily, or consistently for the last two weeks). |
| **4.2 Distress** | | | | |
| **Prompts for more specific details:** "Were these feelings distressing to you? To what extent did you feel distressed by them?” | | | | |
| **Notes:** | | | | |
| **Interviewer rating of level of distress:** | | | | |
| 0  No Distress | 1  Mild Distress | 2  Moderate Distress | 3  Marked Distress | 4  Most Severe Distress |

**5) Self-loathing or self-hatred**

**Ask verbatim:** "During the last two weeks, did you experience any moments, episodes, or prolonged periods of time where you saw yourself in a very negative light or hated yourself?"

**If not present:** Tick "0" for frequency and go to the next item.

**If present, ask further:** "Was this self-loathing so strong that you despised or hated yourself? Was it so strong that you wanted to just erase yourself from the earth?"

| **5.1 Frequency** | | | | |
| --- | --- | --- | --- | --- |
| **Prompts for more specific details: "**How often did you feel this way?" | | | | |
| **Notes:** | | | | |
| **Interviewer rating of frequency:** | | | | |
| 0  Never | 1  Rarely  (1-2 times in the last two weeks) | 2  Occasionally  (3-6 times in the last two weeks). | 3  Frequently  (approx. 7 -8 times in the last two weeks) | 4  Very often  (Almost daily, or consistently for the last two weeks). |
| **5.2 Distress** | | | | |
| **Prompts for more specific details:** "Were these feelings distressing to you? To what extent did you feel distressed by them?” | | | | |
| **Notes:** | | | | |
| **Interviewer rating of level of distress:** | | | | |
| 0  No Distress | 1  Mild Distress | 2  Moderate Distress | 3  Marked Distress | 4  Most Severe Distress |

**6) Irritability, anger, and aggression**

**Ask verbatim:** "During the last two weeks, did you experience any moments, episodes, or prolonged periods of time where you felt highly irritable or angry?"

**If not present:** Tick "0" for frequency and go to the next item.

**If present, ask further:** "For example, did you get angry easily? Did you have an anger outburst at someone? Did you verbally or physically assault anyone? Did you throw or break objects?"

| **6.1 Frequency** | | | | |
| --- | --- | --- | --- | --- |
| **Prompts for more specific details: "**How often did you experience irritability, anger, or aggression?" | | | | |
| **Notes:** | | | | |
| **Interviewer rating of frequency:** | | | | |
| 0  Never | 1  Rarely  (1-2 times in the last two weeks) | 2  Occasionally  (3-6 times in the last two weeks). | 3  Frequently  (approx. 7 -8 times in the last two weeks) | 4  Very often  (Almost daily, or consistently for the last two weeks). |
| **6.2 Distress** | | | | |
| **Prompts for more specific details: "**Was your irritability, anger or aggressive behavior distressing to you or others? To what extent was it distressing? **"** | | | | |
| **Notes:** | | | | |
| **Interviewer rating of level of distress:** | | | | |
| 0  No Distress | 1  Mild Distress | 2  Moderate Distress | 3  Marked Distress | 4  Most Severe Distress |
| **6.3 Behavior** | | | | |
| **Prompts for more specific details**: "What were the consequences of your irritability, anger, or aggressive outbursts?” | | | | |
| **Notes:** | | | | |
| **Interviewer rating of severity of behavior:** | | | | |
| 0  No behavior specifically targeted | 1  Inoffensive or harmless behavior with minor consequences (e.g., distraction, breaking off conversations, skills) | 2  Behavior  Concerning  behavior with noticeable negative consequences  (e.g., insulting or offending others) | 3  Threatening behavior with serious consequences  (e.g., violent quarrel, destruction of furniture) | 4  Highly threatening behavior with severe consequences (e.g., physically assaulting others or severe self-injury) |

**7) Helplessness and powerlessness**

**Ask verbatim:** "During the last two weeks, did you experience any moments, episodes, or prolonged periods of time where you felt helpless or powerless?"

**If not present:** Tick "0" for frequency and go to the next item.

**If present, ask further**, "Were these feelings so strong that you felt completely incapable of living?"

| **7.1 Frequency** | | | | |
| --- | --- | --- | --- | --- |
| **Prompts for more specific details: "**How often did you experience these feelings?" | | | | |
| **Notes:** | | | | |
| **Interviewer rating of frequency:** | | | | |
| 0  Never | 1  Rarely  (1-2 times in the last two weeks) | 2  Occasionally  (3-6 times in the last two weeks). | 3  Frequently  (approx. 7 -8 times in the last two weeks) | 4  Very often  (Almost daily, or consistently for the last two weeks). |
| **7.2 Distress** | | | | |
| **Prompts for more specific details:** "Were these feelings of helplessness and powerlessness distressing to you? To what extent? | | | | |
| **Notes:** | | | | |
| **Interviewer rating of level of distress:** | | | | |
| 0  No Distress | 1  Mild Distress | 2  Moderate Distress | 3  Marked Distress | 4  Most Severe Distress |

**8) Dissociation: Derealization, Depersonalization, Memory Lapses**

**Ask verbatim:** "During the last two weeks, did you experience any moments, episodes, or prolonged periods of time where you felt dissociated? That is, where the world seemed unreal to you or like you were detached from yourself and you couldn’t control it?"

**If not present:** Tick “0” for frequency and go to the next item.

**If present, ask further:** "Was there any change in your vision or perception of your body? Were you still able to feel pain? Were you still able to move? Were you able to speak? Were you able to remember the relevant events or were there gaps in your memory? If so, did you put yourself in danger or hurt yourself during these gaps in your memory?"

| **8.1 Frequency** | | | | |
| --- | --- | --- | --- | --- |
| **Prompts for more specific details:** "How often did you feel dissociative?" | | | | |
| **Notes:** | | | | |
| **Interviewer rating of frequency:** | | | | |
| 0  Never | 1  Rarely  (1-2 times in the last two weeks) | 2  Occasionally  (3-6 times in the last two weeks). | 3  Frequently  (approx. 7 -8 times in the last two weeks) | 4  Very often  (Almost daily, or consistently for the last two weeks). |
| **8.2 Distress** | | | | |
| **Prompts for more specific details:** "Did you find these dissociative experiences distressing? To what extent? | | | | |
| **Notes:** | | | | |
| **Interviewer rating of distress:** | | | | |
| 0  No Distress | 1  Mild Distress | 2  Moderate Distress | 3  Marked Distress | 4  Most Severe Distress |

**9) Urge to Self-Harm**

**Ask verbatim:** "During the last two weeks, did you experience any moments, episodes, or prolonged periods of time where you felt a strong urge to hurt yourself (e.g., cut yourself, burn yourself, bang your head against the wall, draw blood, etc.)?"

**If not present:** Tick "0" for frequency and go to the next item.

**If present, ask further**: "Was the urge so strong that you had difficulties controlling your behavior?"

| **9.1 Frequency** | | | | | | |
| --- | --- | --- | --- | --- | --- | --- |
| **Prompts for more specific details: "**How often did you experience the urge to harm yourself?” | | | | | | |
| **Notes:** | | | | | | |
| **Interviewer rating of frequency:** | | | | | | |
| 0  Never | 1  Rarely  (1-2 times in the last two weeks) | | 2  Occasionally  (3-6 times in the last two weeks). | 3  Frequently  (approx. 7 -8 times in the last two weeks) | 4  Very often  (Almost daily, or consistently for the last two weeks). | |
| **9.2 Distress** | | | | | | |
| **Prompts for more specific details:** To what extent did the urge for self-harm, or the self-harm itself if it occurred, became distressing for you? How intensely did you experience this distress?" | | | | | | |
| **Notes:** | | | | | | |
| **Interviewer rating of level of distress:** | | | | | | |
| 0  No Distress | | | 1  Mild Distress | 2  Moderate Distress | 3  Marked Distress | 4  Most Severe Distress |
| **9.3 Behavior (if self-injury is present)** | | | | | | |
| **Prompts for more specific details:** "What did you do to stop or reduce the intensity of your urges to self-harm? Did you harm yourself? What did you do?" | | | | | | |
| **Notes:** | | | | | | |
| **Interviewer rating of severity of behavior:** | | | | | | |
| 0  No self-harm | | 1  Inoffensive or harmless behavior with minor consequences (e.g., superficial scratching) | | 2  Concerning  behavior with noticeable negative consequences  (e.g., self-harm that required the client to tend to their wounds) | 3  Threatening behavior with serious consequences  (e.g., self-harm that required wound care by doctor) | 4  Highly threatening behavior with severe consequences (e.g., self-harm that led to massive bleeding, opening of the abdominal wall, swallowing of razor blades, etc.) |

**10) Suicidal Thoughts**

**Ask verbatim:** "During the last two weeks, did you experience any moments, episodes, or prolonged periods of time where you had suicidal thoughts?"

**If not present:** Tick "0" for frequency and go to the next item.

**If present, ask further:** "Did you have recurring suicidal thoughts without any concrete plans or recurring suicidal thoughts with concrete plans? Did you make preparations for your death, such as writing suicide notes? Did you inform people around you? Did you attempt suicide?"

| **10.1 Frequency** | | | | | | | | |
| --- | --- | --- | --- | --- | --- | --- | --- | --- |
| **Prompts for more specific details:** "How often did you have suicidal thoughts? " | | | | | | | | |
| **Notes:** | | | | | | | | |
| **Interviewer rating of frequency:** | | | | | | | | |
| 0  Never | 1  Rarely  (1-2 times in the last two weeks) | | 2  Occasionally  (3-6 times in the last two weeks). | 3  Frequently  (approx. 7 -8 times in the last two weeks) | | | | 4  Very often  (Almost daily, or consistently for the last two weeks). |
| **10.2 Distress** | | | | | | | | |
| **Prompts for more specific details:** "Were you able to control your suicidal thoughts? Were they distressing to you? To what extent?" | | | | | | | | |
| **Notes:** | | | | | | | | |
| **Interviewer rating of level of distress:** | | | | | | | | |
| 0  No Distress | | 1  Mild Distress | | | 2  Moderate Distress | | 3  Marked Distress | 4  Most Severe Distress |
| **10.3 Behavior (if suicide attempts are present).** | | | | | | | | |
| **Prompts for more specific details:** "Did you take any specific actions to stop your suicidal thoughts or reduce their intensity? Did you attempt suicide? What did you do?" | | | | | | | | |
| **Notes:** | | | | | | | | |
| **Interviewer rating of severity of behavior:** | | | | | | | | |
| 0  No suicide attempt | 1  Inoffensive or harmless behavior with minor consequences (e.g., started taking medication, then immediately stopped or sought help). | | | 2  Concerning  behavior with noticeable negative consequences  (e.g., drove to bridge with suicidal thoughts and made phone call there). | | 3  Threatening behavior with serious consequences  (e.g., potentially lethal overdose of medication, then called for help) | 4  Highly threatening or destructive behavior with severe consequences (e.g., Life-threatening attempt, saved by chance). | |

**11) Perceived Threat**

**Ask verbatim:** "During the last two weeks, were there any moments, episodes, or prolonged periods of time when you felt an unspecific sense of threat or had a persistent feeling that something bad was going to happen to you?"

**If not present:** Tick "0" for frequency and move to the next item (If it is a realistic, real threat, please tick 0).

**If present, ask further:** "Did you feel like there was a threat to you without knowing exactly where that feeling was coming from? Were there any moments you found yourself on high alert? Was there anything (e.g., specific people or events) that made you feel threatened or in danger?'"

| **11.1 Frequency** | | | | |
| --- | --- | --- | --- | --- |
| **Prompts for more specific details: "**How often did you have the unspecific feeling of threat?" | | | | |
| **Notes:** | | | | |
| **Interviewer rating of frequency:** | | | | |
| 0  Never | 1  Rarely  (1-2 times in the last two weeks) | 2  Occasionally  (3-6 times in the last two weeks). | 3  Frequently  (approx. 7 -8 times in the last two weeks) | 4  Very often  (Almost daily, or consistently for the last two weeks). |
| **11.2 Distress** | | | | |
| **Prompts for more specific details:** "Was this feeling of unspecific threat distressing to you? To what extent?" | | | | |
| **Notes:** | | | | |
| **Interviewer rating of severity of distress:** | | | | |
| 0  No Distress | 1  Mild Distress | 2  Moderate Distress | 3  Marked Distress | 4  Most Severe Distress |

**12) Loneliness**

**Ask verbatim:** "During the last two weeks, were there moments, episodes, or prolonged periods of time where you felt lonely? Or where you felt completely alone in the world?"

**If not present:** Tick "0" for frequency and go to the next item.

**If present, ask further: "**Did you feel this way when you were around people? Were you able to be alone or did this increase feelings of loneliness? What did your loneliness feel like?**"**

| **12.1 Frequency** | | | | |
| --- | --- | --- | --- | --- |
| **Prompts for more specific details: "**How often did you feel lonely?" | | | | |
| **Notes:** | | | | |
| **Interviewer rating of frequency:** | | | | |
| 0  Never | 1  Rarely  (1-2 times in the last two weeks) | 2  Occasionally  (3-6 times in the last two weeks). | 3  Frequently  (approx. 7 -8 times in the last two weeks) | 4  Very often  (Almost daily, or consistently for the last two weeks). |
| **12.2 Distress** | | | | |
| **Prompts for more specific details:** "Did you feel distressed by your feelings of loneliness? To what extent?" | | | | |
| **Notes:** | | | | |
| **Interviewer rating of level of distress:** | | | | |
| 0  No Distress | 1  Mild Distress | 2  Moderate Distress | 3  Marked Distress | 4  Most Severe Distress |

**13) Fear of Abandonment**

**Ask verbatim:** "During the last two weeks, did you experience any moments, episodes, or prolonged periods of time where you felt anxious or worried about someone close to you leaving you? Or about being left alone?"

**If not present:** Tick "0" for frequency and go to the next item.

**If present, ask further:** "Could you describe those moments in more detail? Were they experiences involving other people, animals, a higher power, or perhaps simply moments in nature or with yourself?"

| **13.1 Frequency** | | | | |
| --- | --- | --- | --- | --- |
| **Prompts for more specific details:** "How often did you feel anxious or worried about someone close to you leaving you?" | | | | |
| **Notes:** | | | | |
| **Interviewer rating of frequency:** | | | | |
| 0  Never | 1  Rarely  (1-2 times in the last two weeks) | 2  Occasionally  (3-6 times in the last two weeks). | 3  Frequently  (approx. 7 -8 times in the last two weeks) | 4  Very often  (Almost daily, or consistently for the last two weeks). |
| **13.2 Distress** | | | | |
| **Prompts for more specific details:**  "To what extent did these fears and worries cause you distress? How intensely did you experience this distress?" | | | | |
| **Notes:** | | | | |
| **Interviewer rating of level of distress:** | | | | |
| 0  No Distress | 1  Mild Distress | 2  Moderate Distress | 3  Marked Distress | 4  Most Severe Distress |

**14) Identity: Coherence and Consistency**

**Ask verbatim:** "During the last two weeks, were there moments, episodes, or prolonged periods of time where you felt like you didn’t know who you really were, or what is important and unimportant to you? Or times when your values, goals, or preferences shifted suddenly? "

**If not present:** Tick "0" for frequency and go to the next item.

**If present, ask further**: "During these moments, did you find yourself longing for clarity about who you are? Did you feel like you had control over changes in your values, goals, or preferences?"

| **14.1 Frequency** | | | | |
| --- | --- | --- | --- | --- |
| **Prompts for more specific details:** "How often did you feel uncertain about who you are or unclear about what you want?" | | | | |
| **Notes:** | | | | |
| **Interviewer rating of frequency:** | | | | |
| 0  Never | 1  Rarely  (1-2 times in the last two weeks) | 2  Occasionally  (3-6 times in the last two weeks). | 3  Frequently  (approx. 7 -8 times in the last two weeks) | 4  Very often  (Almost daily, or consistently for the last two weeks). |
| **14.2 Distress** | | | | |
| **Prompts for more specific details:** "Did these feelings of uncertainty or longing for inner security cause you distress? To what extent?" | | | | |
| **Notes:** | | | | |
| **Interviewer rating of distress:** | | | | |
| 0  No Distress | 1  Mild Distress | 2  Moderate Distress | 3  Marked Distress | 4  Most Severe Distress |

**15) Emptiness**

**Ask verbatim:** "During the last two weeks, were there moments, episodes, or prolonged periods of time where you felt empty? Some describe this feeling as a void or a black hole. If so, can you provide more details about what this experience was like?"

**If not present:** Tick "0" for frequency and move to the next item.

**If present, ask for the following information:**

| **15.1 Frequency** | | | | |
| --- | --- | --- | --- | --- |
| **Prompts for more specific details: "**How often did you feel empty?” | | | | |
| **Notes:** | | | | |
| **Interviewer rating of frequency:** | | | | |
| 0  Never | 1  Rarely  (1-2 times in the last two weeks) | 2  Occasionally  (3-6 times in the last two weeks). | 3  Frequently  (approx. 7-8 times in the last two weeks) | 4  Very often  (Almost daily, or consistently for the last two weeks). |
| **15.2 Distress** | | | | |
| **Prompts for more specific details:** "Did you find this empty feeling distressing? To what extent?" | | | | |
| **Notes:** | | | | |
| **Interviewer rating of distress:** | | | | |
| 0  No Distress | 1  Mild Distress | 2  Moderate Distress | 3  Marked Distress | 4  Most Severe Distress |

**16) Uncertainty about one’s judgement**

**Ask verbatim:** "During the last two weeks, were there moments, episodes, or prolonged periods where you doubted your judgment of situations, events, or people because you did not trust your perception or evaluation?”

**If not present:** Tick "0" for frequency and move to the next item.

**If present, ask for the following information:**

| **16.1 Frequency** | | | | |
| --- | --- | --- | --- | --- |
| **Prompts for more specific details:** "During the last two weeks, how often did you doubt yourself?" | | | | |
| **Notes:** | | | | |
| **Interviewer rating of frequency:** | | | | |
| 0  Never | 1  Rarely  (1-2 times in the last two weeks) | 2  Occasionally  (3-6 times in the last two weeks). | 3  Frequently  (approx. 7 -8 times in the last two weeks) | 4  Very often  (Almost daily, or consistently for the last two weeks). |
| **16.2 Distress** | | | | |
| **Prompts for more specific details: "**Did these doubts distress you? To what extent?**"** | | | | |
| **Notes:** | | | | |
| **Interviewer rating of level of distress:** | | | | |
| 0  No Distress | 1  Mild Distress | 2  Moderate Distress | 3  Marked Distress | 4  Most Severe Distress |

**17) Worthlessness**

**Ask verbatim**: "During the last two weeks, how often did you experience this feeling of worthlessness or of not deserving to live? Like the world would be a better place without you?"

**If not present:** Tick "0" for frequency and move to the next item.

**If present, ask for the following information:**

| **17.1 Frequency** | | | | |
| --- | --- | --- | --- | --- |
| **Prompts for more specific details:** "During the last two weeks, how often did you experience this feeling of worthlessness?" | | | | |
| **Notes:** | | | | |
| **Interviewer rating of the severity of frequency:** | | | | |
| 0  Never | 1  Rarely  (1-2 times in the last two weeks) | 2  Occasionally  (3-6 times in the last two weeks). | 3  Frequently  (approx. 7 -8 times in the last two weeks) | 4  Very often  (Almost daily, or consistently for the last two weeks). |
| **17.2 Distress** | | | | |
| **Prompts for more specific details:** "Were you distressed by this feeling of worthlessness? To what extent?” | | | | |
| **Notes:** | | | | |
| **Interviewer rating of the severity of level of distress:** | | | | |
| 0  No Distress | 1  Mild Distress | 2  Moderate Distress | 3  Marked Distress | 4  Most Severe Distress |

**18) Lack of Self-Confidence, Self-Efficacy, Fear of Failure.**

**Ask verbatim: "**During the last two weeks, have there been any moments, episodes, or prolonged periods of time when you found it challenging to accomplish even the simplest of tasks? Or, where you experienced an emotional collapse after being criticized for making a mistake?"

**If not present:** Tick "0" for frequency and move to the next item.

**If present, ask for the following information:**

| **18.1 Frequency** | | | | |
| --- | --- | --- | --- | --- |
| **Prompts for more specific details: "**During the last two weeks, how often did you experience feeling like a failure?" | | | | |
| **Notes:** | | | | |
| **Interviewer rating of the frequency:** | | | | |
| 0  Never | 1  Rarely  (1-2 times in the last two weeks) | 2  Occasionally  (3-6 times in the last two weeks). | 3  Frequently  (approx. 7 -8 times in the last two weeks) | 4  Very often  (Almost daily, or consistently for the last two weeks). |
| **18.2 Distress** | | | | |
| **Prompts for more specific details:** "Did you find this feeling of being a failure distressing? To what extent?” | | | | |
| **Notes:** | | | | |
| **Interviewer rating of the level of distress:** | | | | |
| 0  No Distress | 1  Mild Distress | 2  Moderate Distress | 3  Marked Distress | 4  Most Severe Distress |

**19) Negative Body-Self**

**Ask verbatim:** "During the last two weeks, did you have any moments, episodes, or prolonged periods of time where you felt your body was utterly strange or disgusting?"

**If not present:** Tick "0" for frequency and go to the next item.

**If present,** continue asking, "Did you feel like you hated your body? Did you have the urge to destroy your body?"

| **19.1 Frequency** | | | | |
| --- | --- | --- | --- | --- |
| **Prompts for more specific details:** "During the last two weeks, how often have you had moments or episodes during which you hated your body or found it strange or disgusting?" | | | | |
| **Notes:** | | | | |
| **Interviewer rating of frequency:** | | | | |
| 0  Never | 1  Rarely  (1-2 times in the last two weeks) | 2  Occasionally  (3-6 times in the last two weeks). | 3  Frequently  (approx. 7 -8 times in the last two weeks) | 4  Very often  (Almost daily, or consistently for the last two weeks). |
| **19.2 Distress** | | | | |
| **Prompts for more specific details: "**Did you find this negative feeling towards your body distressing? To what extent?" | | | | |
| **Notes:** | | | | |
| **Interviewer rating of the severity of behavior:** | | | | |
| 0  No Distress | 1  Mild Distress | 2  Moderate Distress | 3  Marked Distress | 4  Most Severe Distress |

**20) Difficulties with Trust, Prosocial Signals, and Emotional Closeness**

**Ask verbatim:** "During the last two weeks, were there moments, episodes, or prolonged periods of time where it felt difficult to trust other people? Did you find it hard to be open to friendly gestures or signals from people around you? Did you find emotional closeness difficult to handle?"

**If not present:** Tick "0" for frequency and move to the next item.

**If present, ask for the following information:**

| **20.1 Frequency** | | | | | | | | |
| --- | --- | --- | --- | --- | --- | --- | --- | --- |
| **Prompts for more specific details: "**During the past two weeks, how often have you had moments or episodes of difficulty trusting others, accepting friendly signals, or allowing emotional closeness?" | | | | | | | | |
| **Notes:** | | | | | | | | |
| **Interviewer rating of the severity of frequency:** | | | | | | | | |
| 0  Never | 1  Rarely  (1-2 times in the last two weeks) | | 2  Occasionally  (3-6 times in the last two weeks). | | 3  Frequent  (approx. 7 -8 times in the last two weeks) | | 4  Very often  (Almost daily, or consistently for the last two weeks). | |
| **20.2 Distress** | | | | | | | | |
| **Prompts for more specific details:** "Did you find these difficulties trusting others distressing? To what extent?" | | | | | | | | |
| **Notes:** | | | | | | | | |
| **Interviewer rating of level of distress:** | | | | | | | | |
| 0  No Distress | | 1  Mild Distress | | 2  Moderate Distress | | 3  Marked Distress | | 4  Most Severe Distress |

**21) Social exclusion, Insult, Humiliation**

**Ask verbatim:** "During the last two weeks, have you experienced moments, episodes, or prolonged periods of time where you felt offended or excluded by others? Or, when you felt humiliated by other people?"

**If not present:** Tick "0" for frequency and go to the next item.

**If present, continue by asking,** "Can you describe these moments? How long did these feelings of social exclusion, or humiliation last?"

| **21.1 Frequency** | | | | |
| --- | --- | --- | --- | --- |
| **Prompts for more specific details:** "How often did you experience these feelings?" | | | | |
| **Notes:** | | | | |
| **Interviewer rating of the frequency:** | | | | |
| 0  Never | 1  Rarely  (1-2 times in the last two weeks) | 2  Occasionally  (3-6 times in the last two weeks). | 3  Frequently  (7 -8 times in the last two weeks) | 4  Very often  (Almost daily, or consistently for the last two weeks). |
| **21.2 Distress** | | | | |
| **Prompts for more specific details:** "Were these experiences distressing for you? To what extent?” | | | | |
| **Notes:** | | | | |
| **Interviewer rating of the level of distress:** | | | | |
| 0  No Distress | 1  Mild Distress | 2  Moderate Distress | 3  Marked Distress | 4  Most Severe Distress |

**22) Alienation**

**Ask verbatim:** "During the last two weeks, did you have any moments, episodes, or prolonged periods of time where you felt different from everyone else? That is, where you were thinking different, feeling different, or simply felt like you did not belong? Or where you sensed a gap between you and other people, or felt like you like you could not connect to others? Did you experience a sense of “homesickness” for connection with others?"

**If not present:** Tick "0" for frequency and move to the next item.

**If present, ask for the following information:**

| **22.1 Frequency** | | | | |
| --- | --- | --- | --- | --- |
| **Prompts for more specific details: "**How often did you experience this feeling of alienation?” | | | | |
| **Notes:** | | | | |
| **Interviewer rating of frequency:** | | | | |
| 0  Never | 1  Rarely  (1-2 times in the last two weeks) | 2  Occasionally  (3-6 times in the last two weeks). | 3  Frequent  (approx. 7 -8 times in the last two weeks) | 4  Very often  (Almost daily, or consistently for the last two weeks). |
| **22.2 Distress** | | | | |
| **Prompts for more specific details:** "Was this feeling of alienation distressing for you? To what extent?" | | | | |
| **Notes:** | | | | |
| **Interviewer rating of level of distress:** | | | | |
| 0  No Distress | 1  Mild Distress | 2  Moderate Distress | 3  Marked Distress | 4  Most Severe Distress |

**23) Intrusions and Flashbacks**

**Ask verbatim:** "During the last two weeks, were there any moments, episodes or prolonged periods of time where past traumatic or invalidating experiences intruded into your memory which you were not able to control? Did you experience these memories as “real” in the here and now?"

**If not present:** Tick "0" for frequency and go to the next item.

**If present, ask further:** "When you experienced these intrusions or flashbacks, did you know they were memories, or did you feel like you were re-experiencing these moments (i.e., you lost contact with reality)?"

| **23.1 Frequency** | | | | |
| --- | --- | --- | --- | --- |
| **Prompts for more specific details: "**How often did you have these intrusions or flashbacks?" | | | | |
| **Notes:** | | | | |
| **Interviewer rating of frequency:** | | | | |
| 0  Never | 1  Rarely  (1-2 times in the last two weeks) | 2  Occasionally  (3-6 times in the last two weeks). | 3  Frequently  (approx. 7 -8 times in the last two weeks) | 4  Very often  (Almost daily, or consistently for the last two weeks). |
| **23.2 Distress** | | | | |
| **Prompts for more specific details:** "Were these intrusions or flashbacks distressing to you? To what extent?” | | | | |
| **Notes:** | | | | |
| **Interviewer rating of level of distress:** | | | | |
| 0  No Distress | 1  Mild Distress | 2  Moderate Distress | 3  Marked Distress | 4  Most Severe Distress |

**24) Visual or Auditory (Pseudo)-Hallucinations**

**Ask verbatim:** "During the last two weeks, did you experience any moments, episodes, or prolonged periods of time where you heard voices (inside or outside of your head) that you know are not real? Or, where you saw things (people, creatures, or images) that did not exist?”

**If not present:** Tick "0" for frequency and go to the next item.

**If present, ask further:** "When you heard these voices, were they your own thoughts or the voices of other people? Did the voices insult or criticize you when you wanted to be nice to yourself? Did they give you instructions (e.g., tell you to do something to yourself or others)?

When you saw things that did not exist, did they seem threatening? Did a familiar person’s face, hands or voice ever suddenly change, transform, or become menacing?"

| **24.1 Frequency** | | | | |
| --- | --- | --- | --- | --- |
| **Prompts for more specific details: "**How often have you had these auditory or visual experiences?" | | | | |
| **Notes:** | | | | |
| **Interviewer rating of frequency:** | | | | |
| 0  Never | 1  Rarely  (1-2 times in the last two weeks) | 2  Occasionally  (3-6 times in the last two weeks). | 3  Frequently  (approx. 7 -8 times in the last two weeks) | 4  Very often  (Almost daily, or consistently for the last two weeks). |
| **24.2 Distress** | | | | |
| **Prompts for more specific details:** "To what extent did these voices or images distress you? Did you feel like you had to follow the voices? Did you feel like you had control over your behavior?” | | | | |
| **Notes:** | | | | |
| **Interviewer rating of level of distress:** | | | | |
| 0  No Distress | 1  Mild Distress | 2  Moderate Distress | 3  Marked Distress | 4  Most Severe Distress |

**25) Behavior Control** (Note: This item excludes anger outbursts (Item 6), self-injury (Item 9), and suicidal behavior (Item 10).)

**Ask verbatim:** "During the last two weeks, did you experience any moments, episodes, or prolonged periods of time where you had trouble controlling your behavior (not including self-harm)? Did you engage in behaviors you later regretted? For example, binge eating and/or vomiting episodes; high-risk behaviors like speeding; walking on bridge railings, railroad tracks, etc.; severe alcohol or drugs; (u/o use of non-prescribed medications); reckless sexual contact that was unsafe or associated with remorse; blood draws?"

**If not present:** Tick "0" for frequency and go to the next item.

**If present, ask further:** "If so, can you give me examples?"

| **25.1 Frequency** | | | | |
| --- | --- | --- | --- | --- |
| **Prompts for more specific details:** "How often did you have trouble controlling your problem behaviors? Please list your problem behaviors." | | | | |
| **Notes:** | | | | |
| **Interviewer rating of frequency:** | | | | |
| 0  Never | 1  Rarely  (1-2 times in the last two weeks) | 2  Occasionally  (3-6 times in the last two weeks). | 3  Frequently  (approx. 7 -8 times in the last two weeks) | 4  Very often  (Almost daily, or consistently for the last two weeks). |
| **25.2 Distress** | | | | |
| **Prompts for more specific details:** "Does your difficulty controlling your behavior cause you distress? To what extent?" | | | | |
| **Notes:** | | | | |
| **Interviewer rating of level of distress:** | | | | |
| 0  No Distress | 1  Mild Distress | 2  Moderate Distress | 3  Marked Distress | 4  Most Severe Distress |
| **25.3 Behavior** | | | | |
| **Prompts for more specific details:** "What behaviors did you engage in and what were the consequences of your behavior? If there were multiple behaviors, please identify and refer to the one with the most dangerous consequences." | | | | |
| **Notes:** | | | | |
| **Interviewer rating of the severity of behavior:** | | | | |
| 0  No problematic behavior | 1  Inoffensive or harmless behavior with minor consequences (e.g., distractions, binge eating, skills) | 2  Concerning  behavior with noticeable negative consequences (e.g., unprotected sexual intercourse with strangers). | 3  Threatening behavior with serious consequences  (e.g., speeding on the highway). | 4  Highly threatening or destructive behavior with severe consequences (e.g., balancing on high bridge railings). |

**26) Hope and confidence**

**Ask verbatim:** "During the last two weeks, did you experience any moments, episodes, or prolonged periods of time where you felt confident and hopeful that you could handle your life? Or where you felt optimistic that things will get better in the future?

**If not present:** Tick “0” for frequency and go to the next item.

**If present, ask for the following information:**

| **26.1 Frequency** | | | | |
| --- | --- | --- | --- | --- |
| **Prompts for more specific details: "**How often did you feel hopeful or confident?" | | | | |
| **Notes:** | | | | |
| **Interviewer rating of the frequency:** | | | | |
| 0  Never | 1  Rarely  (1-2 times in the last two weeks) | 2  Occasionally  (3-6 times in the last two weeks). | 3  Frequently  (approx. 7 -8 times in the last two weeks) | 4  Very often  (Almost daily, or consistently for the last two weeks). |
| **26.2 Intensity** | | | | |
| **Prompts for more specific details:** "If you felt the sense of confidence and hope - how intense or how strong were these feelings?” | | | | |
| **Notes:** | | | | |
| **Interviewer rating of the intensity:** | | | | |
| 0  No hope or confidence | 1  Fleeting Hope and confidence | 2  Clear hope and confidence | 3  Strong hope and confidence | 4  Intensely pronounced hope and confidence |

**27) Meaningfulness**

**Ask verbatim:** "During the last two weeks, did you experience any moments, episodes, or prolonged periods of time where you felt like you were living a meaningful life, or that your existence was meaningful to other people? Did you feel like you were engaged in activities that were important to you? Did you feel like you were creating a life worth living?"

**If not present:**  Tick "0" for frequency and go to the next item.

**If present, ask for the following information:**

| **27.1 Frequency** | | | | |
| --- | --- | --- | --- | --- |
| **Prompts for more specific details: "**How often have you had this sense of meaningfulness?" | | | | |
| **Notes:** | | | | |
| **Interviewer rating of the frequency:** | | | | |
| 0  Never | 1  Rarely  (1-2 times in the last two weeks) | 2  Occasionally  (3-6 times in the last two weeks). | 3  Frequently  (approx. 7 -8 times in the last two weeks) | 4  Very often  (Almost daily, or consistently for the last two weeks). |
| **27.2 Intensity** | | | | |
| **Prompts for more specific details: "**If you felt you were living a meaningful life, how intensely or how strongly did you feel this? | | | | |
| **Notes:** | | | | |
| **Interviewer rating of the intensity:** | | | | |
| 0  No meaningfulness | 1  Fleeting feeling of meaningfulness | 2  Clear sense of meaningfulness | 3  Strong sense of meaningfulness | 4  Intense feeling of meaningfulness |

**28) Life satisfaction**

**Ask verbatim:**  "During the last two weeks, did you experience any moments, episodes, or prolonged periods of time when you felt at peace with your life as it is right now, or even satisfied with your life overall? Were there any aspects that you definitely did not want to change?"

**If not present:** Tick “0” for frequency and go to the next item.

**If present, ask for the following information:**

| **28.1 Frequency** | | | | |
| --- | --- | --- | --- | --- |
| **Prompts for more specific details: "**How often did you feel satisfied with your life?" | | | | |
| **Notes:** | | | | |
| **Interviewer rating of the frequency:** | | | | |
| 0  Never | 1  Rarely  (1-2 times in the last two weeks) | 2  Occasionally  (3-6 times in the last two weeks). | 3  Frequently  (approx. 7 -8 times in the last two weeks) | 4  Very often  (Almost daily, or consistently for the last two weeks). |
| **28.2 Intensity** | | | | |
| **Prompts for more specific details: "**If you felt that you were okay with your life, or even satisfied with it as it is - how intensely or how strongly did you feel this?" | | | | |
| **Notes:** | | | | |
| **Interviewer rating of the intensity:** | | | | |
| 0  No Satisfaction | 1  Fleeting feeling of satisfaction | 2  Clear sense of satisfaction | 3  Strong feeling of satisfaction | 4  Intense feeling of satisfaction |

**29) Joy or happiness**

**Ask verbatim:** "During the last two weeks, did you experience any moments, episodes, or prolonged periods of time where you felt joy or happiness?"

**If not present:** Tick "0" for frequency and end the interview.

**If present, ask further:** "Can you describe these moments in more detail? Were they any moments of exhilaration or deeply felt happiness?"

| **29.1 Frequency** | | | | |
| --- | --- | --- | --- | --- |
| **Prompts for more specific details: "**If so, how often have you had this feeling of joy or happiness?" | | | | |
| **Notes:** | | | | |
| **Interviewer rating of the frequency:** | | | | |
| 0  Never | 1  Rarely  (1-2 times in the last two weeks) | 2  Occasionally  (3-6 times in the last two weeks). | 3  Frequently  (approx. 7 -8 times in the last two weeks) | 4  Very often  (Almost daily, or consistently for the last two weeks). |
| **29.2 Intensity** | | | | |
| **Prompts for more specific details: "**When you had these feelings of joy or happiness - how intense or how strong did you feel this?" | | | | |
| **Notes:** | | | | |
| **Interviewer rating of the intensity:** | | | | |
| 0  No Joy or happiness | 1  Fleeting feeling of joy or happiness | 2  Clear feeling of joy or happiness | 3  Strong feeling of joy or happiness | 4  Intense feeling of joy or happiness |

**30) Security and Comfort**

**Ask verbatim:** "During the last two weeks, did you experience any moments, episodes, or prolonged periods of time where you felt secure and comfortable?"

**If not present:** Tick "0" for frequency and end the interview.

**If present, ask further: "**Can you describe these moments in more detail? When did these experiences occur? Who were you with?"

| 30.1 Frequency | | | | |
| --- | --- | --- | --- | --- |
| **Prompts for more specific details:** "How often did you have this feeling of security?" | | | | |
| Notes: | | | | |
| **Interviewer rating of the frequency:** | | | | |
| 0  Never | 1  Rarely  (1-2 times in the last two weeks) | 2  Occasionally  (3-6 times in the last two weeks). | 3  Frequently  (approx. 7 -8 times in the last two weeks) | 4  Very often  (Almost daily, or consistently for the last two weeks). |
| 30.2 Intensity | | | | |
| **Prompts for more specific details:** "If you had this feeling of security - how intense or how strong did you feel this?" | | | | |
| Notes: | | | | |
| **Interviewer rating of the intensity:** | | | | |
| 0  No feeling of security | 1  Fleeting feeling of security | 2  Clear feeling of security | 3  Strong feeling of security | 4  Intense feeling of security |

**31) Impairment in daily life**

**Ask verbatim:** "Think about all of the symptoms I’ve asked you about so far: Over the past two weeks, how much and how severely did they interfere with your daily life (e.g., how much did they impact your job, school, relationships, or activities of daily living such as cooking for yourself, grocery shopping, hygiene)?"

| **31.1 Daily practical skills** | | | | |
| --- | --- | --- | --- | --- |
| **"In terms of activities of daily living**: Dis you experience any limitations in daily living activities because of your symptoms or because you feared you might experience any of these symptoms? Were you able to get up, leave the house, do your daily activities, take care of your household, children, pets, or other dependents (if you have them)?" | | | | |
| **Notes:** | | | | |
| **Interviewer rating of the severity and frequency of the impairment:** | | | | |
| 0  No impairment | 1  Mild Impairment: one-time, or a few days | 2  Moderate Impairment: a few days | 3  Marked  Impairment: predominant number of days | 4  Most Severe impairment: daily |
| **31.2 Social contacts** | | | | |
| **"In terms of your relationships (i.e., romantic partners, friends, neighbors):** Did you experience any limitations because of your symptoms or because your feared you might experience any of these symptoms? Did you have difficulties with your partner? Were you able to get together with friends or acquaintances? Did you experience any conflict?" | | | | |
| **Notes:** | | | | |
| **Interviewer rating of the severity and frequency of the impairment:** | | | | |
| 0  No impairment | 1  Mild Impairment: one-time, or a few days | 2  Moderate Impairment: a few days | 3  Marked  Impairment: predominant number of days | 4  Most Severe impairment: daily |
| **31.3 Profession / Training / School** | | | | |
| **"In terms of your occupational situation (e.g., your job, school, or other work-related activities):**  Did you experience any limitations because of your symptoms or because you feared you might experience any of these symptoms? Did you have any conflict at work or school? Were you able to complete all your work or school tasks?" | | | | |
| **Notes:** | | | | |
| **Interviewer rating of the severity and frequency of the impairment:** | | | | |
| 0  No impairment | 1  Mid Impairment: one-time, or a few days | 2  Moderate Impairment: a few days | 3  Marked  Impairment: predominant number of days | 4  Most Severe impairment: daily |
