# Supplementary material for: The Borderline Symptom List–Interview: development and psychometric evaluation of an observer-based instrument for assessing symptom severity in borderline personality disorder
Source: Borderline Personal Disord Emot Dysregul. 2025 Aug 28;12:33. doi: 10.1186/s40479-025-00310-6 (PMC12395751; doi:10.1186/s40479-025-00310-6)
Supplement: Supplementary file 2 — Supplementary Material 2 [file 40479_2025_310_MOESM2_ESM.docx]

# **Beispiel zur Kalkulation des BSL-I Scores**

| Item Nr. | Item Titel | Symptom Häufigkeit | Belastung/  Intensität (R) | Verhaltenskonsequenzen | Alltagsbeeinträchtigung |
| --- | --- | --- | --- | --- | --- |
|  |  | Dimension 1  (D1) | Dimension 2  (D2) | Dimension 3  (D3) | Dimension 4  (D4) |
| 1 | Anspannung | **2** | **3** |  |  |
| 2 | Stimmungsschwankungen | **3** | **3** |  |  |
| 3 | Emotionale Taubheit | **1** | **2** |  |  |
| 4 | Scham / Schuld | **3** | **4** |  |  |
| 5 | Selbstverachtung / Selbsthass | **2** | **2** |  |  |
| 6 | Gereiztheit / Wut / Aggression | **4** | **3** | **3** |  |
| 7 | Hilflosigkeit / Ohnmacht | **3** | **4** |  |  |
| 8 | Dissoziation | **2** | **2** |  |  |
| 9 | Selbstverletzungsdrang | **1** | **3** | **2** |  |
| 10 | Suizidgedanken | **3** | **2** | **3** |  |
| 11 | Bedrohungsgefühl | **2** | **2** |  |  |
| 12 | Einsamkeit | **3** | **3** |  |  |
| 13 | Angst vor Verlassenwerden | **1** | **4** |  |  |
| 14 | Identität: Kohärenz und Konsistenz | **2** | **3** |  |  |
| 15 | Innere Leere | **4** | **2** |  |  |
| 16 | Zweifel an der eigenen Urteilsfähigkeit | **1** | **2** |  |  |
| 17 | Wertlosigkeit | **2** | **3** |  |  |
| 18 | Versagensängste | **1** | **3** |  |  |
| 19 | Negatives Körperselbst | **3** | **3** |  |  |
| 20 | Probleme mit Vertrauen | **2** | **2** |  |  |
| 21 | Soziale Ausgrenzung und Demütigung | **0** | **4** |  |  |
| 22 | Fremdheitsgefühl | **2** | **1** |  |  |
| 23 | Intrusionen/Flashbacks | **2** | **2** |  |  |
| 24 | (Pseudo)-Halluzinationen | **1** | **1** |  |  |
| 25 | Verhaltenskontrolle | **2** | **1** | **3** |  |
| 26 | Hoffnung und Zuversicht (R) | **2** | **1** |  |  |
| 27 | Sinnerfülltheit (R) | **2** | **3** |  |  |
| 28 | Lebenszufriedenheit (R) | **1** | **2** |  |  |
| 29 | Freude oder Glück (R) | **3** | **4** |  |  |
| 30 | Geborgenheit (R) | **2** | **1** |  |  |
| Overall functioning item | |  |  |  |  |
| 31 | **Beeinträchtigung im Alltag** |  |  |  |  |
|  | Alltagspraktische Fähigkeiten |  |  |  | **3** |
|  | Soziale Kontakte |  |  |  | **4** |
|  | Beruf/Ausbildung/Schule |  |  |  | **2** |
| Dimensionswerte | | $\boldsymbol{D}\boldsymbol{1=}\frac{\sum_{\boldsymbol{i=1}}^{\boldsymbol{30}} \boldsymbol{I}_{\boldsymbol{i}}}{\boldsymbol{30}}$  $\boldsymbol{D}\boldsymbol{1=}\frac{\boldsymbol{65}}{\boldsymbol{30}}$ **= 2.17** | $\boldsymbol{D}\boldsymbol{2=}\frac{\sum_{\boldsymbol{i=1}}^{\boldsymbol{30}} \boldsymbol{I}_{\boldsymbol{i}}}{\boldsymbol{30}}$  $\boldsymbol{D}\boldsymbol{2=}\frac{\mathbf{79}}{\boldsymbol{30}}\boldsymbol{=2.63}$ | $\boldsymbol{D}\boldsymbol{3=}\frac{\sum_{\boldsymbol{i=1}}^{\boldsymbol{4}} \boldsymbol{I}_{\boldsymbol{i}}}{\boldsymbol{4}}$  $\boldsymbol{D}\boldsymbol{3=}\frac{\boldsymbol{11}}{\boldsymbol{4}}\boldsymbol{=2.75}$ | $\boldsymbol{D}\boldsymbol{4=}\frac{\sum_{\boldsymbol{i=1}}^{\boldsymbol{3}} \boldsymbol{I}_{\boldsymbol{i}}}{\boldsymbol{3}}$  $\boldsymbol{D}\boldsymbol{4=}\frac{\mathbf{9}}{\boldsymbol{3}}\boldsymbol{=3}$ |
| Skalenwert | | $\boldsymbol{M=}\frac{\sum_{\boldsymbol{i=1}}^{\boldsymbol{4}} \boldsymbol{D}_{\boldsymbol{i}}}{\boldsymbol{4}}$ **=** $\frac{\mathbf{2.17+2.63+2.75+3}}{\boldsymbol{4}}\boldsymbol{=}\frac{\boldsymbol{10.55}}{\boldsymbol{4}}\boldsymbol{=2.63}$ **(sehr hoch)** | | | |

**Auswertungsbogen für den Borderline-Symptomliste - Interview (BSL-I) Score**

| Item Nr. | Item Titel | | Symptom Häufigkeit | Belastung/  Intensität (R) | Verhaltens-  konsequenzen | Alltags-beeinträchtigung |
| --- | --- | --- | --- | --- | --- | --- |
|  | **Dimensionen der Skala** | | Dimension 1  (D1) | Dimension 2  (D2) | Dimension 3  (D3) | Dimension 4 (D4) |
| 1 | Anspannung | |  |  |  |  |
| 2 | Stimmungsschwankungen | |  |  |  |  |
| 3 | Emotionale Taubheit | |  |  |  |  |
| 4 | Scham/Schuld | |  |  |  |  |
| 5 | Selbstverachtung/Selbsthass | |  |  |  |  |
| 6 | Gereiztheit/Wut/Aggression | |  |  |  |  |
| 7 | Hilflosigkeit/Ohnmacht | |  |  |  |  |
| 8 | Dissoziation | |  |  |  |  |
| 9 | Selbstverletzungsdrang | |  |  |  |  |
| 10 | Suizidgedanken | |  |  |  |  |
| 11 | Bedrohungsgefühl | |  |  |  |  |
| 12 | Einsamkeit | |  |  |  |  |
| 13 | Angst vor Verlassenwerden | |  |  |  |  |
| 14 | Identität: Kohärenz und Konsistenz | |  |  |  |  |
| 15 | Innere Leere | |  |  |  |  |
| 16 | Zweifel an der eigenen Urteilsfähigkeit | |  |  |  |  |
| 17 | Wertlosigkeit | |  |  |  |  |
| 18 | Versagensängste | |  |  |  |  |
| 19 | Negatives Körperselbst | |  |  |  |  |
| 20 | Probleme mit Vertrauen | |  |  |  |  |
| 21 | Soziale Ausgrenzung und Demütigung | |  |  |  |  |
| 22 | Fremdheitsgefühl | |  |  |  |  |
| 23 | Intrusionen/Flashbacks | |  |  |  |  |
| 24 | (Pseudo)-Halluzinationen | |  |  |  |  |
| 25 | Verhaltenskontrolle | |  |  |  |  |
| 26 | Hoffnung und Zuversicht (R) | |  |  |  |  |
| 27 | Sinnerfülltheit (R) | |  |  |  |  |
| 28 | Lebenszufriedenheit (R) | |  |  |  |  |
| 29 | Freude oder Glück (R) | |  |  |  |  |
| 30 | Geborgenheit (R) | |  |  |  |  |
| Item zur allgemeinen Funktionsfähigkeit | | |  |  |  |  |
| 31 | Beeinträchtigung im Alltag | |  |  |  |  |
|  | Alltagspraktische Fähigkeiten | |  |  |  |  |
|  | Soziale Kontakte | |  |  |  |  |
|  | Beruf/Ausbildung/Schule | |  |  |  |  |
| Dimensionswerte | | | $\boldsymbol{D}\boldsymbol{1=}\frac{}{\boldsymbol{30}}$ **=** | $\boldsymbol{D}\boldsymbol{2=}\frac{}{\boldsymbol{30}}$ **=** | $\boldsymbol{D}\boldsymbol{3=}\frac{}{\boldsymbol{4}}$ **=** | $\boldsymbol{D}\boldsymbol{4=}\frac{}{\boldsymbol{3}}$ **=** |
| Skalenwert | | | $\boldsymbol{M=}\frac{\sum_{\boldsymbol{i=1}}^{\boldsymbol{4}} \boldsymbol{D}_{\boldsymbol{i}}}{\boldsymbol{4}}$ $\boldsymbol{=}\frac{}{\boldsymbol{4}}\boldsymbol{=}$ | | | |
| Schweregrad | 0 – 0.91  Keine Symptomatik /minimal | 0.92 – 1.40  Leicht | 1.41 – 1.89  moderat | 1.90 – 2.38  hoch | 2.39 – 2.87  sehr hoch | 2.88 - 4  extrem hoch |
