# Supplementary material for: The Borderline Symptom List–Interview: development and psychometric evaluation of an observer-based instrument for assessing symptom severity in borderline personality disorder
Source: Borderline Personal Disord Emot Dysregul. 2025 Aug 28;12:33. doi: 10.1186/s40479-025-00310-6 (PMC12395751; doi:10.1186/s40479-025-00310-6)
Supplement: Supplementary file 3 — Supplementary Material 3 [file 40479_2025_310_MOESM3_ESM.docx]

# **Example for calculation of the BSL-I Score**

| Item No. | Item Title | Symptom Frequency | Distress/  Intensity (R) | Behavioral Consequences | Impairment in daily life |
| --- | --- | --- | --- | --- | --- |
|  |  | Dimension 1  (D1) | Dimension 2  (D2) | Dimension 3  (D3) | Dimension 4 (D4) |
| 1 | Aversive Inner Tension | **2** | **3** |  |  |
| 2 | Mood Swings | **3** | **3** |  |  |
| 3 | Emotional Numbness | **1** | **2** |  |  |
| 4 | Shame & Guilt | **3** | **4** |  |  |
| 5 | Self-Loathing or Self-Hatred | **2** | **2** |  |  |
| 6 | Irritability & Anger | **4** | **3** | **3** |  |
| 7 | Helplessness | **3** | **4** |  |  |
| 8 | Dissociation | **2** | **2** |  |  |
| 9 | Urge to Self-Harm | **1** | **3** | **2** |  |
| 10 | Suicidal Thoughts | **3** | **2** | **3** |  |
| 11 | Perceived Threat | **2** | **2** |  |  |
| 12 | Loneliness | **3** | **3** |  |  |
| 13 | Abandonment | **1** | **4** |  |  |
| 14 | Identity: Coherence and Consistency | **2** | **3** |  |  |
| 15 | Emptiness | **4** | **2** |  |  |
| 16 | Judgment uncertainty | **1** | **2** |  |  |
| 17 | Worthlessness | **2** | **3** |  |  |
| 18 | Fear of Failure | **1** | **3** |  |  |
| 19 | Negative Body-Self | **3** | **3** |  |  |
| 20 | Difficulties with Trust | **2** | **2** |  |  |
| 21 | Social Exclusion & Humiliation | **0** | **4** |  |  |
| 22 | Alienation | **2** | **1** |  |  |
| 23 | Intrusions and Flashbacks | **2** | **2** |  |  |
| 24 | (Pseudo)-Hallucinations | **1** | **1** |  |  |
| 25 | Behavior Control | **2** | **1** | **3** |  |
| 26 | Hope and Confidence (R) | **2** | **1** |  |  |
| 27 | Meaningfulness (R) | **2** | **3** |  |  |
| 28 | Life Satisfaction (R) | **1** | **2** |  |  |
| 29 | Joy or Happiness (R) | **3** | **4** |  |  |
| 30 | Security and Comfort (R) | **2** | **1** |  |  |
| Overall functioning item | |  |  |  |  |
| 31 | **Impairment in daily life** |  |  |  |  |
|  | Daily practical skills |  |  |  | **3** |
|  | Social contacts |  |  |  | **4** |
|  | Profession / Training / School |  |  |  | **2** |
| Dimension scores | | $\boldsymbol{D}\boldsymbol{1=}\frac{\sum_{\boldsymbol{i=1}}^{\boldsymbol{30}} \boldsymbol{I}_{\boldsymbol{i}}}{\boldsymbol{30}}$  $\boldsymbol{D}\boldsymbol{1=}\frac{\boldsymbol{65}}{\boldsymbol{30}}$ **= 2.17** | $\boldsymbol{D}\boldsymbol{2=}\frac{\sum_{\boldsymbol{i=1}}^{\boldsymbol{30}} \boldsymbol{I}_{\boldsymbol{i}}}{\boldsymbol{30}}$  $\boldsymbol{D}\boldsymbol{2=}\frac{\mathbf{79}}{\boldsymbol{30}}\boldsymbol{=2.63}$ | $\boldsymbol{D}\boldsymbol{3=}\frac{\sum_{\boldsymbol{i=1}}^{\boldsymbol{4}} \boldsymbol{I}_{\boldsymbol{i}}}{\boldsymbol{4}}$  $\boldsymbol{D}\boldsymbol{3=}\frac{\boldsymbol{11}}{\boldsymbol{4}}\boldsymbol{=2.75}$ | $\boldsymbol{D}\boldsymbol{4=}\frac{\sum_{\boldsymbol{i=1}}^{\boldsymbol{3}} \boldsymbol{I}_{\boldsymbol{i}}}{\boldsymbol{3}}$  $\boldsymbol{D}\boldsymbol{4=}\frac{\mathbf{9}}{\boldsymbol{3}}\boldsymbol{=3}$ |
| Scale score | | $\boldsymbol{M=}\frac{\sum_{\boldsymbol{i=1}}^{\boldsymbol{4}} \boldsymbol{D}_{\boldsymbol{i}}}{\boldsymbol{4}}$ **=** $\frac{\mathbf{2.17+2.63+2.75+3}}{\boldsymbol{4}}\boldsymbol{=}\frac{\boldsymbol{10.55}}{\boldsymbol{4}}\boldsymbol{=2.63}$ **(very high)** | | | |

**Calculation Sheet for the Borderline Symptom List – Interview (BSL-I) Score**

| Item No. | Item Title | | Symptom Frequency | Distress/  Intensity (R) | Behavioral Consequences | Impairment in  daily life |
| --- | --- | --- | --- | --- | --- | --- |
|  |  | | Dimension 1  (D1) | Dimension 2  (D2) | Dimension 3  (D3) | Dimension 4 (D4) |
| 1 | Aversive Inner Tension | |  |  |  |  |
| 2 | Mood Swings | |  |  |  |  |
| 3 | Emotional Numbness | |  |  |  |  |
| 4 | Shame & Guilt | |  |  |  |  |
| 5 | Self-Loathing or Self-Hatred | |  |  |  |  |
| 6 | Irritability & Anger | |  |  |  |  |
| 7 | Helplessness | |  |  |  |  |
| 8 | Dissociation | |  |  |  |  |
| 9 | Urge to Self-Harm | |  |  |  |  |
| 10 | Suicidal Thoughts | |  |  |  |  |
| 11 | Perceived Threat | |  |  |  |  |
| 12 | Loneliness | |  |  |  |  |
| 13 | Abandonment | |  |  |  |  |
| 14 | Identity: Coherence and Consistency | |  |  |  |  |
| 15 | Emptiness | |  |  |  |  |
| 16 | Judgment uncertainty | |  |  |  |  |
| 17 | Worthlessness | |  |  |  |  |
| 18 | Fear of Failure | |  |  |  |  |
| 19 | Negative Body-Self | |  |  |  |  |
| 20 | Difficulties with Trust | |  |  |  |  |
| 21 | Social Exclusion & Humiliation | |  |  |  |  |
| 22 | Alienation | |  |  |  |  |
| 23 | Intrusions and Flashbacks | |  |  |  |  |
| 24 | (Pseudo)-Hallucinations | |  |  |  |  |
| 25 | Behavior Control | |  |  |  |  |
| 26 | Hope and Confidence (R) | |  |  |  |  |
| 27 | Meaningfulness (R) | |  |  |  |  |
| 28 | Life Satisfaction (R) | |  |  |  |  |
| 29 | Joy or Happiness (R) | |  |  |  |  |
| 30 | Security and Comfort (R) | |  |  |  |  |
| Overall functioning item | | |  |  |  |  |
| 31 | **Impairment in daily life** | |  |  |  |  |
|  | Daily practical skills | |  |  |  |  |
|  | Social contacts | |  |  |  |  |
|  | Profession / Training / School | |  |  |  |  |
| Dimension scores | | | $\boldsymbol{D}\boldsymbol{1=}\frac{}{\boldsymbol{30}}$ **=** | $\boldsymbol{D}\boldsymbol{2=}\frac{}{\boldsymbol{30}}$ **=** | $\boldsymbol{D}\boldsymbol{3=}\frac{}{\boldsymbol{4}}$ **=** | $\boldsymbol{D}\boldsymbol{4=}\frac{}{\boldsymbol{3}}$ **=** |
| Scale score | | | $\boldsymbol{M=}\frac{\sum_{\boldsymbol{i=1}}^{\boldsymbol{4}} \boldsymbol{D}_{\boldsymbol{i}}}{\boldsymbol{4}}$ $\boldsymbol{=}\frac{}{\boldsymbol{4}}\boldsymbol{=}$ | | | |
| Severity Degree | 0 – 0.91  none/minimal | 0.92 – 1.40  mild | 1.41 – 1.89  moderate | 1.90 – 2.38  high | 2.39 – 2.87  Very high | 2.88 - 4  Extremely high |
