# Supplementary material for: The Borderline Symptom List–Interview: development and psychometric evaluation of an observer-based instrument for assessing symptom severity in borderline personality disorder
Source: Borderline Personal Disord Emot Dysregul. 2025 Aug 28;12:33. doi: 10.1186/s40479-025-00310-6 (PMC12395751; doi:10.1186/s40479-025-00310-6)
Supplement: Supplementary file 4 — Supplementary Material 4 [file 40479_2025_310_MOESM4_ESM.docx]

**Borderline Symptom Liste - Interview**

**BSL-I**

**Deutsche Version**

**Instruktion**

Das BSL-I ist ein halbstrukturiertes Interview, mit dem sich der Schweregrad der Symptome der Borderline-Persönlichkeitsstörung ermitteln lässt.

In der Regel bezieht sich das Interview auf einen Zeitraum von zwei Wochen vor dem Interviewzeitpunkt.

Mit dem BSL-I werden neben der Häufigkeit der Symptome auch das Ausmaß der subjektiv erlebten Belastung, das Verhalten hinsichtlich seiner potenziellen oder realen Konsequenzen sowie die Alltagsbeeinträchtigung systematisch erfasst.

Das BSL-I umfasst 31 Items, von denen die meisten folgende Informationen abfragen:

1. Häufigkeit des Auftretens der Symptome

2. Intensität der Belastung, die mit dem Symptom einhergeht

3. Verhaltenskonsequenzen (Anmerkung: Dieser Aspekt wird nur bei vier Items erfasst)

Ein zusätzliches Item erfasst das Ausmaß der funktionalen Beeinträchtigung der betroffenen Person im Alltag (z.B. Alltagsaktivitäten, soziale Beziehungen und Arbeit oder Ausbildung). Dabei sollte auch die durch Vermeidungsverhalten verursachte Beeinträchtigung berücksichtigt werden.

**Hinweis**: Für die sachgemäße und valide Durchführung des Interviews ist eine fundierte Schulung der Interviewer*innen erforderlich.

**1) Anspannung und intensive unangenehme Emotionen**

**Wörtliche Fragestellung:** „Gab es während der letzten beiden Wochen Momente, längere Episoden oder durchgängige Phasen, während derer Sie innere Anspannung oder unangenehme Gefühle erlebten, die Sie nicht steuern konnten?“

**Falls nicht vorhanden:** Kreuzen Sie bei Häufigkeit „0“ an und gehen Sie zum nächsten Item.

**Falls vorhanden, fragen Sie weiter:**

| **1.1 Häufigkeit** | | | | |
| --- | --- | --- | --- | --- |
| **Spezifische Fragen zur Häufigkeit:** „Wie häufig erlebten Sie diese innere Anspannung, bzw. die unangenehmen Gefühle?“ | | | | |
| **Notizen:** | | | | |
| **Bitte schätzen Sie die Häufigkeit ein:** | | | | |
| 0  Nie | 1  Selten  (1-2-mal in den letzten beiden Wochen) | 2  Gelegentlich  (3-6-mal in den letzten beiden Wochen) | 3  Häufig  (ca. 7 -8-mal in den letzten beiden Wochen) | 4  Sehr häufig  (Fast täglich, oder durchgehend in den letzten beiden Wochen) |
| **1.2 Leidensdruck** | | | | |
| **Spezifische Fragen und Beispiele zum Leidensdruck:** „Inwiefern stellten die innere Anspannung bzw. die unangenehmen Gefühle eine Belastung für Sie dar? Wie intensiv erlebten Sie diese Belastung?“ | | | | |
| **Notizen:** | | | | |
| **Bitte schätzen Sie den Leidensdruck ein:** | | | | |
| 0  Keine  Belastung | 1  Geringe  Belastung | 2  Deutliche Belastung | 3  Ausgeprägte Belastung | 4  Schwere Belastung |

**2) Stimmungsschwankungen**

**Wörtliche Fragestellung:** „Gab es während der letzten beiden Wochen Momente, längere Episoden oder durchgängige Phasen, während derer Sie Stimmungsschwankungen erlebten? D.h. gab es rasche Wechsel zwischen verschiedenen Gefühlen oder innerer Befindlichkeit?“

**Falls nicht vorhanden:** Kreuzen Sie bei Häufigkeit „0“ an und gehen Sie zum nächsten Item.

**Falls vorhanden, fragen Sie weiter:**

| **2.1 Häufigkeit** | | | | |
| --- | --- | --- | --- | --- |
| **Spezifische Fragen zur Häufigkeit:** „Wie häufig traten diese Stimmungsschwankungen auf?“ | | | | |
| **Notizen:** | | | | |
| **Bitte schätzen Sie die Häufigkeit ein:** | | | | |
| 0  Nie | 1  Selten  (1-2-mal in den letzten beiden Wochen) | 2  Gelegentlich  (3-6-mal in den letzten beiden Wochen) | 3  Häufig  (ca. 7 -8-mal in den letzten beiden Wochen) | 4  Sehr häufig  (Fast täglich, oder durchgehend in den letzten beiden Wochen) |
| **2.2 Leidensdruck** | | | | |
| **Spezifische Fragen und Beispiele zum Leidensdruck: „**Inwiefern stellten diese Stimmungsschwankungen eine Belastung für Sie dar? Wie intensiv erlebten Sie diese Belastung?“ | | | | |
| **Notizen:** | | | | |
| **Bitte schätzen Sie den Leidensdruck ein:** | | | | |
| 0  Keine  Belastung | 1  Geringe  Belastung | 2  Deutliche Belastung | 3  Ausgeprägte Belastung | 4  Schwere Belastung |

**3) Emotionale Taubheit**

**Wörtliche Fragestellung: „**Gab es in der letzten beiden Wochen Momente, längere Episoden oder durchgehende Phasen, in denen Sie sich wie abgeschnitten von Ihren Gefühlen erlebten? D.h. Taubheitsempfinden für (Ihre) Gefühle.“

**Falls nicht vorhanden:** Kreuzen Sie bei Häufigkeit „0“ an und gehen Sie zum nächsten Item.

**Falls vorhanden, fragen Sie weiter:**

| **3.1. Häufigkeit** | | | | |
| --- | --- | --- | --- | --- |
| **Spezifische Fragen zur Häufigkeit:** „Wie häufig erlebten Sie dieses Gefühl, von Ihren Emotionen abgeschnitten zu sein?“ | | | | |
| **Notizen:** | | | | |
| **Bitte schätzen Sie die Häufigkeit ein:** | | | | |
| 0  Nie | 1  Selten  (1-2-mal in den letzten beiden Wochen) | 2  Gelegentlich  (3-6-mal in den letzten beiden Wochen) | 3  Häufig  (ca. 7 -8-mal in den letzten beiden Wochen) | 4  Sehr häufig  (Fast täglich, oder durchgehend in den letzten beiden Wochen) |
| **3.2 Leidensdruck** | | | | |
| **Spezifische Fragen und Beispiele zum Leidensdruck: „**Inwiefern stellte das Gefühl der emotionalen Taubheit eine Belastung für Sie dar? Wie intensiv erlebten Sie diese Belastung?“ | | | | |
| **Notizen:** | | | | |
| **Bitte schätzen Sie den Leidensdruck ein:** | | | | |
| 0  Keine  Belastung | 1  Geringe  Belastung | 2  Deutliche Belastung | 3  Ausgeprägte Belastung | 4  Schwere Belastung |

**4) Scham, Schuld**

**Wörtliche Fragestellung: „**Gab es in der letzten beiden Wochen Momente, längere Episoden oder durchgehende Phasen, in denen Sie sich ohne (echten) Grund schämten, schuldig oder böse fühlten?“

**Falls nicht vorhanden:** Kreuzen Sie bei Häufigkeit „0“ an und gehen Sie zum nächsten Item.

**Falls vorhanden, fragen Sie weiter:**

| **4.1. Häufigkeit** | | | | |
| --- | --- | --- | --- | --- |
| **Spezifische Fragen zur Häufigkeit: „**Wie häufig erlebten Sie diese Gefühle?“ | | | | |
| **Notizen:** | | | | |
| **Bitte schätzen Sie die Häufigkeit ein:** | | | | |
| 0  Nie | 1  Selten  (1-2-mal in den letzten beiden Wochen) | 2  Gelegentlich  (3-6-mal in den letzten beiden Wochen) | 3  Häufig  (ca. 7 -8-mal in den letzten beiden Wochen) | 4  Sehr häufig  (Fast täglich, oder durchgehend in den letzten beiden Wochen) |
| **4.2 Leidensdruck** | | | | |
| **Spezifische Fragen und Beispiele zum Leidensdruck: „**Inwiefern stellten diese Gefühle der Scham und der Schuld eine Belastung für Sie dar? Wie intensiv erlebten Sie diese Belastung?“ | | | | |
| **Notizen:** | | | | |
| **Bitte schätzen Sie den Leidensdruck ein:** | | | | |
| 0  Keine  Belastung | 1  Geringe  Belastung | 2  Deutliche Belastung | 3  Ausgeprägte Belastung | 4  Schwere Belastung |

**5) Selbstverachtung oder Selbsthass**

**Wörtliche Fragestellung: „**Gab es in der letzten beiden Wochen Momente, längere Episoden oder gar durchgehende Phasen, in denen Sie sich selbst sehr negativ sahen?“

**Falls nicht vorhanden:** Kreuzen Sie bei Häufigkeit „0“ an und gehen Sie zum nächsten Item.

**Falls vorhanden,** **fragen Sie weiter:** „War diese Selbstverachtung so stark, dass Sie sich vor sich selbst ekelten, sich selbst verachteten oder sich selbst hassten? So, dass Sie sich am liebsten vernichtet hätten?“

| **5.1 Häufigkeit** | | | | |
| --- | --- | --- | --- | --- |
| **Spezifische Fragen zur Häufigkeit: „**Wie häufig erlebten Sie diese Gefühle?“ | | | | |
| **Notizen:** | | | | |
| **Bitte schätzen Sie die Häufigkeit ein:** | | | | |
| 0  Nie | 1  Selten  (1-2-mal in den letzten beiden Wochen) | 2  Gelegentlich  (3-6-mal in den letzten beiden Wochen) | 3  Häufig  (ca. 7 -8-mal in den letzten beiden Wochen) | 4  Sehr häufig  (Fast täglich, oder durchgehend in den letzten beiden Wochen) |
| **5.2 Leidensdruck** | | | | |
| **Spezifische Fragen und Beispiele zum Leidensdruck: „**Inwiefern stellte diese negativen Gefühle gegenüber sich selbst eine Belastung für Sie dar? Wie intensiv erlebten Sie diese Belastung?“ | | | | |
| **Notizen:** | | | | |
| **Bitte schätzen Sie den Leidensdruck ein:** | | | | |
| 0  Keine  Belastung | 1  Geringe  Belastung  (diffuse Selbstabwertung) | 2  Deutliche Belastung (z.B. Selbst-Ekel) | 3  Ausgeprägte Belastung  (z.B. Selbstverachtung) | 4  Schwere Belastung (Selbsthass und Vernichtungsdrang) |

**6) Gereiztheit, Wut und Aggression**

**Wörtliche Fragestellung: „**Gab es in der letzten beiden Wochen Momente, längere Episoden oder gar durchgehende Phasen, in denen Sie sehr gereizt oder wütend waren?“

**Falls nicht vorhanden:** Kreuzen Sie bei Häufigkeit „0“ an und gehen Sie zum nächsten Item.

**Falls vorhanden, fragen Sie weiter:** „Wurden Sie beispielsweise leicht wütend? Griffen Sie jemanden verbal oder körperlich an? Zerstörten Sie Gegenstände?“

| **6.1 Häufigkeit** | | | | |
| --- | --- | --- | --- | --- |
| **Spezifische Fragen zur Häufigkeit: „**Wie häufig erlebten Sie diese Gereiztheit, Wut oder Aggression?“ | | | | |
| **Notizen:** | | | | |
| **Bitte schätzen Sie die Häufigkeit ein:** | | | | |
| 0  Nie | 1  Selten  (1-2-mal in den letzten beiden Wochen) | 2  Gelegentlich  (3-6-mal in den letzten beiden Wochen) | 3  Häufig  (ca. 7 -8-mal in den letzten beiden Wochen) | 4  Sehr häufig  (Fast täglich, oder durchgehend in den letzten beiden Wochen) |
| **6.2 Leidensdruck** | | | | |
| **Spezifische Fragen und Beispiele zum Leidensdruck: „**Inwiefern stellten diese Gereiztheit, die Wut oder die aggressiven Durchbrüche eine Belastung für Sie – oder Ihr Umfeld dar? Wie intensiv erlebten Sie (oder Ihr Umfeld) diese Belastung?“ | | | | |
| **Notizen:** | | | | |
| **Bitte schätzen Sie den Leidensdruck ein:** | | | | |
| 0  Keine  Belastung | 1  Geringe  Belastung | 2  Deutliche Belastung | 3  Ausgeprägte Belastung | 4  Schwere Belastung |
| **6.3 Verhalten** | | | | |
| **Spezifische Fragen und Beispiele zu Konsequenzen des Verhaltens:** „Was waren die Folgen Ihrer Gereiztheit, Ihrer Wut oder Ihrer Aggressionsausbrüche?“ | | | | |
| **Notizen:** | | | | |
| **Bitte schätzen Sie die Konsequenzen des Verhaltens ein:** | | | | |
| 0  Kein entsprechendes Verhalten  vorhanden | 1  Ungefährliches Verhalten; oder geringe Konsequenzen  (z.B. Ablenken, Abbruch von Gesprächen, auch Skills). | 2  Bedenkliches Verhalten; oder deutliche Konsequenzen  (z.B. Beleidigung oder Kränkung anderer). | 3  Bedrohliches Verhalten; oder schwerwiegende Konsequenzen  (z.B. Heftiger Streit, Zerstörung von Mobiliar). | 4  Äußerst bedrohliches Verhalten; oder zerstörerische Konsequenzen  (z.B. Körperliche Angriffe auf andere). |

**7) Hilflosigkeit und Ohnmacht**

**Wörtliche Fragestellung:** „Gab es in der letzten beiden Wochen Momente, längere Episoden oder durchgängige Phasen, in denen Sie sich hilflos oder ohnmächtig gefühlt haben?“

**Falls nicht vorhanden:** Kreuzen Sie bei Häufigkeit „0“ an und gehen Sie zum nächsten Item.

**Falls vorhanden, fragen Sie weiter**: **„**Waren diese Gefühle so stark, dass Sie sich als lebensunfähig erlebten?“

| **7.1 Häufigkeit** | | | | |
| --- | --- | --- | --- | --- |
| **Spezifische Fragen zur Häufigkeit: "**Wie häufig erlebten Sie dieses Gefühl?“ | | | | |
| **Notizen:** | | | | |
| **Bitte schätzen Sie die Häufigkeit ein:** | | | | |
| 0  Nie | 1  Selten  (1-2-mal in den letzten beiden Wochen) | 2  Gelegentlich  (3-6-mal in den letzten beiden Wochen) | 3  Häufig  (ca. 7 -8-mal in den letzten beiden Wochen) | 4  Sehr häufig  (Fast täglich, oder durchgehend in den letzten beiden Wochen) |
| **7.2 Leidensdruck** | | | | |
| **Spezifische Fragen und Beispiele zum Leidensdruck:** “Inwiefern stellten diese Gefühle der Hilflosigkeit und Ohnmacht eine Belastung für Sie dar? Wie intensiv erlebten Sie diese Belastung?“ | | | | |
| **Notizen:** | | | | |
| **Bitte schätzen Sie den Leidensdruck ein:** | | | | |
| 0  Keine  Belastung | 1  Geringe  Belastung | 2  Deutliche Belastung | 3  Ausgeprägte Belastung | 4  Schwere Belastung |

**8) Dissoziation: Derealisation, Depersonalisation, Erinnerungslücken**

**Wörtliche Fragestellung:** „Gab es in der letzten beiden Wochen Momente, längere Episoden oder durchgängige Phasen, in denen Sie dissoziiert waren? Das heißt dass Ihnen die Welt unwirklich vorkam oder Sie sich selbst als fremd erlebt haben, ohne dass Sie dies steuern konnten?“

**Falls nicht vorhanden:** Kreuzen Sie bei Häufigkeit „0“ an und gehen Sie zum nächsten Item.

**Falls vorhanden, fragen Sie weiter:** „Gab es Veränderung im Sehen oder darin, wie Sie Ihren Körper wahnahmen? Spürten Sie noch Schmerzen? Konnten Sie sich noch bewegen? Konnten Sie sprechen? Konnten Sie sich an die entsprechenden Ereignisse erinnern oder gab es Erinnerungslücken? Falls ja, haben Sie sich während dieser Erinnerungslücken gefährdet oder verletzt?“

| **8.1 Häufigkeit** | | | | |
| --- | --- | --- | --- | --- |
| **Spezifische Fragen zur Häufigkeit:** „Wie häufig erlebten Sie diese dissoziativen Episoden?“ | | | | |
| **Notizen:** | | | | |
| **Bitte schätzen Sie die Häufigkeit ein:** | | | | |
| 0  Nie | 1  Selten  (1-2-mal in den letzten beiden Wochen) | 2  Gelegentlich  (3-6-mal in den letzten beiden Wochen) | 3  Häufig  (ca. 7 -8-mal in den letzten beiden Wochen) | 4  Sehr häufig  (Fast täglich, oder durchgehend in den letzten beiden Wochen) |
| **8.2 Leidensdruck** | | | | |
| **Spezifische Fragen und Beispiele zum Leidensdruck:** „Inwiefern stellten diese dissoziativen Episoden eine Belastung für Sie dar? Wie intensiv erlebten Sie diese Belastung?“ | | | | |
| **Notizen:** | | | | |
| **Bitte schätzen Sie den Leidensdruck ein:** | | | | |
| 0  Keine  Belastung | 1  Geringe  Belastung | 2  Deutliche Belastung | 3  Ausgeprägte Belastung | 4  Schwere Belastung |

**9) Selbstverletzungsdrang**

**Wörtliche Fragestellung:** „Gab es in der letzten beiden Wochen Momente, längere Episoden oder durchgängige Phasen, in denen Sie einen starken Drang erlebten, sich selbst zu verletzen (z.B. sich zu schneiden, zu brennen, den Kopf gegen die Wand schlagen, Blutabnehmen etc.)?“

**Falls nicht vorhanden:** Kreuzen Sie bei Häufigkeit „0“ an und gehen Sie zum nächsten Item.

**Falls vorhanden, fragen Sie weiter:** „War der Drang so stark, dass Sie Schwierigkeiten hatten, sich zu kontrollieren?“

| **9.1 Häufigkeit** | | | | |
| --- | --- | --- | --- | --- |
| **Spezifische Fragen zur Häufigkeit: „**Wie häufig hatten Sie den Selbstverletzungsdrang?“ | | | | |
| **Notizen:** | | | | |
| **Bitte schätzen Sie die Häufigkeit ein:** | | | | |
| 0  Nie | 1  Selten  (1-2-mal in den letzten beiden Wochen) | 2  Gelegentlich  (3-6-mal in den letzten beiden Wochen) | 3  Häufig  (ca. 7 -8-mal in den letzten beiden Wochen) | 4  Sehr häufig  (Fast täglich, oder durchgehend in den letzten beiden Wochen) |
| **9.2 Leidensdruck** | | | | |
| **Spezifische Fragen und Beispiele zum Leidensdruck: „**Inwiefern stellte dieser Selbstverletzungsdrang, oder- falls aufgetreten, die Selbstverletzungen eine Belastung für Sie dar? Wie intensiv erlebten Sie diese Belastung?“ | | | | |
| **Notizen:** | | | | |
| **Bitte schätzen Sie den Leidensdruck ein:** | | | | |
| 0  Keine  Belastung | 1  Geringe  Belastung | 2  Deutliche Belastung | 3  Ausgeprägte Belastung | 4  Schwere Belastung |
| **9.3 Verhalten (falls Selbstverletzungen vorhanden)** | | | | |
| **Spezifische Fragen und Beispiele zu Konsequenzen: „**Haben Sie dem Selbstverletzungsdrang nachgegeben? Falls ja, was taten Sie? Wie gefährlich waren die Selbstverletzungen? Welche Folgen hatten Ihre Selbstverletzungen? Was waren die gefährlichsten Konsequenzen?“ | | | | |
| **Notizen:** | | | | |
| **Bitte schätzen Sie die Konsequenzen der Selbstverletzungen ein:** | | | | |
| 0  Keine Selbst-verletzungen | 1  Ungefährliches Verhalten; oder geringe Konsequenzen (z.B. oberflächliches Ritzen) | 2  Bedenkliches Verhalten; oder deutliche Konsequenzen  (z.B. Eigenständige Wundversorgung) | 3  Bedrohliches Verhalten; oder schwerwiegende Konsequenzen  (z.B. Wundversorgung durch Arzt notwendig) | 4  Äußerst bedrohliches Verhalten; oder zerstörerische Konsequenzen  (potenziell lebensgefährlich: z.B. das Bestehen von massiven Blutungen; Öffnung der Bauchwand, Schlucken von Rasierklingen etc.) |

**10) Suizidgedanken**

**Wörtliche Fragestellung:** „Gab es in der letzten beiden Wochen Momente, längere Episoden oder durchgängige Phasen, mit drängenden Suizidgedanken?“

**Falls nicht vorhanden:** Kreuzen Sie bei Häufigkeit „0“ an und gehen Sie zum nächsten Item

**Falls vorhanden, fragen Sie weiter:** „Handelte es sich um ständig aufflackernde Suizidgedanken ohne konkrete Pläne oder gab es konkrete Pläne? Machten Sie Vorbereitung wie bspw. das Schreiben von Abschiedsbriefen? Informierten Sie Personen aus Ihrer Umgebung? Haben Sie einen Suizidversuch begangen?“

| **10.1 Häufigkeit** | | | | |
| --- | --- | --- | --- | --- |
| **Spezifische Fragen zur Häufigkeit:** Wie häufig hatten Sie Suizidgedanken? | | | | |
| **Notizen:** | | | | |
| **Bitte schätzen Sie die Häufigkeit ein:** | | | | |
| 0  Nie | 1  Selten  (1-2-mal in den letzten beiden Wochen) | 2  Gelegentlich  (3-6-mal in den letzten beiden Wochen) | 3  Häufig  (ca. 7 -8-mal in den letzten beiden Wochen) | 4  Sehr häufig  (Fast täglich, oder durchgehend in den letzten beiden Wochen) |
| **10.2 Leidensdruck** | | | | |
| **Spezifische Fragen und Beispiele zum Leidensdruck:** „Inwiefern stellten die Suizidgedanken eine Belastung für Sie dar? Konnten Sie diese Gedanken kontrollieren? Wie intensiv erlebten Sie diese Belastung?“ | | | | |
| **Notizen:** | | | | |
| **Bitte schätzen Sie den Leidensdruck durch die Suizidgedanken ein:** | | | | |
| 0  Keine  Belastung | 1  Geringe  Belastung | 2  Deutliche Belastung | 3  Ausgeprägte Belastung | 4  Schwere Belastung |
| **10.3 Verhaltens (falls Suizidversuche vorhanden)** | | | | |
| **Spezifische Fragen und Beispiele zu Konsequenzen: „**Haben Sie etwas Spezifisches unternommen, um Ihre Suizidgedanken zu stoppen oder ihre Intensität zu verringern? Haben Sie einen Suizidversuch unternommen? Was haben Sie getan?" | | | | |
| **Notizen:** | | | | |
| **Bitte schätzen Sie die Konsequenzen der Selbstverletzungen ein:** | | | | |
| 0  Kein Suizidversuch | 1  Ungefährliches Verhalten; oder geringe Konsequenzen (z.B. begonnen, Medikament einzunehmen, dann sofort unterbrochen oder Hilfe gesucht). | 2  Bedenkliches Verhalten; oder deutliche Konsequenzen  (z.B. mit Suizidgedanken zu Brücke gefahren und dort telefoniert). | 3  Bedrohliches Verhalten; oder schwerwiegende Konsequenzen  (z.B. potenziell letale Dosis Medikamente eingenommen, dann Hilfe organisiert). | 4  Äußerst bedrohliches Verhalten; oder zerstörerische Konsequenzen  (Lebensgefährlicher Versuch, durch Zufall gerettet). |
|  | | | | |

**11) Bedrohungsgefühl**

**Wörtliche Fragestellung:** „Gab es in der letzten beiden Wochen Momente, längere Episoden oder durchgängige Phasen, in denen Sie sich einer unbestimmten Gefahr ausgesetzt fühlten?“

**Falls nicht vorhanden:** Kreuzen Sie bei Häufigkeit „0“ an und gehen Sie zum nächsten Item (Falls es sich um eine realistische, reale Bedrohung handelt, bitte 0 ankreuzen).

**Falls vorhanden, fragen Sie weiter:** „Fühlten Sie sich bedroht, ohne genauer zu wissen, woher dieses Gefühl kam? Erlebten Sie Momente oder Episoden, in denen Sie sich in einer “Hab-acht-Stellung" befanden? Bezog sich Ihr Bedrohungsgefühl auf konkrete Personen oder Ereignisse oder handelte es sich eher um ein unbestimmtes Gefühl? Gab es einen realistischen Hintergrund für das Gefühl?“

| **11.1 Häufigkeit** | | | | |
| --- | --- | --- | --- | --- |
| **Spezifische Fragen zur Häufigkeit: „**Wie häufig hatten Sie das Bedrohungsgefühl?“ | | | | |
| **Notizen:** | | | | |
| **Bitte schätzen Sie die Häufigkeit ein:** | | | | |
| 0  Nie | 1  Selten  (1-2-mal in den letzten beiden Wochen) | 2  Gelegentlich  (3-6-mal in den letzten beiden Wochen) | 3  Häufig  (ca. 7 -8-mal in den letzten beiden Wochen) | 4  Sehr häufig  (Fast täglich, oder durchgehend in den letzten beiden Wochen) |
| **11.2 Leidensdruck** | | | | |
| **Spezifische Fragen und Beispiele zum Leidensdruck: „**Inwiefern stellte dieses Bedrohungsgefühl eine Belastung für Sie dar? Wie intensiv erlebten Sie diese Belastung?“ | | | | |
| **Notizen:** | | | | |
| **Bitte schätzen Sie den Leidensdruck ein:** | | | | |
| 0  Keine  Belastung | 1  Geringe  Belastung | 2  Deutliche Belastung | 3  Ausgeprägte Belastung | 4  Schwere Belastung |

**12) Einsamkeit**

**Wörtliche Fragestellung:** “Gab es in der letzten beiden Wochen Momente, längere Episoden oder durchgehende Phasen, in denen Sie sich einsam fühlten?“

**Falls nicht vorhanden:** Kreuzen Sie bei Häufigkeit „0“ an und gehen Sie zum nächsten Item (Falls es sich um eine realistische, reale Bedrohung handelt, bitte 0 ankreuzen).

**Falls vorhanden, fragen Sie weiter:** **„**Trat dieses Gefühl auch auf, wenn Sie unter Menschen waren? Konnten Sie allein sein oder verstärkte dies das Gefühl der Einsamkeit?

| **12.1 Häufigkeit** | | | | |
| --- | --- | --- | --- | --- |
| **Spezifische Fragen zur Häufigkeit: „**Wie häufig hatten das Gefühl der Einsamkeit?“ | | | | |
| **Notizen:** | | | | |
| **Bitte schätzen Sie die Häufigkeit ein:** | | | | |
| 0  Nie | 1  Selten  (1-2-mal in den letzten beiden Wochen) | 2  Gelegentlich  (3-6-mal in den letzten beiden Wochen) | 3  Häufig  (ca. 7 -8-mal in den letzten beiden Wochen) | 4  Sehr häufig  (Fast täglich, oder durchgehend in den letzten beiden Wochen) |
| **12.2 Leidensdruck** | | | | |
| **Spezifische Fragen und Beispiele zum Leidensdruck:** „Inwiefern stellte dieses Gefühl der Einsamkeit eine Belastung für Sie dar? Wie intensiv erlebten Sie diese Belastung?“ | | | | |
| **Notizen:** | | | | |
| **Bitte schätzen Sie den Leidensdruck ein:** | | | | |
| 0  Keine  Belastung | 1  Geringe  Belastung | 2  Deutliche Belastung | 3  Ausgeprägte Belastung | 4  Schwere Belastung |

**13) Angst vor Verlassenwerden**

**Wörtliche Fragestellung:** „Gab es in der letzten beiden Wochen Momente, längere Episoden oder auch durchgängige Phasen, in denen Sie Angst oder Sorgen hatten, dass eine Ihnen nahestehende Person Sie verlassen könnte? Oder hatten Sie das Gefühl allein gelassen zu werden?

**Falls nicht vorhanden:** Kreuzen Sie bei Häufigkeit „0“ an und beenden Sie das Interview

**Falls vorhanden, fragen Sie weiter:**

| **13.1 Häufigkeit** | | | | |
| --- | --- | --- | --- | --- |
| **Spezifische Fragen zur Häufigkeit:** "Falls ja, wie oft hatten Sie Angst oder Sorge, dass eine Ihnen nahestehende Person Sie verlassen könnte?“ | | | | |
| **Notizen:** | | | | |
| **Bitte schätzen Sie die Häufigkeit ein:** | | | | |
| 0  Nie | 1  Selten  (1-2-mal in den letzten beiden Wochen) | 2  Gelegentlich  (3-6-mal in den letzten beiden Wochen) | 3  Häufig  (ca. 7 -8-mal in den letzten beiden Wochen) | 4  Sehr häufig  (Fast täglich, oder durchgehend in den letzten beiden Wochen) |
| **13.2 Leidensdruck** | | | | |
| **Spezifische Fragen und Beispiele zum Leidensdruck: „**Inwiefern stellten diese Angst und Sorgen eine Belastung für Sie dar? Wie intensiv erlebten Sie diese Belastung?“ | | | | |
| **Notizen:** | | | | |
| **Bitte schätzen Sie den Leidensdruck ein:** | | | | |
| 0  Keine  Belastung | 1  Geringe  Belastung | 2  Deutliche Belastung | 3  Ausgeprägte Belastung | 4  Schwere Belastung |

**14) Identität: Kohärenz und Konsistenz**

**Wörtliche Fragestellung:** „Gab es in der letzten beiden Wochen Momente, längere Episoden oder durchgängige Phasen, wo Sie das Gefühl hatten, dass Sie nicht wissen, wer Sie eigentlich sind, oder was für Sie wichtig und unwichtig ist? Änderten sich Ihre Werte und Ziele kurzfristig?“

**Falls nicht vorhanden:** Kreuzen Sie bei Häufigkeit „0“ an und gehen Sie zum nächsten Item.

**Falls vorhanden, fragen Sie weiter:** „Hatten Sie Sehnsucht nach innerer Sicherheit und den drängenden Wunsch nach einem stabilen inneren Kern? Fühlten Sie sich Ihren Schwankungen, Gedanken und Ihrer Umgebung ausgeliefert?“

| **14.1 Häufigkeit** | | | | |
| --- | --- | --- | --- | --- |
| **Spezifische Fragen zur Häufigkeit:** Wie häufig hatten das Gefühl der Unsicherheit? | | | | |
| **Notizen:** | | | | |
| **Bitte schätzen Sie die Häufigkeit ein:** | | | | |
| 0  Nie | 1  Selten  (1-2-mal in den letzten beiden Wochen) | 2  Gelegentlich  (3-6-mal in den letzten beiden Wochen) | 3  Häufig  (ca. 7 -8-mal in den letzten beiden Wochen) | 4  Sehr häufig  (Fast täglich, oder durchgehend in den letzten beiden Wochen) |
| **14.2 Leidensdruck** | | | | |
| **Spezifische Fragen und Beispiele zum Leidensdruck: „**Inwiefern stellte dieses Gefühl der Unsicherheit oder Sehnsucht nach innerer Sicherheit eine Belastung für Sie dar? Wie intensiv erlebten Sie diese Belastung?“ | | | | |
| **Notizen:** | | | | |
| **Bitte schätzen Sie den Leidensdruck ein:** | | | | |
| 0  Keine  Belastung | 1  Geringe  Belastung | 2  Deutliche Belastung | 3  Ausgeprägte Belastung | 4  Schwere Belastung |

**15) Innere Leere**

**Wörtliche Fragestellung:** „Gab es in der letzten beiden Wochen Momente, längere Episoden oder durchgängige Phasen, in denen Sie in sich eine unangenehme innere Leere verspürten? Manche beschreiben dies als eine Art schwarzes Loch.

**Falls nicht vorhanden:** Kreuzen Sie bei Häufigkeit „0“ an und gehen Sie zum nächsten Item.

**Falls vorhanden, fragen Sie weiter:** Können Sie das genauer beschreiben?“

| **15.1 Häufigkeit** | | | | |
| --- | --- | --- | --- | --- |
| **Spezifische Fragen zur Häufigkeit: „**Wie häufig hatten Sie diese Momente oder Episoden von Innerer Leere?“ | | | | |
| **Notizen:** | | | | |
| **Bitte schätzen Sie die Häufigkeit ein:** | | | | |
| 0  Nie | 1  Selten  (1-2-mal in den letzten beiden Wochen) | 2  Gelegentlich  (3-6-mal in den letzten beiden Wochen) | 3  Häufig  (ca. 7 -8-mal in den letzten beiden Wochen) | 4  Sehr häufig  (Fast täglich, oder durchgehend in den letzten beiden Wochen) |
| **15.2 Leidensdruck** | | | | |
| **Spezifische Fragen und Beispiele zum Leidensdruck: „**Inwiefern stellte dieses Gefühl der Inneren Leere eine Belastung für Sie dar? Wie intensiv erlebten Sie diese Belastung?“ | | | | |
| **Notizen:** | | | | |
| **Bitte schätzen Sie den Leidensdruck ein:** | | | | |
| 0  Keine  Belastung | 1  Geringe  Belastung | 2  Deutliche Belastung | 3  Ausgeprägte Belastung | 4  Schwere Belastung |

**16) Zweifel an der eigenen Urteilsfähigkeit**

**Wörtliche Fragestellung:** „Gab es in der letzten beiden Wochen Momente, längere Episoden oder durchgängige Phasen, in denen Sie an Ihrer eigenen Urteilfähigkeit gezweifelt haben, Dinge, Ereignisse, oder Menschen richtig einzuschätzen, weil Sie an Ihrer Wahrnehmung, Einschätzung oder Beurteilung zweifelten?“

**Falls nicht vorhanden:** Kreuzen Sie bei Häufigkeit „0“ an und gehen Sie zum nächsten Item.

**Falls vorhanden, fragen Sie weiter:**

| **16.1 Häufigkeit** | | | | |
| --- | --- | --- | --- | --- |
| **Spezifische Fragen zur Häufigkeit: „**Wie häufig hatten Sie in den letzten beiden Wochen diese Momente oder Episoden voller Zweifel an Ihrer eigenen Urteilsfähigkeit?“ | | | | |
| **Notizen:** | | | | |
| **Bitte schätzen Sie die Häufigkeit ein:** | | | | |
| 0  Nie | 1  Selten  (1-2-mal in den letzten beiden Wochen) | 2  Gelegentlich  (3-6-mal in den letzten beiden Wochen) | 3  Häufig  (ca. 7 -8-mal in den letzten beiden Wochen) | 4  Sehr häufig  (Fast täglich, oder durchgehend in den letzten beiden Wochen) |
| **16.2 Leidensdruck** | | | | |
| **Spezifische Fragen und Beispiele zum Leidensdruck: „**Inwiefern stellten diese Zweifel am eigenen Urteil eine Belastung für Sie dar? Wie intensiv erlebten Sie diese Belastung?“ | | | | |
| **Notizen:** | | | | |
| **Bitte schätzen Sie den Leidensdruck ein:** | | | | |
| 0  Keine  Belastung | 1  Geringe  Belastung | 2  Deutliche Belastung | 3  Ausgeprägte Belastung | 4  Schwere Belastung |

**17) Wertlosigkeit**

**Wörtliche Fragestellung:** „Gab es in der letzten beiden Wochen Momente, längere Episoden oder durchgängige Phasen, in denen Sie das Gefühl hatten, nichts wert zu sein, völlig bedeutungslos zu sein, oder dass Sie es nicht verdient hätten, auf der Welt zu sein? Also dass die Welt ohne Sie eine bessere Welt wäre?

**Falls nicht vorhanden:** Kreuzen Sie bei Häufigkeit „0“ an und gehen Sie zum nächsten Item.

**Falls vorhanden, fragen Sie weiter:**

| **17.1 Häufigkeit** | | | | |
| --- | --- | --- | --- | --- |
| **Spezifische Fragen zur Häufigkeit: „**Wie häufig erlebten Sie in den letzten beiden Wochen diese Momente oder Episoden der Wertlosigkeit oder fehlenden Lebensberechtigung?“ | | | | |
| **Notizen:** | | | | |
| **Bitte schätzen Sie die Häufigkeit ein:** | | | | |
| 0  Nie | 1  Selten  (1-2-mal in den letzten beiden Wochen) | 2  Gelegentlich  (3-6-mal in den letzten beiden Wochen) | 3  Häufig  (ca. 7 -8-mal in den letzten beiden Wochen) | 4  Sehr häufig  (Fast täglich, oder durchgehend in den letzten beiden Wochen) |
| **17.2 Leidensdruck** | | | | |
| **Spezifische Fragen und Beispiele zum Leidensdruck: „**Inwiefern stellte dieses Gefühl der Wertlosigkeit eine Belastung für Sie dar? Wie intensiv erlebten Sie diese Belastung?“ | | | | |
| **Notizen:** | | | | |
| **Bitte schätzen Sie den Leidensdruck ein:** | | | | |
| 0  Keine  Belastung | 1  Geringe  Belastung | 2  Deutliche Belastung | 3  Ausgeprägte Belastung | 4  Schwere Belastung |

**18) Fehlen von Selbstvertrauen, Selbstwirksamkeit, Versagensängste**

**Wörtliche Fragestellung: „**Gab es in der letzten beiden Wochen Momente, längere Episoden oder durchgängige Phasen, in denen sie das Gefühl hatten, dass Sie die einfachsten Dinge nicht erledigen können? Oder haben Sie erlebt, dass sie nach einer Kritik, oder einem Fehler emotional eingebrochen sind?“

**Falls nicht vorhanden:** Kreuzen Sie bei Häufigkeit „0“ an und gehen Sie zum nächsten Item.

**Falls vorhanden, fragen Sie weiter:**

| **18.1 Häufigkeit** | | | | |
| --- | --- | --- | --- | --- |
| **Spezifische Fragen zur Häufigkeit: „**Wie häufig erlebten Sie in den letzten beiden Wochen dieses Gefühl, eine Versagerin/ein Versager zu sein?“ | | | | |
| **Notizen:** | | | | |
| **Bitte schätzen Sie die Häufigkeit ein:** | | | | |
| 0  Nie | 1  Selten  (1-2-mal in den letzten beiden Wochen) | 2  Gelegentlich  (3-6-mal in den letzten beiden Wochen) | 3  Häufig  (ca. 7 -8-mal in den letzten beiden Wochen) | 4  Sehr häufig  (Fast täglich, oder durchgehend in den letzten beiden Wochen) |
| **18.2 Leidensdruck** | | | | |
| **Spezifische Fragen und Beispiele zum Leidensdruck: „**Inwiefern stellte dieses Gefühl, zu versagen, eine Belastung für Sie dar? Wie intensiv erlebten Sie diese Belastung?“ | | | | |
| **Notizen:** | | | | |
| **Bitte schätzen Sie den Leidensdruck ein:** | | | | |
| 0  Keine  Belastung | 1  Geringe  Belastung | 2  Deutliche Belastung | 3  Ausgeprägte Belastung | 4  Schwere Belastung |

**19) Negatives Körperselbst**

**Wörtliche Fragestellung:** „Gab es in der letzten beiden Wochen Momente, längere Episoden oder durchgängige Phasen, in denen Sie Ihren Körper als völlig fremd oder ekelhaft erlebten?“

**Falls nicht vorhanden:** Kreuzen Sie bei Häufigkeit „0“ an und gehen Sie zum nächsten Item.

**Falls vorhanden, fragen Sie weiter**: „Hatten Sie das Gefühl, Ihren Körper zu hassen? Hatten Sie den Drang, Ihren Körper zu zerstören?“

| **19.1 Häufigkeit** | | | | |
| --- | --- | --- | --- | --- |
| **Spezifische Fragen zur Häufigkeit: „**Wie häufig hatten Sie in den letzten beiden Wochen Momente oder Episoden, während derer Sie Ihren Köper fremd, ekelhaft oder hassenswert fanden?“ | | | | |
| **Notizen:** | | | | |
| **Bitte schätzen Sie die Häufigkeit ein:** | | | | |
| 0  Nie | 1  Selten  (1-2-mal in den letzten beiden Wochen) | 2  Gelegentlich  (3-6-mal in den letzten beiden Wochen) | 3  Häufig  (ca. 7 -8-mal in den letzten beiden Wochen) | 4  Sehr häufig  (Fast täglich, oder durchgehend in den letzten beiden Wochen) |
| **19.2 Leidensdruck** | | | | |
| **Spezifische Fragen und Beispiele zum Leidensdruck: „**Inwiefern stellte dieses negative Gefühl gegenüber Ihrem Körper eine Belastung für Sie dar? Wie intensiv erlebten Sie diese Belastung?“ | | | | |
| **Notizen:** | | | | |
| **Bitte schätzen Sie den Leidensdruck ein:** | | | | |
| 0  Keine  Belastung | 1  Geringe  Belastung | 2  Deutliche Belastung | 3  Ausgeprägte Belastung | 4  Schwere Belastung |

**20) Probleme mit Vertrauen, prosozialen Signalen und emotionaler Nähe**

**Wörtliche Fragestellung:** „Gab es in der letzten beiden Wochen Momente, längere Episoden oder durchgängige Phasen, in denen es für Sie schwierig war, anderen Menschen zu vertrauen? Oder hatten Sie Probleme damit, freundliche Signale aus Ihrer Umwelt anzunehmen. Haben Sie emotionale Nähe als schwer erträglich empfunden?“

**Falls nicht vorhanden:** Kreuzen Sie bei Häufigkeit „0“ an und gehen Sie zum nächsten Item.

**Falls vorhanden, fragen Sie weiter:**

| **20.1 Häufigkeit** | | | | |
| --- | --- | --- | --- | --- |
| **Spezifische Fragen zur Häufigkeit: „**Wie häufig hatten Sie in den letzten beiden Wochen Momente oder Episoden mit Schwierigkeiten, anderen zu vertrauten, freundliche Signale anzunehmen, oder emotionale Nähe zuzulassen?“ | | | | |
| **Notizen:** | | | | |
| **Bitte schätzen Sie die Häufigkeit ein:** | | | | |
| 0  Nie | 1  Selten  (1-2-mal in den letzten beiden Wochen) | 2  Gelegentlich  (3-6-mal in den letzten beiden Wochen) | 3  Häufig  (ca. 7 -8-mal in den letzten beiden Wochen) | 4  Sehr häufig  (Fast täglich, oder durchgehend in den letzten beiden Wochen) |
| **20.2 Leidensdruck** | | | | |
| **Spezifische Fragen und Beispiele zum Leidensdruck: „**Inwiefern stellten diese Schwierigkeiten anderen zu vertrauen eine Belastung für Sie dar? Wie intensiv erlebten Sie diese Belastung?“ | | | | |
| **Notizen:** | | | | |
| **Bitte schätzen Sie den Leidensdruck ein:** | | | | |
| 0  Keine  Belastung | 1  Geringe  Belastung | 2  Deutliche Belastung | 3  Ausgeprägte Belastung | 4  Schwere Belastung |

**21) Soziale Ausgrenzung, Kränkung, Demütigung**

**Wörtliche Fragestellung:** „Gab es in der letzten beiden Wochen Momente, längere Episoden oder auch durchgängige Phasen, in denen Sie das Gefühl hatten, von anderen aus einer Gemeinschaft ausgeschlossen oder gekränkt zu werden? Gab es Momente, in denen Sie sich gedemütigt fühlten?“

**Falls nicht vorhanden:** Kreuzen Sie bei Häufigkeit „0“ an und gehen Sie zum nächsten Item.

**Falls vorhanden, fragen Sie weiter:** „Können Sie diese Momente beschreiben? Wie lange hielt das Gefühl des sozialen Ausschlusses, der Kränkung oder Demütigung an?“

| **21.1 Häufigkeit** | | | | |
| --- | --- | --- | --- | --- |
| **Spezifische Fragen zur Häufigkeit: „**Wie häufig erlebten Sie diese Gefühle?“ | | | | |
| **Notizen:** | | | | |
| **Bitte schätzen Sie die Häufigkeit ein:** | | | | |
| 0  Nie | 1  Selten  (1-2-mal in den letzten beiden Wochen) | 2  Gelegentlich  (3-6-mal in den letzten beiden Wochen) | 3  Häufig  (ca. 7 -8-mal in den letzten beiden Wochen) | 4  Sehr häufig  (Fast täglich, oder durchgehend in den letzten beiden Wochen) |
| **21.2 Psychisches Erleben: Leidensdruck** | | | | |
| **Spezifische Fragen und Beispiele zum Leidensdruck:** „Inwiefern stellten diese Erfahrungen eine Belastung für Sie dar? Wie intensiv erlebten Sie diese Belastung?“ | | | | |
| **Notizen:** | | | | |
| **Bitte schätzen Sie den Leidensdruck ein:** | | | | |
| 0  Keine  Belastung | 1  Geringe  Belastung | 2  Deutliche Belastung | 3  Ausgeprägte Belastung | 4  Schwere Belastung |

**22) Fremdheitsgefühl (Alienation)**

**Wörtliche Fragestellung:** „Gab es in der letzten beiden Wochen Momente, längere Episoden oder auch durchgängige Phasen, in denen das Sie das Gefühl hatten, anders zu sein als alle anderen? Anders zu denken oder zu fühlen oder einfach nicht „dazuzugehören“? Spürten Sie so etwas wie einen Riss zwischen sich und der Welt oder das Gefühl nicht mit anderen verbunden zu sein? Spürten Sie eine Art „Heimweh“ nach Ihren Mitmenschen?“

**Falls nicht vorhanden:** Kreuzen Sie bei Häufigkeit „0“ an und gehen Sie zum nächsten Item.

**Falls vorhanden, fragen Sie weiter:**

| **22.1 Häufigkeit** | | | | |
| --- | --- | --- | --- | --- |
| **Spezifische Fragen zur Häufigkeit: „**Wie häufig erlebten Sie dieses Gefühl des Fremdseins? War das fast täglich der Fall?“ | | | | |
| **Notizen:** | | | | |
| **Bitte schätzen Sie die Häufigkeit ein:** | | | | |
| 0  Nie | 1  Selten  (1-2-mal in den letzten beiden Wochen) | 2  Gelegentlich  (3-6-mal in den letzten beiden Wochen) | 3  Häufig  (ca. 7 -8-mal in den letzten beiden Wochen) | 4  Sehr häufig  (Fast täglich, oder durchgehend in den letzten beiden Wochen) |
| **22.2 Leidensdruck** | | | | |
| **Spezifische Fragen und Beispiele zum Leidensdruck:** „Inwiefern stellte dieses Fremdheitsgefühl eine Belastung für Sie dar? Wie intensiv erlebten Sie diese Belastung?“ | | | | |
| **Notizen:** | | | | |
| **Bitte schätzen Sie den Leidensdruck ein:** | | | | |
| 0  Keine  Belastung | 1  Geringe  Belastung | 2  Deutliche Belastung | 3  Ausgeprägte Belastung | 4  Schwere Belastung |

**23) Intrusionen & Flashbacks**

**Wörtliche Fragestellung:** „Gab es in der letzten beiden Wochen Momente, längere Episoden oder auch durchgängige Phasen, während derer sich alte traumatische oder invalidierende (kränkende) Erfahrungen in Ihr Gedächtnis drängten, die Sie schlecht kontrollieren konnten? Fühlte es sich so ab, als ob das Erlebnis im Hier und Jetzt wieder stattfände?“

F**alls nicht vorhanden:** Kreuzen Sie bei Häufigkeit „0“ an und gehen Sie zum nächsten Item.

**Falls vorhanden, fragen Sie weiter: „**Wussten Sie, dass es sich um Erinnerungen handelte, oder hatten Sie jeden Kontakt zu Realität verloren?

| **23.1. Häufigkeit** | | | | |
| --- | --- | --- | --- | --- |
| **Spezifische Fragen zur Häufigkeit: „**Falls ja, wie häufig hatten Sie diese Intrusionen oder Flashbacks? War das fast täglich der Fall?“ | | | | |
| **Notizen:** | | | | |
| **Bitte schätzen Sie die Häufigkeit ein:** | | | | |
| 0  Nie | 1  Selten  (1-2-mal in den letzten beiden Wochen) | 2  Gelegentlich  (3-6-mal in den letzten beiden Wochen) | 3  Häufig  (ca. 7 -8-mal in den letzten beiden Wochen) | 4  Sehr häufig  (Fast täglich, oder durchgehend in den letzten beiden Wochen) |
| **23.2. Leidensdruck** | | | | |
| **Spezifische Fragen und Beispiele zum Leidensdruck:** „Inwiefern stellten diese Intrusionen oder Flashbacks eine Belastung für Sie dar? Wie intensiv erlebten Sie diese Belastung?“ | | | | |
| **Notizen:** | | | | |
| **Bitte schätzen Sie den Leidensdruck ein:** | | | | |
| 0  Keine  Belastung | 1  Geringe  Belastung | 2  Deutliche Belastung | 3  Ausgeprägte Belastung | 4  Schwere Belastung |

**24) Akustische & Optische (Pseudo-) Halluzinationen**

**Wörtliche Fragestellung: „**Gab es in der letzten beiden Wochen Momente, längere Episoden oder auch durchgängige Phasen, in denen Sie Stimmen hörten (im Kopf oder außerhalb des Kopfes), von denen Sie wussten, dass sie nicht real sind? Oder haben Personen, Wesen oder Gestalten gesehen, von denen Sie wussten, dass sie nicht wirklich sind?“

**Falls nicht vorhanden:** Kreuzen Sie bei Häufigkeit „0“ an und gehen Sie zum nächsten Item.

**Falls vorhanden, fragen Sie weiter**: „Falls Sie diese Stimmen hörten, waren das Ihre eigenen Gedanken, die laut wurden, oder die Stimmen von anderen Personen? Wurden die Stimmen laut, um sie zu beschimpfen, z.B. wenn sei sich etwas Gutes tun wollen? Gaben Ihnen diese Stimmen Anweisungen, fordern Sie diese Stimmen auf, sich etwas anzutun, oder jemand anderen anzugreifen?

Falls Sie Wesen oder Dinge sahen, die es nicht gibt, wirkten diese Personen bedrohlich auf Sie? Haben Sie erlebt, dass sich das Gesicht, oder die Hände oder auch die Stimme einer vertrauten Person plötzlich veränderten, sich verwandelten und bedrohlich wurden?“

| **24.1. Häufigkeit** | | | | |
| --- | --- | --- | --- | --- |
| **Spezifische Fragen zur Häufigkeit: „**Falls ja, wie häufig hatten Sie diese akustische oder optische Pseudohalluzinationen?“ | | | | |
| **Notizen:** | | | | |
| **Bitte schätzen Sie die Häufigkeit ein:** | | | | |
| 0  Nie | 1  Selten  (1-2-mal in den letzten beiden Wochen) | 2  Gelegentlich  (3-6-mal in den letzten beiden Wochen) | 3  Häufig  (ca. 7 -8-mal in den letzten beiden Wochen) | 4  Sehr häufig  (Fast täglich, oder durchgehend in den letzten beiden Wochen) |
| **24.2 Leidensdruck** | | | | |
| **Spezifische Fragen und Beispiele zum Leidensdruck:** „Inwiefern stellten diese Stimmen oder Bilder eine Belastung für Sie dar? Wie intensiv erlebten Sie diese Belastung?“  Hatten Sie das Gefühl den Stimmen folgen zu müssen oder keine Kontrolle über ihr Verhalten mehr zu haben?“ | | | | |
| **Notizen:** | | | | |
| **Bitte schätzen Sie den Leidensdruck ein:** | | | | |
| 0  Keine  Belastung | 1  Geringe  Belastung | 2  Deutliche Belastung | 3  Ausgeprägte Belastung | 4  Schwere Belastung |

**25) Verhaltenskontrolle (ohne Selbstverletzungen)**

**Wörtliche Fragestellung:** „Gab es in der letzten beiden Wochen Momente, längere Episoden oder auch durchgängige Phasen, in denen Sie Schwierigkeiten hatten, Ihr Verhalten zu kontrollieren (Selbstverletzungen sind hier nicht gemeint)? So dass Sie Dinge machten, die Sie später bereuten? Gab es zum Beispiel Ess- und/oder Brechanfällen; Hochrisikoverhalten (Rasend Autofahren; Brückengeländer, Bahngleise etc.); Schwere Betrunkenheit; Drogen (u/o nicht verschriebene Medikamentenkonsum); Schwierige sexuelle Kontakte (mit hohem Risiko oder Reue verbunden)? Blutabnehmen?“

**Falls nicht vorhanden:** Kreuzen Sie bei Häufigkeit „0“ an und gehen Sie zum nächsten Item.

**Falls vorhanden, fragen Sie weiter:** „Falls ja, können Sie mir Beispiele nennen?“

| **25.1 Häufigkeit** | | | | |
| --- | --- | --- | --- | --- |
| **Spezifische Fragen zur Häufigkeit:“** Falls ja, wie häufig kam es zu diesem schwer kontrollierbarem Problemverhalten? Wie viele verschiedene Verhaltensweisen sind betroffen?“ | | | | |
| **Notizen:** | | | | |
| **Bitte schätzen Sie die Häufigkeit ein:** | | | | |
| 0  Nie | 1  Selten  (1-2-mal in den letzten beiden Wochen) | 2  Gelegentlich  (3-6-mal in den letzten beiden Wochen) | 3  Häufig  (ca. 7 -8-mal in den letzten beiden Wochen) | 4  Sehr häufig  (Fast täglich, oder durchgehend in den letzten beiden Wochen) |
| **25.2 Leidensdruck** | | | | |
| **Spezifische Fragen und Beispiele zum Leidensdruck:** „Inwiefern stellten diese Probleme der Verhaltenskontrolle eine Belastung für Sie dar? Wie intensiv erlebten Sie diese Belastung?“ | | | | |
| **Notizen:** | | | | |
| **Bitte schätzen Sie den Leidensdruck ein:** | | | | |
| 0  Keine  Belastung | 1  Geringe  Belastung | 2  Deutliche Belastung | 3  Ausgeprägte Belastung | 4  Schwere Belastung |
| **25.3 Verhalten** | | | | |
| **Spezifische Fragen und Beispiele zu Konsequenzen des Verhaltens: „**Falls ja, was haben Sie getan und welche Konsequenzen hatte Ihr Verhalten? Bei mehreren Verhaltensweisen denken Sie bitte an das Verhalten mit den gefährlichsten Konsequenzen.“ | | | | |
| **Notizen:** | | | | |
| **Bitte schätzen Sie die Konsequenzen des Verhaltens ein:** | | | | |
| 0  Kein Problemverhalten  vorhanden | 1  Ungefährliches Verhalten oder geringe Konsequenzen (z.B. Ablenkung, Essanfälle, auch Skills). | 2  Bedenkliches Verhalten oder deutliche Konsequenzen (z.B. Ungeschützter Geschlechtsverkehr mit Fremden). | 3  Bedrohliches Verhalten oder schwerwiegende Konsequenzen (z.B. Rasen auf der Autobahn). | 4  Äußerst bedrohliches Verhalten; oder zerstörerische Konsequenzen  (z.B. Balancieren auf hohem Brückengeländer). |

**26) Hoffnung und Zuversicht**

**Wörtliche Fragestellung:** „Gab es in der letzten beiden Wochen Momente, längere Episoden oder auch durchgängige Phasen, während derer Sie sich zuversichtlich und hoffnungsvoll fühlten, dass Sie Ihr Leben meistern können?

**Falls nicht vorhanden:** Kreuzen Sie bei Häufigkeit „0“ an und gehen Sie zum nächsten Item.

**Falls vorhanden, fragen Sie weiter:**

| **26.1 Häufigkeit** | | | | |
| --- | --- | --- | --- | --- |
| **Spezifische Fragen zur Häufigkeit:** „Wie oft haben Sie sich hoffnungsvoll oder zuversichtlich gefühlt?“ | | | | |
| **Notizen:** | | | | |
| **Bitte schätzen Sie die Häufigkeit ein:** | | | | |
| 0  Nie | 1  Selten  (1-2-mal in den letzten beiden Wochen) | 2  Gelegentlich  (3-6-mal in den letzten beiden Wochen) | 3  Häufig  (ca. 7 -8-mal in den letzten beiden Wochen) | 4  Sehr häufig  (Fast täglich, oder durchgehend in den letzten beiden Wochen) |
| **26.2 Intensität** | | | | |
| **Spezifische Fragen und Beispiele zur Intensität:** „Wenn Sie das Gefühl der Zuversicht und Hoffnung verspürten – wie intensiv oder wie stark spürten Sie dies?“ | | | | |
| **Notizen:** | | | | |
| **Bitte schätzen Sie die ein:** | | | | |
| 0  Keine Hoffnung oder Zuversicht | 1  Flüchtige  Hoffnung und Zuversicht | 2  Deutliche Hoffnung und Zuversicht | 3  Stark ausgeprägte Hoffnung | 4  Intensiv ausgeprägte Zuversicht und Hoffnung |

**27) Sinnerfülltheit**

**Wörtliche Fragestellung:** „Gab es in der letzten beiden Wochen Momente, längere Episoden oder auch durchgängige Phasen, in denen Sie das Gefühl hatten, ein sinnerfülltes Leben zu führen? Das Gefühl, für jemanden oder für etwas von Bedeutung zu sein? Das Gefühl ein paar Dinge umzusetzen, die Ihnen wichtig sind?“

**Falls nicht vorhanden:**  Kreuzen Sie bei Häufigkeit „0“ an und gehen Sie zum nächsten Item.

**Falls vorhanden, fragen Sie weiter:**

| **27.1 Häufigkeit** | | | | |
| --- | --- | --- | --- | --- |
| **Spezifische Fragen zur Häufigkeit: „**Wie häufig hatten Sie dieses Gefühl der Sinnerfülltheit?“ | | | | |
| **Notizen:** | | | | |
| **Bitte schätzen Sie die Häufigkeit ein:** | | | | |
| 0  Nie | 1  Selten  (1-2-mal in den letzten beiden Wochen) | 2  Gelegentlich  (3-6-mal in den letzten beiden Wochen) | 3  Häufig  (ca. 7 -8-mal in den letzten beiden Wochen) | 4  Sehr häufig  (Fast täglich, oder durchgehend in den letzten beiden Wochen) |
| **27.2 Intensität** | | | | |
| **Spezifische Fragen und Beispiele zur Intensität: „**Wenn Sie das Gefühl hatten, ein sinnerfülltes Leben zu führen, wie intensiv oder wie stark spürten Sie dies? | | | | |
| **Notizen:** | | | | |
| **Bitte schätzen Sie die Intensität ein:** | | | | |
| 0  Kein Gefühl der Sinnerfülltheit | 1  Flüchtiges Gefühl der Sinnerfülltheit | 2  Deutliches Gefühl der Sinnerfülltheit | 3  Starkes Gefühl der Sinnerfülltheit | 4  Intensives Gefühl der Sinnerfülltheit |

**28) Lebenszufriedenheit**

**Wörtliche Fragestellung:** „Gab es in der letzten beiden Wochen Momente, längere Episoden oder auch durchgängige Phasen, in denen Sie das das Gefühl hatten, einverstanden mit Ihrem Leben im jetzigen Zustand oder gar mit Ihrem Leben zufrieden zu sein? Gab es Dinge, die Sie auf keinen Fall ändern wollten?

**Falls nicht vorhanden:** Kreuzen Sie bei Häufigkeit „0“ an und gehen Sie zum nächsten Item.

**Falls vorhanden, fragen Sie weiter:**

| **28.1 Häufigkeit** | | | | |
| --- | --- | --- | --- | --- |
| **Spezifische Fragen zur Häufigkeit: „**Wie häufig hatten Sie dieses Gefühl der Lebenszufriedenheit?“ | | | | |
| **Notizen:** | | | | |
| **Bitte schätzen Sie die Häufigkeit ein:** | | | | |
| 0  Nie | 1  Selten  (1-2-mal in den letzten beiden Wochen) | 2  Gelegentlich  (3-6-mal in den letzten beiden Wochen) | 3  Häufig  (ca. 7 -8-mal in den letzten beiden Wochen) | 4  Sehr häufig  (Fast täglich, oder durchgehend in den letzten beiden Wochen) |
| **28.2 Intensität** | | | | |
| **Spezifische Fragen und Beispiele zur Intensität**: „Wie intensiv oder wie stark spürten diese Gefühle?“ | | | | |
| **Notizen:** | | | | |
| **Bitte schätzen Sie die Intensität ein:** | | | | |
| 0  Kein Gefühl der Zufriedenheit | 1  Flüchtiges Gefühl der Zufriedenheit | 2  Deutliches Gefühl der Zufriedenheit | 3  Starkes Gefühl der Zufriedenheit | 4  Intensives Gefühl der Zufriedenheit |

**29) Freude oder Glück**

**Wörtliche Fragestellung:** „Gab es in der letzten beiden Wochen Momente, längere Episoden oder auch durchgängige Phasen, in denen Sie Freude oder Glück erleben konnten?“

**Falls nicht vorhanden:** Kreuzen Sie bei Häufigkeit „0“ an und gehen Sie zum nächsten Item.

**Falls vorhanden, fragen Sie weiter: „**Können Sie diese Momente genauer beschreiben? Waren dies eher Momente von Heiterkeit oder wie tief empfundenes Glück?“

| **29.1 Häufigkeit** | | | | |
| --- | --- | --- | --- | --- |
| **Spezifische Fragen zur Häufigkeit: „**Wie häufig hatten Sie dieses Gefühl von Freude oder Glück?“ | | | | |
| **Notizen:** | | | | |
| **Bitte schätzen Sie die Häufigkeit ein:** | | | | |
| 0  Nie | 1  Selten  (1-2-mal in den letzten beiden Wochen) | 2  Gelegentlich  (3-6-mal in den letzten beiden Wochen) | 3  Häufig  (ca. 7 -8-mal in den letzten beiden Wochen) | 4  Sehr häufig  (Fast täglich, oder durchgehend in den letzten beiden Wochen) |
| **29.2 Intensität** | | | | |
| **Spezifische Fragen und Beispiele zur Intensität**: „Wenn Sie diese Gefühle von Freude oder Glück hatten – wie intensiv oder wie stark spürten Sie dies?“ | | | | |
| **Notizen:** | | | | |
| **Bitte schätzen Sie die Intensität ein:** | | | | |
| 0  Kein Gefühl von Freude oder Glück | 1  Flüchtiges Gefühl von Freude oder Glück | 2  Deutliches Gefühl von Freude oder Glück | 3  Starkes Gefühl von Freude oder Glück | 4  Intensives Gefühl von Freude oder Glück |

**30) Geborgenheit**

**Wörtliche Fragestellung:** „Gab es in der letzten beiden Wochen Momente, längere Episoden oder auch durchgängige Phasen, in denen Sie wohltuende Geborgenheit erleben konnten?“

**Falls nicht vorhanden:** Kreuzen Sie bei Häufigkeit „0“ an und gehen Sie zum nächsten Item.

**Falls vorhanden, fragen Sie weiter:** „Können Sie diese Momente genauer beschreiben? Waren dies Erlebnisse mit anderen Personen, mit Tieren, mit einem höheren Wesen, oder auch einfach in der Natur oder mit Ihnen selbst?“

| **30.1 Häufigkeit** | | | | |
| --- | --- | --- | --- | --- |
| **Spezifische Fragen zur Häufigkeit: „**Falls ja, wie häufig hatten Sie dieses Gefühl von Geborgenheit?“ | | | | |
| **Notizen:** | | | | |
| **Bitte schätzen Sie die Häufigkeit ein:** | | | | |
| 0  Nie | 1  Selten  (1-2-mal in den letzten beiden Wochen) | 2  Gelegentlich  (3-6-mal in den letzten beiden Wochen) | 3  Häufig  (ca. 7 -8-mal in den letzten beiden Wochen) | 4  Sehr häufig  (Fast täglich, oder durchgehend in den letzten beiden Wochen) |
| **30.2 Intensität** | | | | |
| **Spezifische Fragen und Beispiele zur Intensität**: „Wenn Sie dieses Gefühl von Geborgenheit hatten - wie intensiv oder wie stark spürten Sie dies?“ | | | | |
| **Notizen:** | | | | |
| **Bitte schätzen Sie die Intensität ein:** | | | | |
| 0  Kein Gefühl der Geborgenheit | 1  Flüchtiges Gefühl von Geborgenheit | 2  Deutliches Gefühl von Geborgenheit | 3  Starkes Gefühl von Geborgenheit | 4  Intensives Gefühl von Geborgenheit |

**31) Beeinträchtigung im Alltag**

**Wörtliche Fragestellung:** „Wenn Sie nun noch einmal an alle Ihre Symptome denken, oder auch an Ihre Angst vor diesen Symptomen: Wie stark und wie häufig beeinträchtigten diese Ihren Alltag? Das heißt Ihr Berufsleben (Ausbildung; Schule), Ihre sozialen Beziehungen (Partnerschaft, Freunde), oder auch Ihre einfachen Angelegenheiten im Alltag, (sich um die Kinder kümmern, Aufstehen, Einkaufen, sauber machen).“

| **31.1 Alltagspraktische Fähigkeiten** | | | | |
| --- | --- | --- | --- | --- |
| **„Zunächst zu den einfachen Angelegenheiten im Alltag:** Gab es auf Grund Ihrer Symptome oder auch wegen der Angst vor diesen Symptomen, diesbezüglich Einschränkungen? Konnten Sie aufstehen, das Haus verlassen, einkaufen gehen, sich um Ihren Haushalt oder Ihre Kinder, Haustiere oder andere Angehörige (falls Sie welche haben) kümmern?“ | | | | |
| **Notizen:** | | | | |
| **Bitte schätzen Sie die Schwere und die Häufigkeit der Beeinträchtigung ein:** | | | | |
| 0  Keine Beeinträchtigung | 1  Geringe Beeinträchtigung: einmalig, oder wenige Tage | 2  Deutliche Beeinträchtigung: einige Tage | 3  Ausgeprägte Beeinträchtigung: überwiegende Zahl der Tage | 4  Schwere Beeinträchtigung: durchgehend |
| **31.2 Soziale Kontakte** | | | | |
| **„Nun zu Ihren sozialen Kontakten, also Partnern, Freunden, Nachbarn:** Gab es auf Grund Ihrer Symptome oder auch wegen der Angst vor diesen Symptomen, diesbezüglich Einschränkungen?  Gab es Schwierigkeiten mit Ihrem Partner / Partnerin? Konnten Sie sich mit Ihren Freunden / Freundinnen ungestört treffen, oder gab es Schwierigkeiten? Konnten Sie sich mit NachbarInnen oder KollegInnen treffen, oder gab es Schwierigkeiten? | | | | |
| **Notizen:** | | | | |
| **Bitte schätzen Sie die Schwere und die Häufigkeit der Beeinträchtigung ein:** | | | | |
| 0  Keine Beeinträchtigung | 1  Geringe Beeinträchtigung: einmalig, oder wenige Tage | 2  Deutliche Beeinträchtigung: einige Tage | 3  Ausgeprägte Beeinträchtigung: überwiegende Zahl der Tage | 4  Schwere Beeinträchtigung: durchgehend |
| **31.3 Beruf / Ausbildung / Schule** | | | | |
| **„Nun zu Ihrer beruflichen Situation (Ihrer Ausbildung; Ihrer schulischen Situation):** Gab es auf Grund Ihrer Symptome oder auch wegen der Angst vor diesen Symptomen, diesbezüglich Einschränkungen? Hatten Sie z.B. Streit, konnten Sie nicht hingehen, konnten Sie die erforderlichen Leistungen nicht erbringen?“ | | | | |
| **Notizen:** | | | | |
| **Bitte schätzen Sie die Schwere und die Häufigkeit der Beeinträchtigung ein:** | | | | |
| 0  Keine Beeinträchtigung | 1  Geringe Beeinträchtigung: einmalig, oder wenige Tage | 2  Deutliche Beeinträchtigung: einige Tage | 3  Ausgeprägte Beeinträchtigung: überwiegende Zahl der Tage | 4  Schwere Beeinträchtigung: durchgehend |
