# Supplementary figures and images for: The Borderline Symptom List–Interview: development and psychometric evaluation of an observer-based instrument for assessing symptom severity in borderline personality disorder
Source: Borderline Personal Disord Emot Dysregul. 2025 Aug 28;12:33. doi: 10.1186/s40479-025-00310-6 (PMC12395751; doi:10.1186/s40479-025-00310-6)

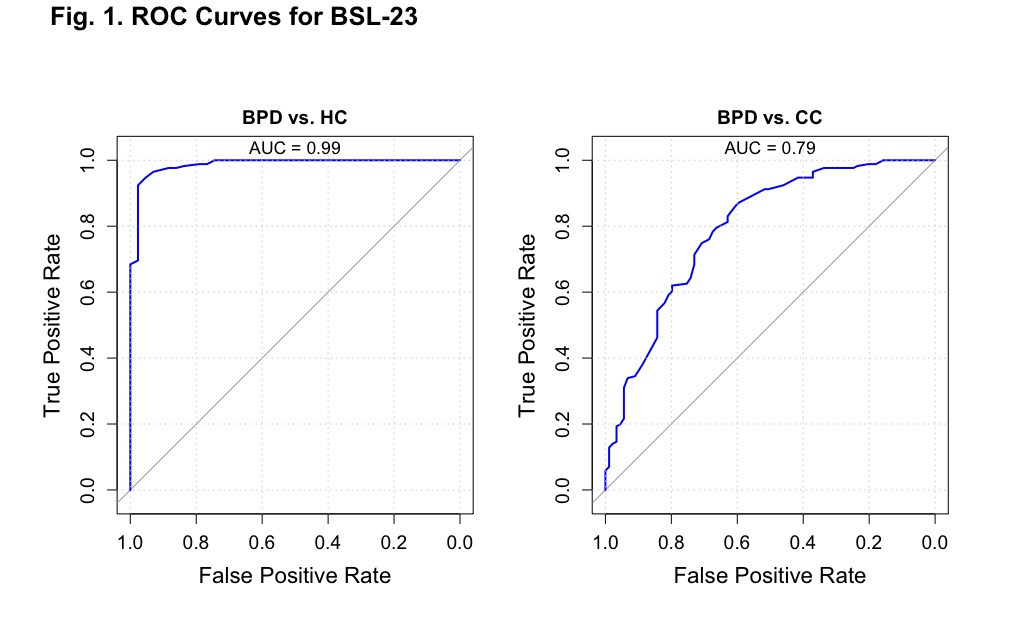

Supplement: Supplementary file 5 — Supplementary Material 5 [file 40479_2025_310_MOESM5_ESM.jpeg]
