# Supplementary material for: The Borderline Symptom List–Interview: development and psychometric evaluation of an observer-based instrument for assessing symptom severity in borderline personality disorder
Source: Borderline Personal Disord Emot Dysregul. 2025 Aug 28;12:33. doi: 10.1186/s40479-025-00310-6 (PMC12395751; doi:10.1186/s40479-025-00310-6)
Supplement: Supplementary file 6 — Supplementary Material 6 [file 40479_2025_310_MOESM6_ESM.docx]

# Supplements - Table 1

# Youden’s Index and Coordinates of the ROC curve for BPD vs CC

| Positive if Greater Than or Equal To | Sensitivity | Specificity | Youden |
| --- | --- | --- | --- |
| -Inf | 1.000 | 0.000 | 1.000 |
| 0.1125 | 1.000 | 0.011 | 1.011 |
| 0.1937 | 1.000 | 0.022 | 1.022 |
| 0.2146 | 1.000 | 0.034 | 1.034 |
| 0.2333 | 1.000 | 0.045 | 1.045 |
| 0.2542 | 1.000 | 0.056 | 1.056 |
| 0.2604 | 1.000 | 0.067 | 1.067 |
| 0.2708 | 1.000 | 0.079 | 1.079 |
| 0.2840 | 1.000 | 0.090 | 1.090 |
| 0.3111 | 1.000 | 0.101 | 1.101 |
| 0.3521 | 1.000 | 0.112 | 1.112 |
| 0.3708 | 1.000 | 0.124 | 1.124 |
| 0.3729 | 1.000 | 0.135 | 1.135 |
| 0.3771 | 1.000 | 0.146 | 1.146 |
| 0.3812 | 0.994 | 0.146 | 1.140 |
| 0.3958 | 0.994 | 0.169 | 1.163 |
| 0.4333 | 0.994 | 0.180 | 1.174 |
| 0.4625 | 0.994 | 0.191 | 1.185 |
| 0.4729 | 0.994 | 0.202 | 1.196 |
| 0.4812 | 0.994 | 0.213 | 1.208 |
| 0.4875 | 0.994 | 0.225 | 1.219 |
| 0.5083 | 0.994 | 0.236 | 1.230 |
| 0.5396 | 0.994 | 0.247 | 1.241 |
| 0.5646 | 0.994 | 0.258 | 1.253 |
| 0.5792 | 0.994 | 0.270 | 1.264 |
| 0.5896 | 0.994 | 0.281 | 1.275 |
| 0.5979 | 0.994 | 0.292 | 1.286 |
| 0.6042 | 0.988 | 0.303 | 1.292 |
| 0.6167 | 0.988 | 0.326 | 1.314 |
| 0.6479 | 0.982 | 0.326 | 1.308 |
| 0.6771 | 0.982 | 0.337 | 1.320 |
| 0.6854 | 0.982 | 0.348 | 1.331 |
| 0.7000 | 0.977 | 0.348 | 1.325 |
| 0.7146 | 0.977 | 0.360 | 1.336 |
| 0.7229 | 0.977 | 0.371 | 1.347 |
| 0.7333 | 0.977 | 0.382 | 1.359 |
| 0.7458 | 0.977 | 0.393 | 1.370 |
| 0.7604 | 0.977 | 0.404 | 1.381 |
| 0.7688 | 0.977 | 0.416 | 1.392 |
| 0.7750 | 0.977 | 0.427 | 1.404 |
| 0.7854 | 0.977 | 0.449 | 1.426 |
| 0.7979 | 0.977 | 0.461 | 1.437 |
| 0.8250 | 0.977 | 0.472 | 1.449 |
| 0.8479 | 0.977 | 0.483 | 1.460 |
| 0.8508 | 0.977 | 0.494 | 1.471 |
| 0.8529 | 0.977 | 0.506 | 1.482 |
| 0.8684 | 0.977 | 0.517 | 1.493 |
| 0.8827 | 0.971 | 0.517 | 1.488 |
| 0.8894 | 0.971 | 0.528 | 1.499 |
| 0.8979 | 0.965 | 0.528 | 1.493 |
| 0.9007 | 0.959 | 0.528 | 1.487 |
| 0.9028 | 0.959 | 0.539 | 1.498 |
| 0.9188 | 0.959 | 0.551 | 1.510 |
| 0.9479 | 0.959 | 0.562 | 1.521 |
| 0.9658 | 0.953 | 0.562 | 1.515 |
| 0.9700 | 0.953 | 0.573 | 1.526 |
| 0.9792 | 0.947 | 0.573 | 1.520 |
| 0.9917 | 0.947 | 0.584 | 1.532 |
| 0.9958 | 0.947 | 0.596 | 1.543 |
| 1.0021 | 0.947 | 0.607 | 1.554 |
| 1.0167 | 0.947 | 0.618 | 1.565 |
| 1.0292 | 0.947 | 0.629 | 1.577 |
| 1.0333 | 0.947 | 0.640 | 1.588 |
| 1.0583 | 0.947 | 0.652 | 1.599 |
| 1.0875 | 0.942 | 0.652 | 1.593 |
| 1.0932 | 0.942 | 0.663 | 1.604 |
| 1.0974 | 0.936 | 0.663 | 1.599 |
| 1.1063 | 0.930 | 0.663 | 1.593 |
| 1.1208 | 0.924 | 0.663 | 1.587 |
| 1.1396 | 0.924 | 0.674 | 1.598 |
| 1.1542 | 0.924 | 0.685 | 1.609 |
| 1.1646 | 0.918 | 0.685 | 1.604 |
| 1.1729 | 0.918 | 0.697 | 1.615 |
| 1.1854 | 0.918 | 0.708 | 1.626 |
| 1.1979 | 0.918 | 0.730 | 1.648 |
| 1.2021 | 0.906 | 0.730 | 1.637 |
| 1.2068 | 0.901 | 0.742 | 1.642 |
| 1.2234 | 0.895 | 0.742 | 1.636 |
| 1.2396 | 0.895 | 0.753 | 1.648 |
| 1.2500 | 0.895 | 0.764 | 1.659 |
| 1.2625 | 0.895 | 0.775 | 1.670 |
| 1.2750 | 0.895 | 0.787 | 1.681 |
| 1.3000 | 0.883 | 0.787 | 1.670 |
| 1.3292 | 0.883 | 0.798 | 1.681 |
| 1.3458 | 0.877 | 0.798 | 1.675 |
| 1.3521 | 0.877 | 0.809 | 1.686 |
| 1.3687 | 0.877 | 0.820 | 1.697 |
| 1.3938 | 0.871 | 0.820 | 1.692 |
| 1.4062 | 0.865 | 0.820 | 1.686 |
| 1.4146 | 0.860 | 0.820 | 1.680 |
| 1.4292 | 0.860 | 0.831 | 1.691 |
| 1.4438 | 0.854 | 0.831 | 1.685 |
| 1.4521 | 0.848 | 0.831 | 1.679 |
| 1.4561 | 0.848 | 0.843 | 1.691 |
| 1.4603 | 0.842 | 0.843 | 1.685 |
| 1.4688 | 0.842 | 0.854 | 1.696 |
| 1.4792 | 0.836 | 0.854 | 1.690 |
| 1.4917 | 0.830 | 0.854 | 1.684 |
| 1.5000 | 0.830 | 0.865 | 1.696 |
| 1.5021 | 0.825 | 0.865 | 1.690 |
| 1.5083 | 0.819 | 0.876 | 1.695 |
| 1.5167 | 0.813 | 0.888 | 1.701 |
| 1.5312 | 0.807 | 0.888 | 1.695 |
| 1.5500 | 0.801 | 0.899 | 1.700 |
| 1.5583 | 0.795 | 0.899 | 1.694 |
| 1.5625 | 0.789 | 0.899 | 1.688 |
| 1.5667 | 0.778 | 0.899 | 1.677 |
| 1.5688 | 0.772 | 0.899 | 1.671 |
| 1.5750 | 0.766 | 0.899 | 1.665 |
| 1.5958 | 0.760 | 0.899 | 1.659 |
| 1.6146 | 0.754 | 0.899 | 1.653 |
| 1.6250 | 0.749 | 0.899 | 1.647 |
| 1.6333 | 0.743 | 0.899 | 1.642 |
| 1.6375 | 0.743 | 0.910 | 1.653 |
| 1.6437 | 0.737 | 0.910 | 1.647 |
| 1.6479 | 0.731 | 0.910 | 1.641 |
| 1.6542 | 0.725 | 0.910 | 1.635 |
| 1.6667 | 0.719 | 0.910 | 1.629 |
| 1.6833 | 0.713 | 0.910 | 1.624 |
| 1.6938 | 0.708 | 0.910 | 1.618 |
| 1.6979 | 0.702 | 0.910 | 1.612 |
| 1.7000 | 0.696 | 0.910 | 1.606 |
| 1.7021 | 0.690 | 0.910 | 1.600 |
| 1.7083 | 0.678 | 0.910 | 1.588 |
| 1.7167 | 0.673 | 0.910 | 1.583 |
| 1.7250 | 0.673 | 0.921 | 1.594 |
| 1.7312 | 0.667 | 0.921 | 1.588 |
| 1.7375 | 0.655 | 0.921 | 1.576 |
| 1.7481 | 0.649 | 0.921 | 1.570 |
| 1.7564 | 0.643 | 0.921 | 1.565 |
| 1.7625 | 0.637 | 0.921 | 1.559 |
| 1.7750 | 0.637 | 0.933 | 1.570 |
| 1.7875 | 0.632 | 0.933 | 1.564 |
| 1.7937 | 0.626 | 0.933 | 1.558 |
| 1.7979 | 0.620 | 0.933 | 1.552 |
| 1.8021 | 0.614 | 0.944 | 1.558 |
| 1.8062 | 0.614 | 0.955 | 1.569 |
| 1.8104 | 0.608 | 0.955 | 1.563 |
| 1.8146 | 0.596 | 0.955 | 1.552 |
| 1.8264 | 0.591 | 0.955 | 1.546 |
| 1.8368 | 0.585 | 0.955 | 1.540 |
| 1.8417 | 0.579 | 0.955 | 1.534 |
| 1.8542 | 0.579 | 0.966 | 1.545 |
| 1.8625 | 0.573 | 0.966 | 1.539 |
| 1.8708 | 0.567 | 0.966 | 1.534 |
| 1.8803 | 0.561 | 0.966 | 1.528 |
| 1.8845 | 0.556 | 0.966 | 1.522 |
| 1.8917 | 0.544 | 0.966 | 1.510 |
| 1.9021 | 0.538 | 0.966 | 1.504 |
| 1.9104 | 0.532 | 0.966 | 1.498 |
| 1.9125 | 0.532 | 0.978 | 1.510 |
| 1.9146 | 0.526 | 0.978 | 1.504 |
| 1.9208 | 0.520 | 0.978 | 1.498 |
| 1.9271 | 0.515 | 0.978 | 1.492 |
| 1.9401 | 0.509 | 0.978 | 1.486 |
| 1.9568 | 0.503 | 0.978 | 1.480 |
| 1.9660 | 0.497 | 0.978 | 1.475 |
| 1.9698 | 0.491 | 0.978 | 1.469 |
| 1.9726 | 0.485 | 0.978 | 1.463 |
| 1.9792 | 0.480 | 0.978 | 1.457 |
| 1.9875 | 0.474 | 0.978 | 1.451 |
| 1.9937 | 0.450 | 0.978 | 1.428 |
| 1.9979 | 0.444 | 0.978 | 1.422 |
| 2.0021 | 0.444 | 0.989 | 1.433 |
| 2.0053 | 0.439 | 0.989 | 1.427 |
| 2.0073 | 0.433 | 0.989 | 1.422 |
| 2.0083 | 0.427 | 0.989 | 1.416 |
| 2.0125 | 0.421 | 0.989 | 1.410 |
| 2.0194 | 0.415 | 0.989 | 1.404 |
| 2.0298 | 0.409 | 0.989 | 1.398 |
| 2.0417 | 0.404 | 0.989 | 1.392 |
| 2.0479 | 0.398 | 0.989 | 1.386 |
| 2.0521 | 0.392 | 0.989 | 1.381 |
| 2.0560 | 0.386 | 0.989 | 1.375 |
| 2.0662 | 0.380 | 0.989 | 1.369 |
| 2.0789 | 0.374 | 0.989 | 1.363 |
| 2.0854 | 0.368 | 0.989 | 1.357 |
| 2.0917 | 0.363 | 0.989 | 1.351 |
| 2.0985 | 0.357 | 1.000 | 1.357 |
| 2.1048 | 0.351 | 1.000 | 1.351 |
| 2.1104 | 0.345 | 1.000 | 1.345 |
| 2.1146 | 0.327 | 1.000 | 1.327 |
| 2.1188 | 0.322 | 1.000 | 1.322 |
| 2.1250 | 0.316 | 1.000 | 1.316 |
| 2.1312 | 0.310 | 1.000 | 1.310 |
| 2.1437 | 0.304 | 1.000 | 1.304 |
| 2.1583 | 0.298 | 1.000 | 1.298 |
| 2.1646 | 0.292 | 1.000 | 1.292 |
| 2.1708 | 0.281 | 1.000 | 1.281 |
| 2.1792 | 0.275 | 1.000 | 1.275 |
| 2.1854 | 0.269 | 1.000 | 1.269 |
| 2.1896 | 0.257 | 1.000 | 1.257 |
| 2.1958 | 0.251 | 1.000 | 1.251 |
| 2.2021 | 0.246 | 1.000 | 1.246 |
| 2.2062 | 0.240 | 1.000 | 1.240 |
| 2.2104 | 0.234 | 1.000 | 1.234 |
| 2.2142 | 0.228 | 1.000 | 1.228 |
| 2.2184 | 0.222 | 1.000 | 1.222 |
| 2.2250 | 0.216 | 1.000 | 1.216 |
| 2.2333 | 0.211 | 1.000 | 1.211 |
| 2.2583 | 0.205 | 1.000 | 1.205 |
| 2.2812 | 0.199 | 1.000 | 1.199 |
| 2.2917 | 0.193 | 1.000 | 1.193 |
| 2.3062 | 0.187 | 1.000 | 1.187 |
| 2.3167 | 0.175 | 1.000 | 1.175 |
| 2.3271 | 0.170 | 1.000 | 1.170 |
| 2.3417 | 0.164 | 1.000 | 1.164 |
| 2.3585 | 0.158 | 1.000 | 1.158 |
| 2.3731 | 0.152 | 1.000 | 1.152 |
| 2.3958 | 0.146 | 1.000 | 1.146 |
| 2.4146 | 0.140 | 1.000 | 1.140 |
| 2.4292 | 0.135 | 1.000 | 1.135 |
| 2.4542 | 0.129 | 1.000 | 1.129 |
| 2.4813 | 0.123 | 1.000 | 1.123 |
| 2.5062 | 0.117 | 1.000 | 1.117 |
| 2.5208 | 0.111 | 1.000 | 1.111 |
| 2.5292 | 0.105 | 1.000 | 1.105 |
| 2.5354 | 0.099 | 1.000 | 1.099 |
| 2.5458 | 0.088 | 1.000 | 1.088 |
| 2.5623 | 0.082 | 1.000 | 1.082 |
| 2.5706 | 0.076 | 1.000 | 1.076 |
| 2.5812 | 0.070 | 1.000 | 1.070 |
| 2.5958 | 0.064 | 1.000 | 1.064 |
| 2.6063 | 0.053 | 1.000 | 1.053 |
| 2.6479 | 0.047 | 1.000 | 1.047 |
| 2.6917 | 0.041 | 1.000 | 1.041 |
| 2.7042 | 0.035 | 1.000 | 1.035 |
| 2.7562 | 0.029 | 1.000 | 1.029 |
| 2.8542 | 0.023 | 1.000 | 1.023 |
| 2.9313 | 0.018 | 1.000 | 1.018 |
| 3.0125 | 0.012 | 1.000 | 1.012 |
| 3.0750 | 0.006 | 1.000 | 1.006 |
| Inf | 0.000 | 1.000 | 1.000 |

# Supplementents Table 2

# Youden’s Index and Coordinates of the ROC curve for BPD vs HC

| Positive if Greater Than or Equal  To | Sensitivity | Specificity | Youden |
| --- | --- | --- | --- |
| -Inf | 1.000 | 0.000 | 1.000 |
| 0.0125 | 1.000 | 0.047 | 1.047 |
| 0.0333 | 1.000 | 0.070 | 1.070 |
| 0.0458 | 1.000 | 0.093 | 1.093 |
| 0.0542 | 1.000 | 0.116 | 1.116 |
| 0.0625 | 1.000 | 0.163 | 1.163 |
| 0.0688 | 1.000 | 0.186 | 1.186 |
| 0.0729 | 1.000 | 0.209 | 1.209 |
| 0.0792 | 1.000 | 0.256 | 1.256 |
| 0.0875 | 1.000 | 0.302 | 1.302 |
| 0.0938 | 1.000 | 0.326 | 1.326 |
| 0.0979 | 1.000 | 0.349 | 1.349 |
| 0.1042 | 1.000 | 0.395 | 1.395 |
| 0.1125 | 1.000 | 0.419 | 1.419 |
| 0.1208 | 1.000 | 0.442 | 1.442 |
| 0.1292 | 1.000 | 0.488 | 1.488 |
| 0.1354 | 1.000 | 0.512 | 1.512 |
| 0.1396 | 1.000 | 0.535 | 1.535 |
| 0.1562 | 1.000 | 0.558 | 1.558 |
| 0.1729 | 1.000 | 0.581 | 1.581 |
| 0.1771 | 1.000 | 0.605 | 1.605 |
| 0.1854 | 1.000 | 0.628 | 1.628 |
| 0.2021 | 1.000 | 0.674 | 1.674 |
| 0.2146 | 1.000 | 0.698 | 1.698 |
| 0.2250 | 1.000 | 0.721 | 1.721 |
| 0.2375 | 1.000 | 0.744 | 1.744 |
| 0.2458 | 1.000 | 0.767 | 1.767 |
| 0.2583 | 1.000 | 0.791 | 1.791 |
| 0.2750 | 1.000 | 0.814 | 1.814 |
| 0.3167 | 1.000 | 0.837 | 1.837 |
| 0.3542 | 1.000 | 0.860 | 1.860 |
| 0.3667 | 1.000 | 0.884 | 1.884 |
| 0.3771 | 1.000 | 0.907 | 1.907 |
| 0.3833 | 0.994 | 0.907 | 1.901 |
| 0.3979 | 0.994 | 0.930 | 1.924 |
| 0.5042 | 0.994 | 0.953 | 1.948 |
| 0.6125 | 0.988 | 0.953 | 1.942 |
| 0.6562 | 0.982 | 0.977 | 1.959 |
| 0.7850 | 0.977 | 1.000 | 1.977 |
| 0.8892 | 0.971 | 1.000 | 1.971 |
| 0.8979 | 0.965 | 1.000 | 1.965 |
| 0.9313 | 0.959 | 1.000 | 1.959 |
| 0.9667 | 0.953 | 1.000 | 1.953 |
| 1.0271 | 0.947 | 1.000 | 1.947 |
| 1.0891 | 0.942 | 1.000 | 1.942 |
| 1.0974 | 0.936 | 1.000 | 1.936 |
| 1.1063 | 0.930 | 1.000 | 1.930 |
| 1.1354 | 0.924 | 1.000 | 1.924 |
| 1.1792 | 0.918 | 1.000 | 1.918 |
| 1.2021 | 0.906 | 1.000 | 1.906 |
| 1.2068 | 0.901 | 1.000 | 1.901 |
| 1.2463 | 0.895 | 1.000 | 1.895 |
| 1.3125 | 0.883 | 1.000 | 1.883 |
| 1.3625 | 0.877 | 1.000 | 1.877 |
| 1.3938 | 0.871 | 1.000 | 1.871 |
| 1.4062 | 0.865 | 1.000 | 1.865 |
| 1.4229 | 0.860 | 1.000 | 1.860 |
| 1.4438 | 0.854 | 1.000 | 1.854 |
| 1.4540 | 0.848 | 1.000 | 1.848 |
| 1.4665 | 0.842 | 1.000 | 1.842 |
| 1.4792 | 0.836 | 1.000 | 1.836 |
| 1.4917 | 0.830 | 1.000 | 1.830 |
| 1.5021 | 0.825 | 1.000 | 1.825 |
| 1.5083 | 0.819 | 1.000 | 1.819 |
| 1.5167 | 0.813 | 1.000 | 1.813 |
| 1.5312 | 0.807 | 1.000 | 1.807 |
| 1.5500 | 0.801 | 1.000 | 1.801 |
| 1.5583 | 0.795 | 1.000 | 1.795 |
| 1.5625 | 0.789 | 1.000 | 1.789 |
| 1.5667 | 0.778 | 1.000 | 1.778 |
| 1.5688 | 0.772 | 1.000 | 1.772 |
| 1.5750 | 0.766 | 1.000 | 1.766 |
| 1.5958 | 0.760 | 1.000 | 1.760 |
| 1.6146 | 0.754 | 1.000 | 1.754 |
| 1.6250 | 0.749 | 1.000 | 1.749 |
| 1.6375 | 0.743 | 1.000 | 1.743 |
| 1.6437 | 0.737 | 1.000 | 1.737 |
| 1.6479 | 0.731 | 1.000 | 1.731 |
| 1.6542 | 0.725 | 1.000 | 1.725 |
| 1.6667 | 0.719 | 1.000 | 1.719 |
| 1.6833 | 0.713 | 1.000 | 1.713 |
| 1.6938 | 0.708 | 1.000 | 1.708 |
| 1.6979 | 0.702 | 1.000 | 1.702 |
| 1.7000 | 0.696 | 1.000 | 1.696 |
| 1.7021 | 0.690 | 1.000 | 1.690 |
| 1.7083 | 0.678 | 1.000 | 1.678 |
| 1.7208 | 0.673 | 1.000 | 1.673 |
| 1.7312 | 0.667 | 1.000 | 1.667 |
| 1.7375 | 0.655 | 1.000 | 1.655 |
| 1.7481 | 0.649 | 1.000 | 1.649 |
| 1.7564 | 0.643 | 1.000 | 1.643 |
| 1.7708 | 0.637 | 1.000 | 1.637 |
| 1.7875 | 0.632 | 1.000 | 1.632 |
| 1.7937 | 0.626 | 1.000 | 1.626 |
| 1.7979 | 0.620 | 1.000 | 1.620 |
| 1.8042 | 0.614 | 1.000 | 1.614 |
| 1.8104 | 0.608 | 1.000 | 1.608 |
| 1.8146 | 0.596 | 1.000 | 1.596 |
| 1.8264 | 0.591 | 1.000 | 1.591 |
| 1.8368 | 0.585 | 1.000 | 1.585 |
| 1.8500 | 0.579 | 1.000 | 1.579 |
| 1.8625 | 0.573 | 1.000 | 1.573 |
| 1.8708 | 0.567 | 1.000 | 1.567 |
| 1.8803 | 0.561 | 1.000 | 1.561 |
| 1.8845 | 0.556 | 1.000 | 1.556 |
| 1.8917 | 0.544 | 1.000 | 1.544 |
| 1.9021 | 0.538 | 1.000 | 1.538 |
| 1.9104 | 0.532 | 1.000 | 1.532 |
| 1.9146 | 0.526 | 1.000 | 1.526 |
| 1.9208 | 0.520 | 1.000 | 1.520 |
| 1.9271 | 0.515 | 1.000 | 1.515 |
| 1.9401 | 0.509 | 1.000 | 1.509 |
| 1.9568 | 0.503 | 1.000 | 1.503 |
| 1.9660 | 0.497 | 1.000 | 1.497 |
| 1.9698 | 0.491 | 1.000 | 1.491 |
| 1.9726 | 0.485 | 1.000 | 1.485 |
| 1.9792 | 0.480 | 1.000 | 1.480 |
| 1.9875 | 0.474 | 1.000 | 1.474 |
| 1.9937 | 0.450 | 1.000 | 1.450 |
| 2.0000 | 0.444 | 1.000 | 1.444 |
| 2.0053 | 0.439 | 1.000 | 1.439 |
| 2.0073 | 0.433 | 1.000 | 1.433 |
| 2.0083 | 0.427 | 1.000 | 1.427 |
| 2.0125 | 0.421 | 1.000 | 1.421 |
| 2.0194 | 0.415 | 1.000 | 1.415 |
| 2.0298 | 0.409 | 1.000 | 1.409 |
| 2.0417 | 0.404 | 1.000 | 1.404 |
| 2.0479 | 0.398 | 1.000 | 1.398 |
| 2.0521 | 0.392 | 1.000 | 1.392 |
| 2.0560 | 0.386 | 1.000 | 1.386 |
| 2.0662 | 0.380 | 1.000 | 1.380 |
| 2.0789 | 0.374 | 1.000 | 1.374 |
| 2.0854 | 0.368 | 1.000 | 1.368 |
| 2.0917 | 0.363 | 1.000 | 1.363 |
| 2.0985 | 0.357 | 1.000 | 1.357 |
| 2.1048 | 0.351 | 1.000 | 1.351 |
| 2.1104 | 0.345 | 1.000 | 1.345 |
| 2.1146 | 0.327 | 1.000 | 1.327 |
| 2.1188 | 0.322 | 1.000 | 1.322 |
| 2.1250 | 0.316 | 1.000 | 1.316 |
| 2.1312 | 0.310 | 1.000 | 1.310 |
| 2.1437 | 0.304 | 1.000 | 1.304 |
| 2.1583 | 0.298 | 1.000 | 1.298 |
| 2.1646 | 0.292 | 1.000 | 1.292 |
| 2.1708 | 0.281 | 1.000 | 1.281 |
| 2.1792 | 0.275 | 1.000 | 1.275 |
| 2.1854 | 0.269 | 1.000 | 1.269 |
| 2.1896 | 0.257 | 1.000 | 1.257 |
| 2.1958 | 0.251 | 1.000 | 1.251 |
| 2.2021 | 0.246 | 1.000 | 1.246 |
| 2.2062 | 0.240 | 1.000 | 1.240 |
| 2.2104 | 0.234 | 1.000 | 1.234 |
| 2.2142 | 0.228 | 1.000 | 1.228 |
| 2.2184 | 0.222 | 1.000 | 1.222 |
| 2.2250 | 0.216 | 1.000 | 1.216 |
| 2.2333 | 0.211 | 1.000 | 1.211 |
| 2.2583 | 0.205 | 1.000 | 1.205 |
| 2.2812 | 0.199 | 1.000 | 1.199 |
| 2.2917 | 0.193 | 1.000 | 1.193 |
| 2.3062 | 0.187 | 1.000 | 1.187 |
| 2.3167 | 0.175 | 1.000 | 1.175 |
| 2.3271 | 0.170 | 1.000 | 1.170 |
| 2.3417 | 0.164 | 1.000 | 1.164 |
| 2.3585 | 0.158 | 1.000 | 1.158 |
| 2.3731 | 0.152 | 1.000 | 1.152 |
| 2.3958 | 0.146 | 1.000 | 1.146 |
| 2.4146 | 0.140 | 1.000 | 1.140 |
| 2.4292 | 0.135 | 1.000 | 1.135 |
| 2.4542 | 0.129 | 1.000 | 1.129 |
| 2.4813 | 0.123 | 1.000 | 1.123 |
| 2.5062 | 0.117 | 1.000 | 1.117 |
| 2.5208 | 0.111 | 1.000 | 1.111 |
| 2.5292 | 0.105 | 1.000 | 1.105 |
| 2.5354 | 0.099 | 1.000 | 1.099 |
| 2.5458 | 0.088 | 1.000 | 1.088 |
| 2.5623 | 0.082 | 1.000 | 1.082 |
| 2.5706 | 0.076 | 1.000 | 1.076 |
| 2.5812 | 0.070 | 1.000 | 1.070 |
| 2.5958 | 0.064 | 1.000 | 1.064 |
| 2.6063 | 0.053 | 1.000 | 1.053 |
| 2.6479 | 0.047 | 1.000 | 1.047 |
| 2.6917 | 0.041 | 1.000 | 1.041 |
| 2.7042 | 0.035 | 1.000 | 1.035 |
| 2.7562 | 0.029 | 1.000 | 1.029 |
| 2.8542 | 0.023 | 1.000 | 1.023 |
| 2.9313 | 0.018 | 1.000 | 1.018 |
| 3.0125 | 0.012 | 1.000 | 1.012 |
| 3.0750 | 0.006 | 1.000 | 1.006 |
| Inf | 0.000 | 1.000 | 1.000 |
